# A rational design strategy of radical-type mechanophores with thermal tolerance†

**DOI:** 10.1039/d3sc02991c

**Published:** 2023-08-03

**Authors:** Yi Lu, Hajime Sugita, Koichiro Mikami, Daisuke Aoki, Hideyuki Otsuka

**Affiliations:** a Department of Chemical Science and Engineering, Tokyo Institute of Technology 2-12-1 Ookayama, Meguro-ku Tokyo 152-8550 Japan; b Sagami Chemical Research Institute 2743-1 Hayakawa Ayase Kanagawa 252-1193 Japan r200870208tyoko@gmail.com; c Living Systems Materialogy (LiSM) Research Group, International Research Frontiers Initiative (IRFI), Tokyo Institute of Technology 4259 Nagatsuta-cho, Midori-ku Yokohama 226-8501 Japan

## Abstract

Radical-type mechanophores (RMs) are attractive molecules that undergo homolytic scission of their central C–C bond to afford radical species upon exposure to heat or mechanical stimuli. However, the lack of a rational design concept limits the development of RMs with pre-determined properties. Herein, we report a rational design strategy of RMs with high thermal tolerance while maintaining mechanoresponsiveness. A combined experimental and theoretical analysis revealed that the high thermal tolerance of these RMs is related to the radical-stabilization energy (RSE) as well as the Hammett and modified Swain–Lupton constants at the *para*-position (*σ*_p_). The trend of the RSE values is in good agreement with the experimentally evaluated thermal tolerance of a series of mechanoresponsive RMs based on the bisarylcyanoacetate motif. Furthermore, the singly occupied molecular orbital (SOMO) levels clearly exhibit a negative correlation with *σ*_p_ within a series of RMs that are based on the same skeleton, paving the way toward the development of RMs that can be handled under ambient conditions without peroxidation.

## Abstract 2

Radical-type mechanophores (RMs) are attractive molecules that undergo homolytic scission of their central C–C bond to afford radical species upon exposure to heat or mechanical stimuli.

## Graphical abstract

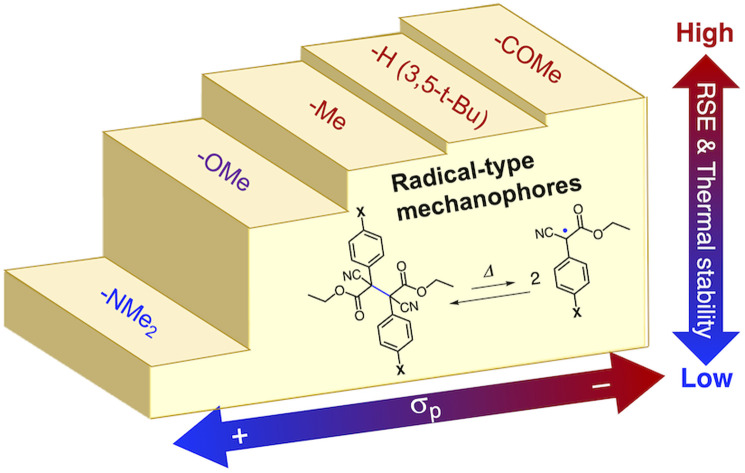

## Introduction

The development of mechanoresponsive materials (MRMs) that exhibit a variety of macroscale functionalities, including coloring, fluorescence, self-healing, and self-strengthening, upon exposure to mechanical stimuli has been a research hotspot in materials science during the past few decades.^1–12^ In particular, mechanophores that induce microscale chemical reactions such as scission, isomerization, and release of small molecules are essential because they enable the functionalization of polymeric materials. Radical-type mechanophores (RMs), which undergo mechanical-stimuli-induced homolytic scission of their central C–C bonds to afford radical species, have been used to study micro-to-macroscale mechanical stimuli, strengthened materials based on sacrificial bonds, or subsequent polymer propagation.^13–23^

The nature of the central C–C bond of RMs exerts a great impact on the functionality of the resulting polymeric materials. For example, the central C–C bonds of diarylbibenzofuranone (DABBF) and tetraarylsuccinonitrile (TASN) readily dissociate even at ambient temperature, which makes it possible to endow the polymeric material with self-healing properties.^18,24^ In contrast, the central C–C bond of difluorenylsuccinonitrile (DFSN), which exhibits the highest thermal tolerance among hitherto reported RMs, does not dissociate up to 100 °C.^22^ The extraordinary thermal tolerance of DFSN inspired us to apply the radical-polymerization methodology to develop highly mechanoresponsive or shape-memory materials.^15,25,26^ However, despite the clear importance of decreasing the propensity of the central C–C bond to subdue to thermal scission while preserving its ability to scission upon exposure to mechanical impact, the relationship between both phenomena is still unclear, which limits the scope of RM-based materials.

**Fig. 1:**
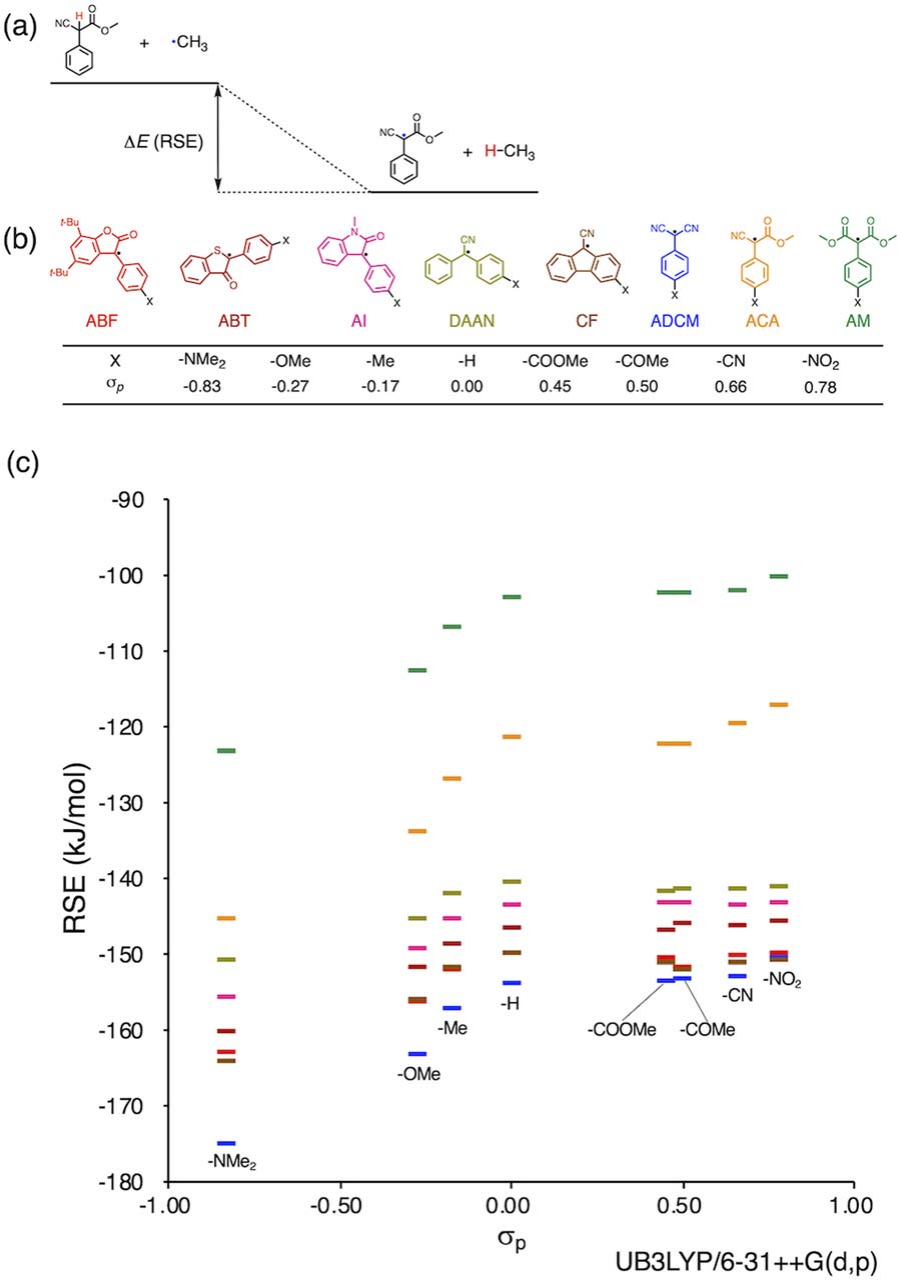
(a) Schematic illustration of RSE using ACA radical as an example; (b) radical skeletons with various functional groups and their *σ*_p_ values; (c) plot of RSE against *σ*_p_ (the colors in the plot correspond to those of (b)). DFT calculations were performed at the unrestricted B3LYP/6-31++G(d,p) level.

Herein, we report a rational design strategy for RMs with high thermal tolerance for the development of novel MRMs. We found that the thermal tolerance within a series of RMs that contain the same skeleton can be simply explained by the relationship between two key factors, *i.e.*, the radical-stabilization energy (RSE) (Fig. 1a)^27–31^ as well as the Hammett and modified Swain–Lupton constants at the *para*-position (*σ*_p_) (Fig. 1b).^32^ The RSE, which indicates the thermodynamic stability of radicals, is defined as the enthalpy change in isodesmic hydrogen-transfer reactions (Fig. 1a). We simulated the RSE values using a computational approach (Fig. 1c and S32–S34†). Meanwhile, *σ*_p_ is an experimental value that quantitatively represents the electron-withdrawing or electron-donating nature of substitution groups at *para*-position of the aromatic part (Fig. 1b).

## Results and discussion

We investigated the relationship between RSE and *σ*_p_ in a series of representative radical molecules including derivatives of arylbenzofuranone (ABF), arylbenzothiophenone (ABT), arylindolinone (AI), diarylacetonitrile (DAAN), cyanofluorene (CF), aryldicyanomethane (ADCM), arylcyanoacetate (ACA), and arylmalonate (AM). Dimers of ABF, ABT, AI, DAAN, and CF radicals are radical-type mechanophores previously developed in our group.^18,22,24,33,34^ In order to elucidate the electron-donating or -withdrawing nature of these radicals, not only ADCMs, which are well-known stable radicals under ambient conditions that possess two electron-withdrawing cyano groups,^35–45^ but also ACAs and AMs were investigated as ADCM analogues.^46,47^ The relationship between RSE and *σ*_p_ is plotted in Fig. 1c, which shows that the change in the RSE values within the series of radical molecules that have the same skeleton depends on the *σ*_p_ range. The RSE value clearly increases with increasing *σ*_p_ value in the region of *σ*_p_ < 0, where the electron-donating nature of the functional groups increases with decreasing *σ*_p_ value. In contrast, the RSE values did not show significant changes and almost saturated in the region of *σ*_p_ > 0, where the electron-withdrawing nature of the functional groups increases with increasing *σ*_p_ value. These trends were theoretically reproduced irrespective of the computational methodology used (Fig. S32–S34†). These results thus indicate that the thermodynamic stability of these radicals can be interpreted in terms of the substituent effect within a series of radicals that share the same skeleton. Therefore, we envisioned that it should be possible to develop RMs with high thermal tolerance by designing electron-deficient radical species that bear electron-withdrawing groups.

**Scheme 1:**
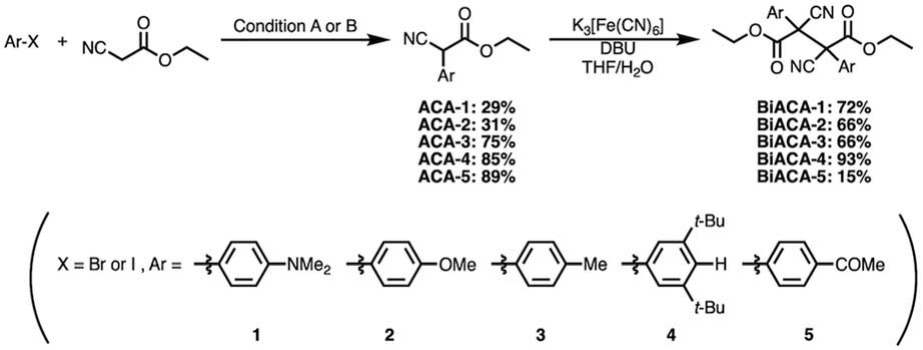
Synthesis of BiACAs with a variety of substituents at *para*-position of the aryl group. Reaction conditions: (A) Pd_2_(dba)_3_/HBF_4_·P(*t*-Bu)_3_, Na_3_PO_4_, toluene, 80 °C; (B) CuI/l-proline, K_2_CO_3_, DMSO, 90 °C.

**Fig. 2:**
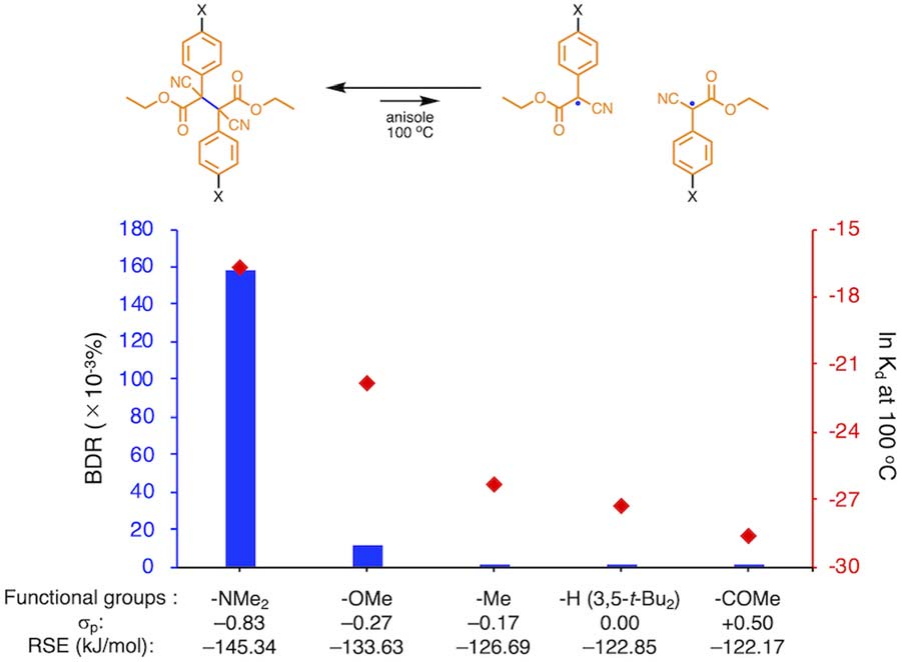
Bar graph and plot of BDR and ln *K*_d_ at 100 °C as a function of the increasing RSE and *σ*_p_ values for BiACA derivatives.

To prove this hypothesis experimentally, we synthesized ACA dimers, *i.e.*, bisarylcyanoacetates (BiACAs), with a series of functional groups at the *para*-position as novel RMs (Schemes 1, S1 and S2†). In contrast to the complicated synthetic protocols for previously reported RMs such as DABBF, TASN, and DFSN,^48–51^ the BiACA structures provided significantly more accessible protocols to the desired electron-deficient RMs, allowing a systematic investigation of the substituent effect. All BiACAs were obtained without peroxidized products, which is indicative of low-lying singly occupied molecular orbital (SOMO) levels^52–54^ and slow C–C bond cleavage under ambient conditions (*vide infra*). We conducted variable-temperature electron paramagnetic resonance (EPR) spectroscopic measurements to determine the bond dissociation ratio (BDR) of the central C–C bond of the RMs and their dissociation constant (*K*_d_) at 100 °C (Table S1†). In Fig. 2, the BDR values and the natural logarithms of *K*_d_ (ln *K*_d_) are plotted as a function of increasing RSE and *σ*_p_ values of the BiACAs. As expected, the BDR and ln *K*_d_ values decrease with increasing RSE and *σ*_p_ values. Specifically, BDR and ln *K*_d_ values of 158 × 10^−3^% and −16.7, respectively, were obtained for BiACA-1 (RSE: −145.34 kJ mol^−1^, *σ*_p_: −0.83), 12.1 × 10^−3^% and −21.6 for BiACA-2 (RSE: −133.63 kJ mol^−1^, *σ*_p_: −0.27), 1.37 × 10^−3^% and −26.3 for BiACA-3 (RSE: −126.69 kJ mol^−1^, *σ*_p_: −0.17), 0.41 × 10^−3^% and −27.3 for BiACA-4 (RSE: −122.17 kJ mol^−1^, *σ*_p_: 0.00), and 0.20 × 10^−3^% and −28.7 for BiACA-5 (RSE: −122.17 kJ mol^−1^, *σ*_p_: 0.50). Furthermore, BiACA-5 exhibited one of the highest levels of thermal tolerance among our previously reported RMs. These results indicate that the thermal tolerance of RMs can be correlated with the RSE values and, therefore, with the substituent effect associated with the *σ*_p_ value.

**Fig. 3:**
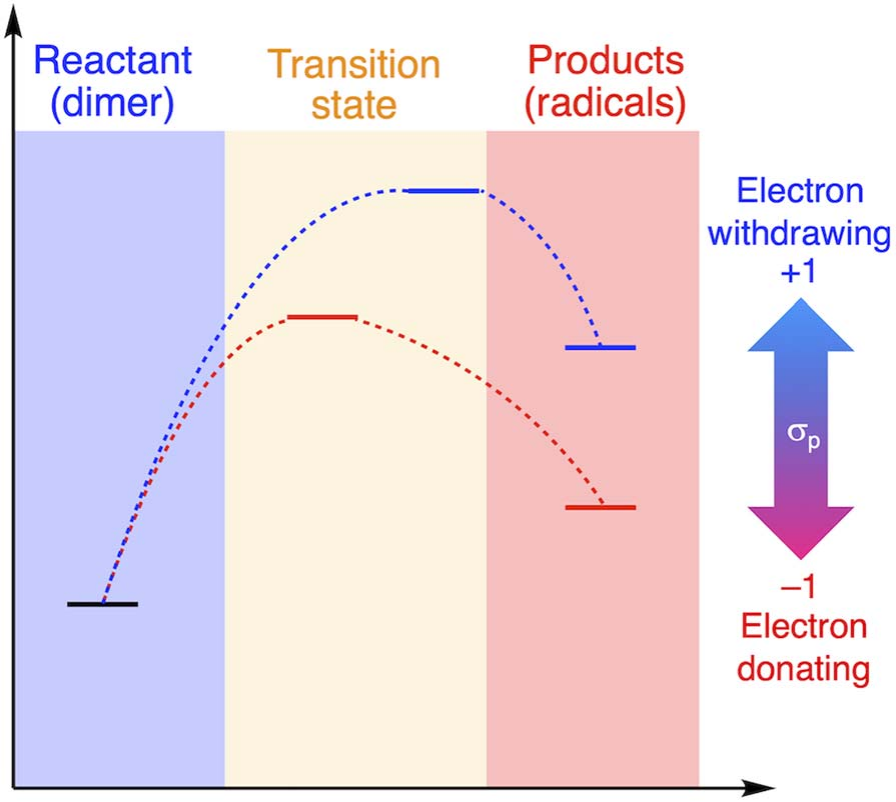
Schematic illustration of the change in the thermal tolerance according to the Hammond–Leffler postulate.

The relationship between RSE/thermodynamic stability and the thermal tolerance of RMs can be explained by the Hammond–Leffler postulate (Fig. 3), which describes the transition-state (TS) geometry in an elementary step related to reactants, intermediates, and products.^55,56^ According to this postulate, the energy barrier between reactants, *i.e.*, RMs, and products, *i.e.*, radicals generated from RMs, is amenable to the thermodynamic stability of the products. Thus, RMs that bear electron-withdrawing groups, which showed shallow RSE values and, in turn, low thermodynamic stability, exhibit a higher energy barrier for thermal dissociation into the corresponding radical products, indicating higher thermal tolerance. Although DFT is a powerful tool to evaluate the dissociation of the central C–C bond and the TS in RMs, the rotation of the central C–C bond, which leads to complicated rotamers, hinders the interpretability of the results. The present approach provides a simple method for the prediction of the thermal tolerance of RMs without complicated TS searches.

**Scheme 2:**
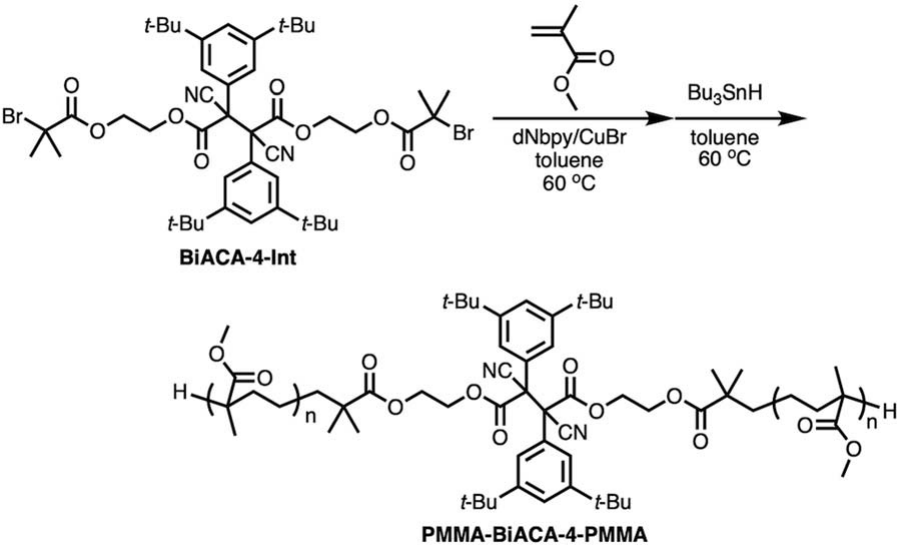
ATRP of methyl methacrylate initiated by bifunctional BiACA-4 initiator.

**Fig. 4:**
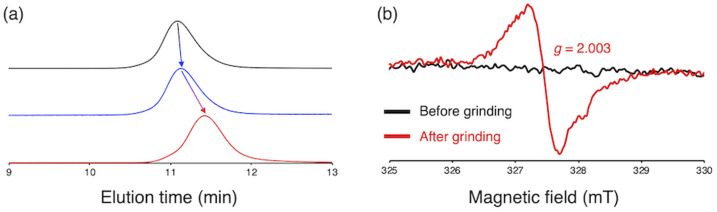
(a) GPC profiles of PMMA-BiACA-4-PMMA before (black line) and after conducting a heating test without BiACA-diol (blue line) and with BiACA-diol (red line); (b) solid-state ESR spectra of PMMA-BiACA-4-PMMA before and after grinding.

To investigate the mechanochemical reactivity of BiACAs, a poly(methyl methacrylate) (PMMA) derivative, PMMA-BiACA-4-PMMA, was synthesized *via* the atom transfer radical polymerization (ATRP) of methyl methacrylate using a bifunctional initiator consisting of the BiACA-4 skeleton (Scheme 2). The Br end groups were hydrogenated using tributyltin hydride to minimize possible side reactions.^57^ The *M*_n_ and *M*_w_/*M*_n_ values of PMMA-BiACA-4-PMMA were determined to be 24 kDa and 1.18, respectively, by gel-permeation chromatography (GPC) (Fig. 4a, black line). To verify whether the ATRP reaction was initiated and the PMMA chains grew bidirectionally from the central BiACA-4 moiety while maintaining the dynamic covalent properties of the latter, we conducted a heating test in the absence or presence of BiACA-4-diol as a scrambling unit (Scheme S4†). A comparison of the GPC profiles before and after heating PMMA-BiACA-4-PMMA in anisole at 130 °C for 90 minutes suggested that degradation did not occur (Fig. 4a, blue line), demonstrating the high thermal tolerance of the BiACA-4 unit to radical reactions below 130 °C. To investigate the mechanochemical reactivity of the polymer, we performed a grinding test on the solid sample by ball-milling at a frequency of 30 Hz for 60 minutes. A solid-state EPR spectrum recorded after grinding showed a clear signal with a *g*-value of 2.003, which was attributed to carbon-centered radicals. The BDR value (0.102% ± 0.006%) is 246 times higher than that obtained at 100 °C (0.41 × 10^−3^%), indicating that the mechanical stimulus (grinding) greatly enhances the C–C bond dissociation. These results indicate that the thermal tolerance of an RM can be increased without significantly deactivating its mechanochemical reactivity.

**Fig. 5:**
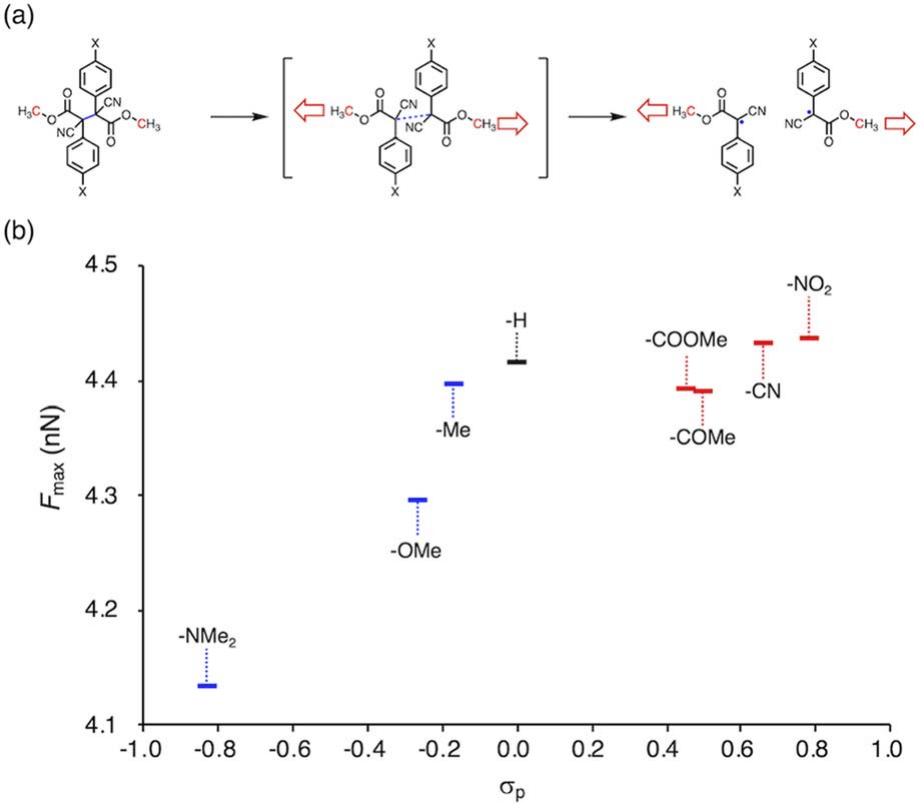
(a) Schematic illustration of the CoGEF calculations on the BiACA series (UB3LYP/6-31++G(d,p)). The red carbon atoms designate the anchor points, and the blue-colored bond is dissociated in the calculation. The red arrows indicate the direction of the force. (b) Plot of *F*_max_*vs. σ*_p_ for BiACA derivatives.

To gain more insight into the relationship between the thermal- and mechanochemical scission, we performed constrained-geometries-simulate-external-force (CoGEF) calculations, which allow theoretically evaluating the mechanochemical reactivity (Fig. 5a).^58–68^Fig. 5b shows a plot of the maximum force (*F*_max_) of BiACAs *vs. σ*_p_. The *F*_max_ value increases in the region *σ*_p_ < 0 and was almost saturated in the region *σ*_p_ > 0, similar to the case of the plots of RSE *vs. σ*_p_ (Fig. 1c and 2). These results indicate that the nature of the thermal and mechanical bond dissociation might be the same from the viewpoint of the thermodynamic stability of the products. The CoGEF calculations showed that as the central C–C bond energy increases, not only thermal scission but also mechanochemical scission is less likely to occur. Although this may seem contradictory at first glance, it might be due to the change in the potential energy surface under mechanical force, as pointed out by Martínez *et al.*^69^ This could also explain why the bond dissociation occurs even in the case of strong C–C bonds with shallow RSE and high *F*_max_ values. These results suggest that controlling the thermal tolerance of RMs having mechanochemical reactivity can be achieved by tuning the substituent effect, which opens new avenues for the design of RMs by controlling the dissociation bond energy of single bonds.

**Fig. 6:**
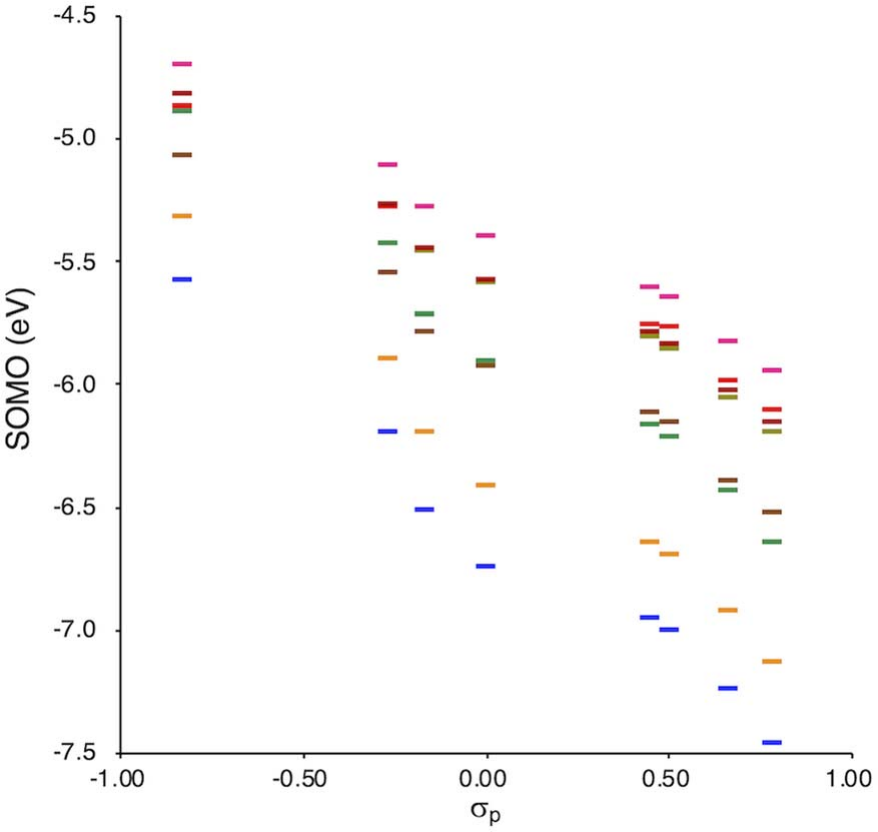
Calculated SOMO levels (UB3LYP/6-31++G(d,p)) *vs. σ*_p_.

SOMO levels are often used to evaluate the reactivity of molecules; therefore, they can be considered as a good indicator for the reactivity of RMs toward oxygen molecules.^54^ In this study, the SOMO levels clearly show a negative correlation with the *σ*_p_ values within the series of radicals that share the same skeleton, reflecting the substituent effect at the *para*-position (Fig. 6). ADCMs, which are stable radicals under ambient conditions reported by Sakamaki and Winter,^34–44^ show deep SOMO levels. Interestingly, the ACAs presented here also exhibit deep SOMO levels. These results indicates that the presence of electron-withdrawing groups shallows/deepens the RSE/SOMO, provides the RMs with thermal tolerance and could enable their handling under ambient conditions without peroxidation.

## Conclusions

In conclusion, we have demonstrated a rational designing strategy of RMs with high thermal tolerance. According to the relationship between the RSE and *σ*_p_ of the corresponding radicals, a series of thermally resistant RMs sharing similar skeletons could be simply designed. The thermal tolerance increased with increasing electron-withdrawing nature of the functional groups. The veracity of the RSE–*σ*_p_ relationship was experimentally validated by preparing a series of RMs that bear bisarylcyanoacetate (BiACA) skeletons with different electron density. Furthermore, not only the thermal but also the oxygen tolerance could be related to the SOMO level, and the oxygen tolerance can thus be tuned according to this parameter. Our results provide a systematic guideline for the design of RMs with various functionalities while maintaining good thermal tolerance.

## Data availability

The data generated in this study are available in the main text or the ESI.† Crystallographic data in the ESI† have been deposited at the joint Cambridge Crystallographic Data Centre (BiACA-4, CCDC: 2207403). The NMR and EPR spectra and the results of DFT calculation and CoGEF studies are provided in the ESI.†

## Author contributions

H. S., K. M. and H. O. conceptualized this work. H. S. designed the experiments and carried out DFT calculations. Y. L. performed experiments include ESR measurement. All authors wrote the manuscript. Y. L. and H. S. prepared the ESI.† All authors contributed to the editing and revision of the manuscript and ESI.†

## Conflicts of interest

There are no conflicts to declare.

## Supplementary Material

SC-014-D3SC02991C-s001

SC-014-D3SC02991C-s002
