# Supplementary material for: A rational design strategy of radical-type mechanophores with thermal tolerance
Source: Chem Sci. 2023 Aug 3;14(33):8792–7. doi: 10.1039/d3sc02991c (PMC10445462; doi:10.1039/d3sc02991c)
Supplement: SC-014-D3SC02991C-s001 [file SC-014-D3SC02991C-s001.pdf]

Electronic Supplementary Material (ESI) for Chemical Science.
This journal is © The Royal Society of Chemistry 2023

A Rational Design Strategy of Radical-type Mechanophores with Thermal Tolerance

Yi Lu,[†] Hajime Sugita,[†,§] Koichiro Mikami,[*,§] Daisuke Aoki,[†] and Hideyuki Otsuka[*,†,‖]

[†]Department of Chemical Science and Engineering, Tokyo Institute of Technology, 2-12-1 Ookayama, Meguro-ku, Tokyo 152-8550, Japan

[§]Sagami Chemical Research Institute, 2743-1 Hayakawa, Ayase, Kanagawa 252-1193, Japan

[‖]Living Systems Materialogy (LiSM) Research Group, International Research Frontiers Initiative (IRFI), Tokyo Institute of Technology, 4259 Nagatsuta-cho, Midori-ku, Yokohama 226-8501, Japan

Table of Contents

1. General information ········· 1
2. Synthesis procedures ········· 2-9
3. NMR spectra ········· 10-21
4. EPR studies ········· 22-24
5. Mechanochemical reactivity and dynamic covalent property of PMMA-BiACA-PMMA ········· 25
6. Results of computational studies ········· 26-38
7. CoGEF studies ········· 39-49
8. Computational details ········· 50-80
9. References ········· 81

1. General information

All reagents and solvents were purchased from Sigma-Aldrich, Wako Pure Chemical Industries, Tokyo Chemical Industry, or Kanto Chemical, and used as received, unless otherwise noted. ^{1}H NMR spectra were obtained using a 500 MHz or a 400 MHz Bruker spectrometer. ^{13}C NMR spectra were obtained using a 400 MHz JNM-ECZ400S/L1 JEOL spectrometer or a 500 MHz Bruker spectrometer. Electron paramagnetic resonance (EPR) measurements were carried out on a JEOL JES-X320 X-band EPR spectrometer equipped with a JEOL DVT temperature controller. The spectra were measured using a microwave of 0.1 mW and a field modulation 0.2 mT with a time constant of 0.03 s. The sweep rate was 0.25 mT/s. In variable temperature measurements from 50 to 130 °C, the spectra were measured after waiting for 1 min at each temperature. The concentration of the radicals was determined by comparing the integration of the observed integral spectrum with a ca. 0.02 mM solution of 4-hydroxy-2,2,6,6-tetramethylpiperidin-1-oxyl (TEMPOL) in anisole or benzene under the identical experimental conditions. The Mn^{2+} signal was used as an auxiliary standard. The g value was calculated according to the following equation: $g = hn/\beta H$ where h is the Planck constant, n is the microwave frequency, β is the Bohr magneton, and H is the magnetic field. Grinding tests of **PMMA-BiACA-4-PMMA** were performed on a Retsch Mixer Mill MM 400 with a frequency of 30 Hz. Gel permeation chromatography (GPC) measurements were carried out at 40 °C on TOSOH HLC-8320 GPC system equipped with a guard column (TOSOH TSK guard column Super H-L), three columns (TOSOH TSK gel SuperH 6000, 4000, and 2500) and a differential refractive index detector. Tetrahydrofuran (THF) was used as the eluent at a flow rate of 0.6 mL/min. Polystyrene (PS) standards (M_n = 4430–3242000; M_w/M_n = 1.03–1.08) were used to calibrate the GPC system. Density functional theory (DFT) calculations were performed using Gaussian 16 program package.[1]

2. Synthesis procedures

2-1. Syntheses of ACAs

The syntheses of **ACA-4** and **ACA-1** were done according to the reported method of Hartwig *et al.* (method A),[2] and the syntheses of **ACA-5**, **ACA-3**, and **ACA-2** were done according to the reported method of Jiang *et al* (method B).[3] The characterization results of **ACA-5**, **ACA-3**, and **ACA-2** were the same as the reported ones.

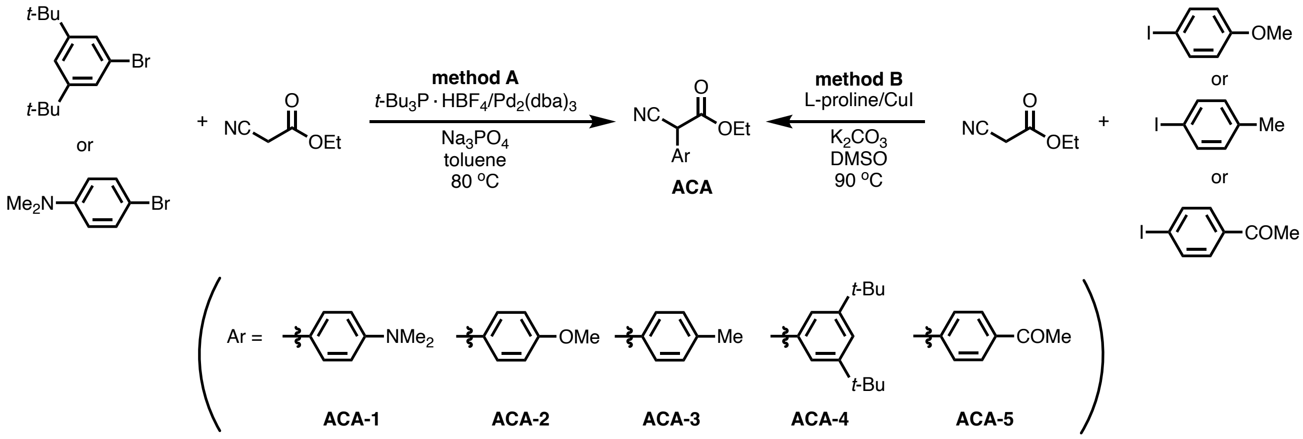

Scheme S1. Syntheses of ACAs.

ACA-4 (85%)

^{1}H-NMR (500 MHz, DMSO-*d*$_6$): δ/ppm 7.45 (t, *J* = 1.70 Hz, 1H), 7.26 (d, *J* = 1.70 Hz, 2H), 5.57 (s, 1H), 4.20 (q, *J* = 7.08 Hz, 2H), 1.29 (s, 18H), 1.19 (t, *J* = 7.08 Hz, 3H).

^{13}C-NMR (100 MHz, acetone-*d*$_6$): δ/ppm 165.61, 151.83, 130.50, 122.86, 122.31, 116.50, 62.55, 43.44, 34.78, 30.85, 13.47.

ESI-TOF-MS (*m/z*): [M+Na]$^+$ calcd. for $C_{19}H_{27}NNaO_2$: 324.1934; Found, 324.1933.

ACA-1 (29%)

^{1}H-NMR (500 MHz, acetone-*d*$_6$): δ/ppm 7.28 (d, *J* = 8.77 Hz, 2H), 6.77 (d, *J* = 8.88 Hz, 2H), 5.06 (s, 1H), 4.13-4.28 (m, 2H), 2.96 (s, 6H), 1.22 (t, *J* = 7.11 Hz, 3H).

^{13}C-NMR (100 MHz, acetone-*d*$_6$): δ/ppm 166.04, 151.01, 128.69, 118.02, 116.76, 112.55, 62.38, 42.31, 39.53, 13.40.

ESI-TOF-MS (*m/z*): [M+Na]$^+$ calcd. for $C_{13}H_{16}N_2NaO_2$: 255.1104; Found, 255.1097.

2-2. Synthesis of BiACAs

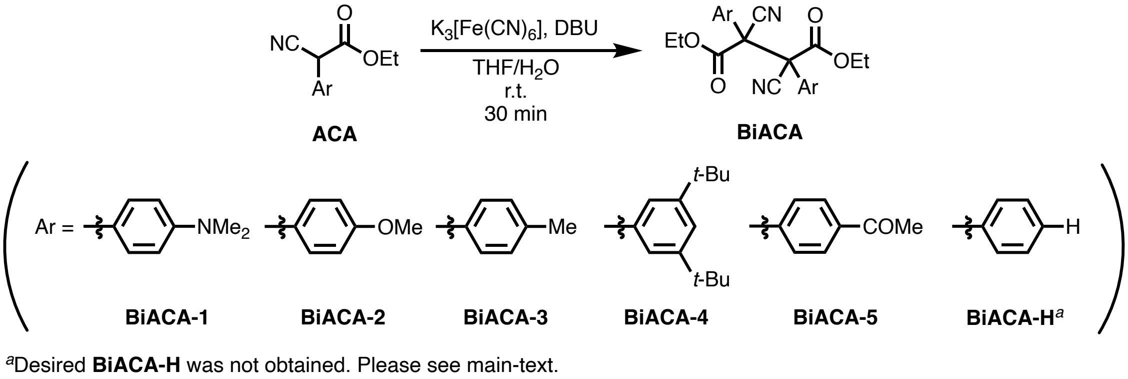

Scheme S2. Syntheses of **BiACA**s.

Syntheses of BiACAs

As a general method, ACA and $K_3[Fe(CN)_6]$ (1.5 eq.) were mixed in THF/H_2O (v/v = 4/1, $[ACA]_0$ = 0.05 M) combined solvent at room temperature. The mixture was degassed under reduced pressure and recharged with N_2 gas 3 times. To the resulting mixture was added DBU (4.0 eq.) dropwise. The mixture was stirred at room temperature for 30 minutes. Then, the mixture was transferred to a separation funnel. The organic layer was diluted with EtOAc, washed with saturated NaCl aqueous solution 3 times, collected, dried upon anhydrous $MgSO_4$, filtered, concentrated under vacuum, and purified with column chromatography (silica gel, EtOAc / hexane) to give **BiACA**. Single crystal X-ray analysis clearly showed the successful synthesis of **BiACA-4** (Fig. S1). Although we first attempted to synthesize **BiACA-H** (Scheme S2) rather than **BiACA-4** in which *t*-Bu groups were installed, unfortunately, the oxidative dimerization of **ACA-H** did not afford the desired product but several oligomeric products (Scheme S2).[4] This would be because high spin density appeared on the carbon at the 4-position of the aromatic unit (Fig. S35), and two *t*-Bu groups onto the 3,5-position of aromatic part of ACA were necessary for the kinetic stabilization.

BiACA-1 (72%)

^{1}H-NMR (500 MHz, acetone-d_6): δ/ppm 6.91 (d, J = 9.09 Hz, 2H), 6.68 (d, J = 9.09 Hz, 2H), 4.33–4.45 (m, 2H), 2.98 (s, 6H), 1.30 (t, J = 7.11 Hz, 3H).

^{13}C-NMR (100 MHz, acetone-d_6): δ/ppm 166.87, 151.29, 129.59, 116.00, 115.87, 111.19, 63.68, 60.34, 39.29, 13.28.

ESI-TOF-MS (*m/z*): $[M+Na]^+$ calcd. for $C_{26}H_{30}N_4NaO_4$: 485.2159; Found, 485.2156.

BiACA-2 (66%)

^{1}H-NMR (500 MHz, chloroform-d): δ/ppm 6.69–7.33 (m, 4H), 4.13–4.41 (m, 2H), 3.75 (s, 3H), 1.14–1.29 (m, 3H).

^{13}C-NMR (100 MHz, acetone-d_6): δ/ppm 166.34, 161.12, 130.17, 121.04, 115.60, 113.87, 64.16, 60.07, 55.00, 13.12.

ESI-TOF-MS (*m/z*): $[M+Na]^+$ calcd. for $C_{24}H_{24}N_2NaO_6$: 459.1527; Found, 459.1533.

BiACA-3 (66%)

^{1}H-NMR (500 MHz, acetone-*d*$_6$): δ/ppm 7.23 (d, *J* = 8.35 Hz, 2H), 6.97 (d, *J* = 8.35 Hz, 2H), 4.29–4.49 (m, 2H), 2.35–2.39 (m, 3H), 1.19–1.34 (m, 3H).

^{13}C-NMR (100 MHz, chloroform-*d*): δ/ppm 166.33, 164.50, 140.41, 140.34, 129.32, 129.27, 129.02, 128.66, 126.06, 116.30, 115.73, 64.39, 60.31, 21.20, 13.72.

ESI-TOF-MS (*m/z*): [M+Na]$^+$ calcd. for $C_{24}H_{24}N_2NaO_4$: 427.1628; Found, 427.1626.

BiACA-4 (93%)

Isomer 1:

^{1}H-NMR (400 MHz, acetone-*d*$_6$): δ/ppm 7.50 (t, *J* = 1.64 Hz, 1H), 7.25 (s, 2H), 4.12–4.25 (m, 2H), 1.19–1.30 (m, 21H).

^{13}C-NMR (100 MHz, acetone-*d*$_6$): δ/ppm 166.99, 151.95, 129.51, 134.54, 123.32, 116.79, 64.97, 61.64, 35.61, 31.48, 14.17.

ESI-TOF-MS (*m/z*): [M+Na]$^+$ calcd. for $C_{38}H_{52}N_2NaO_4$: 623.3827; Found, 623.3819.

Isomer 2:

^{1}H-NMR (400 MHz, acetone-*d*$_6$): δ/ppm 7.55 (t, *J* = 1.70 Hz, 1H), 7.07 (d, *J* = 1.68 Hz, 2H), 4.40–4.48 (m, 2H), 1.30 (t, *J* = 7.10 Hz, 3H), 1.23 (m, 18H).

^{13}C-NMR (100 MHz, acetone-*d*$_6$): δ/ppm 166.42, 152.68, 131.41, 123.77, 123.17, 117.30, 63.40, 44.34, 35.66, 31.79, 14.38.

ESI-TOF-MS (*m/z*): [M+Na]$^+$ calcd. for $C_{38}H_{52}N_2NaO_4$: 623.3827; Found, 623.3819.

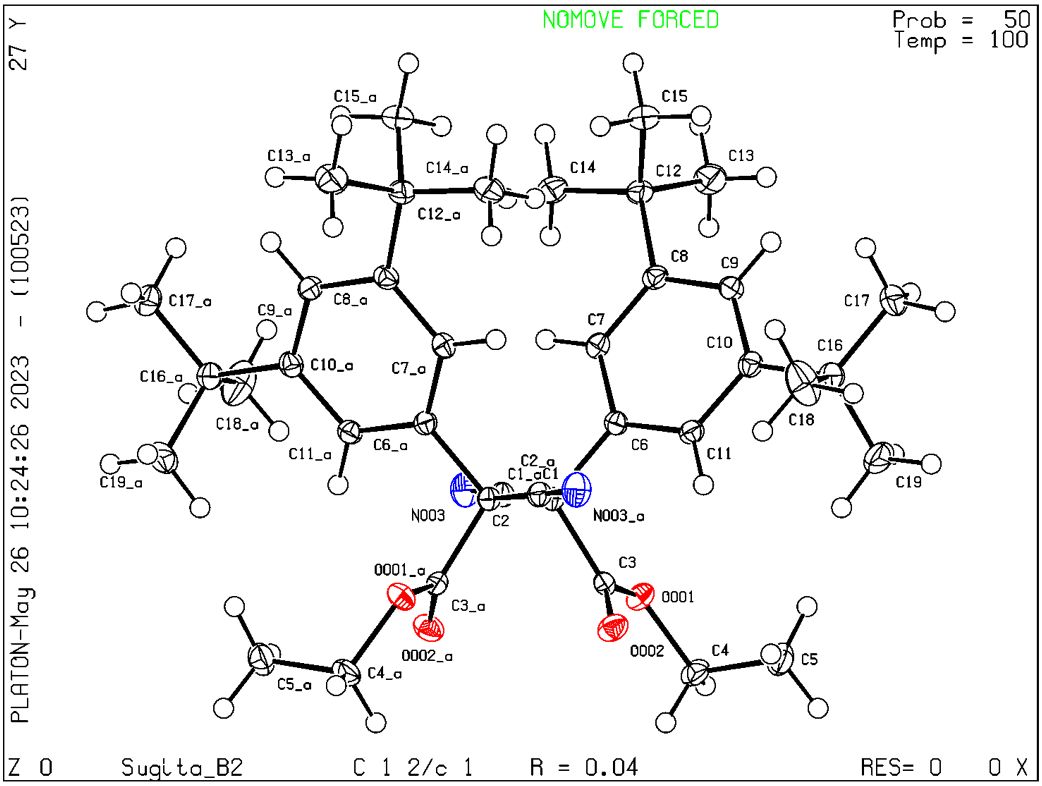

Fig. S1. Crystal structure of **BiACA-4**.

BiACA-5 (15%)

^{1}H-NMR (500 MHz, acetone-*d₆*): δ/ppm 7.98–8.06 (m, 2H), 7.19–7.56 (m, 2H), 4.34–4.59 (m, 2H), 2.63 (s, 3H), 1.23–1.35 (m, 3H).

^{13}C-NMR (100 MHz, acetone-d_6): δ/ppm 196.47, 165.49, 138.71, 133.25, 129.13, 128.49, 114.88, 64.92, 60.09, 26.06, 13.07.

ESI-TOF-MS (*m/z*): $[M+Na]^+$ calcd. for $C_{26}H_{24}N_2NaO_6$: 483.1527; Found, 483.1521.

2-3. Synthesis of **PMMA-BiACA-4-PMMA**

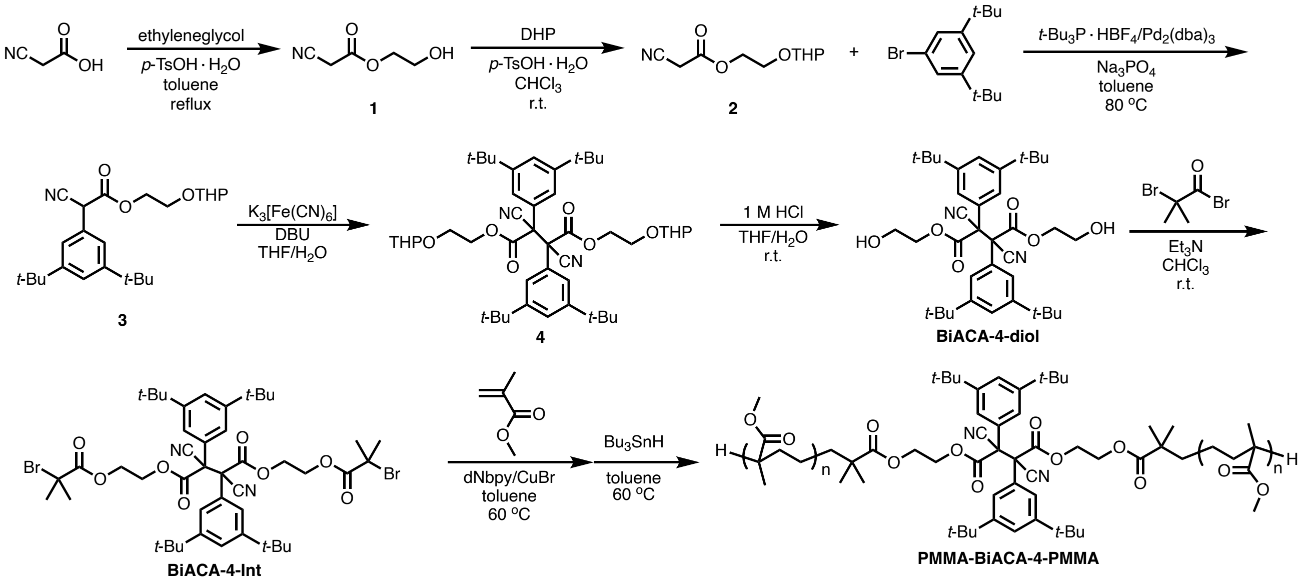

Scheme S3. Synthesis of **PMMA-BiACA-4-PMMA**.

2-Hydroxyethyl 2-cyanoacetate (1)

Cyanoacetic acid (5.0 g, 59 mmol), ethylene glycol (7.3 g, 6.6 mL, 120 mmol, 2.0 eq.), and *p*-toluenesulfonic acid monohydrate (0.3 g, 1.6 mmol, 2.7 mol%) were mixed in 20 mL dry toluene at room temperature. The mixture was heated to 130 °C in a round-bottom flask equipped with a Dean-Stark apparatus overnight. The mixture was cooled down to room temperature. 2-Hydroxyethyl 2-cyanoacetate (3.0 g, 24 mmol, 40%) was obtained by distillation under reduced pressure as a colorless oil-like liquid. The structure of the product was confirmed as reported.[5]

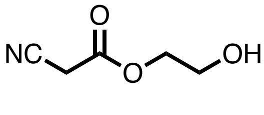

2-((Tetrahydro-2H-pyran-2-yl)oxy)ethyl 2-cyanoacetate (2)

1 (1.1 g, 8.3 mmol) and *p*-toluenesulfonic acid monohydrate (16 mg, 0.08 mmol, 1 mol%) were dissolved in 20 mL of dry $CHCl_3$ at 0 °C. To the solution was added 3,4-dihydro-2H-pyran (910 mg, 11 mmol, 1.3 eq.) dropwise. The mixture was allowed to warm up to room temperature and react overnight. $NaHCO_3$ was added to quench the acid and the mixture was transferred into a separation

funnel. The organic layer was washed with brine, collected, dried upon anhydrous $MgSO_4$, concentrated, and purified with column chromatography (EtOAc / hexane = 1 / 2) to afford a colorless oil-like liquid **2** (0.98 g, 4.6 mmol, 57%).

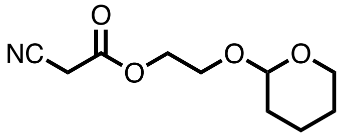

^{1}H-NMR (500 MHz, acetone-d_6): δ/ppm 4.64 (t, J = 3.35 Hz, 1H), 4.28–4.40 (m, 2H), 3.78–3.92 (m, 4H), 3.63–3.69 (m, 1H), 3.43–3.50 (m, 1H), 1.73–1.85 (m, 1H), 1.60–1.69 (s, 1H), 1.43–1.59 (m, 4H).
^{13}C-NMR (100 MHz, acetone-d_6): δ/ppm 164.05, 113.98, 98.40, 65.40, 64.53, 61.40, 30.38, 25.52, 24.05, 19.10.
ESI-TOF-MS (*m/z*): $[M+Na]^+$ calcd. for $C_{10}H_{15}NNaO_4$: 236.0893; Found, 236.0890.

2-((tetrahydro-2H-pyran-2-yl)oxy)ethyl 2-cyano-2-(3,5-di-tert-butylphenyl)acetate (3)

2 (980 mg, 4.6 mmol), 1-bromo-3,5-di-*tert*-butylbenzene (1.2 g, 4.6 mmol, 1.0 eq.), and sodium phosphate (2.3 g, 14 mmol, 3.0 eq.) were mixed in 30 mL dry toluene. The mixture was bubbled with N_2 for 30 minutes at room temperature. To the mixture was added tris(dibenzylideneacetone)dipalladium(0) (42 mg, 0.046 mmol, 1 mol%) and tri-tert-butylphosphonium tetrafluoroborate (53 mg, 0.18 mmol, 4 mol%). The mixture was bubbled with N_2 for another 30 minutes and then heated up to 80 °C overnight. After cooling down to room temperature, the mixture was transferred to a separation funnel. The organic layer was washed with brine, collected, dried upon anhydrous $MgSO_4$, concentrated in vacuum, and purified with column chromatography (silica gel, EtOAc / hexane = 1 / 10) to afford a colorless oil-like liquid **3** (1.3 g, 3.2 mmol, 72%).

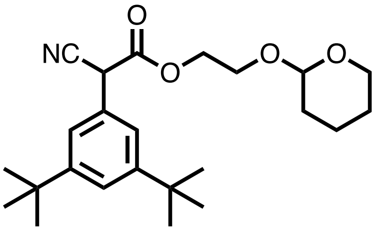

^{1}H-NMR (400 MHz, Acetone-d_6): δ/ppm 7.55 (t, J = 1.72 Hz, 1H), 7.39 (s, 2H), 5.28 (s, 1H), 4.55 (s, 1H), 4.36 (m, 2H), 3.84 (m, 1H), 3.72 (m, 1H), 3.62 (m, 1H), 3.41 (m, 1H), 1.71 (m, 1H), 1.38–1.62 (m, 5H), 1.34 (s, 18H).
^{13}C-NMR (100 MHz, acetone-d_6): δ/ppm 165.71, 151.83, 130.49, 123.00, 122.37, 116.48, 98.22, 65.90, 64.50, 61.20, 43.42, 34.77, 30.84, 30.30, 25.34, 18.98.
ESI-TOF-MS (*m/z*): $[M+Na]^+$ calcd. for $C_{24}H_{35}NNaO_4$: 424.2458; Found, 424.2469.

Bis(2-((tetrahydro-2H-pyran-2-yl)oxy)ethyl) 2,3-dicyano-2,3-bis(3,5-di-tert-butylphenyl) succinate (4)

3 (550 mg, 1.4 mmol) and $K_3[Fe(CN)_6]$ (580 mg, 1.8 mmol, 1.3 eq.) were mixed in THF / H_2O (12 mL / 3 mL) mixed solvent at room temperature under N_2 atmosphere. To the mixture was added DBU

(830 mg, 810 mL, 5.4 mmol, 4.0 eq.) dropwise and the resultant was stirred at room temperature for 30 minutes. Then, the mixture was transferred to a separation funnel. The organic layer was diluted with EtOAc, washed with saturated NaCl aqueous solution, concentrated, dried upon anhydrous $MgSO_4$, and purified with column chromatography (silica gel, EtOAc / hexane = 1 / 4) to give a white solid **4** (350 mg, 1.7 mmol, crude yield: 65%), which was directly used in the next step of the synthesis.

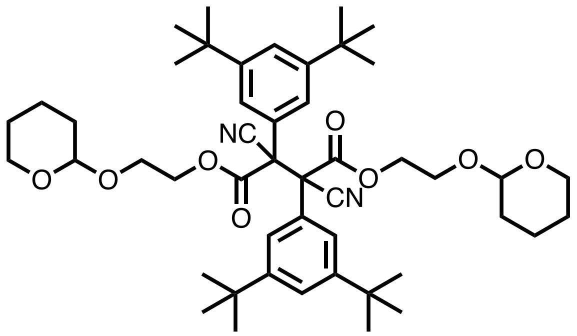

Bis(2-hydroxyethyl) 2,3-dicyano-2,3-bis(3,5-di-tert-butylphenyl)succinate (BiACA-4-diol)

4 (160 mg, 0.20 mmol) was dissolved in 10 mL of dry THF at room temperature. To the solution was added 10 mL 1 M HCl aqueous solution. The mixture was stirred at room temperature overnight. The acid was quenched with $NaHCO_3$ and then the organic layer was extracted with EtOAc, collected, dried upon $MgSO_4$, concentrated, and purified with column chromatography (silica gel, EtOAc / hexane = 1 / 1) to afford a white solid **5** (120 mg, 0.19 mmol, 92%). The **5** contained 2 isomers, which could be confirmed by NMR spectroscopy, mass spectroscopy, and X-ray crystallographic analysis.

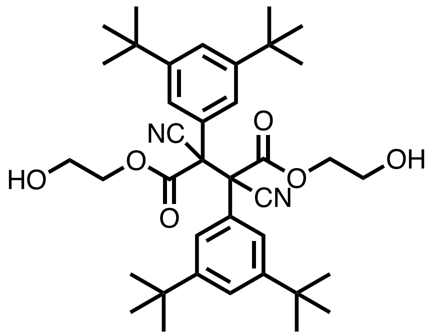

Isomer 1:

^{1}H-NMR (400 MHz, Acetone-*d*$_6$): δ/ppm 7.48 (t, *J* = 1.52 Hz, 1H), 7.21 (s, 2H), 4.29–4.55 (m, 2H), 3.99 (t, *J* = 5.54 Hz, 1H) 3.78 (q, *J* = 5.18 Hz, 2H), 1.23 (s, 18H).

^{13}C-NMR (100 MHz, Acetone-*d*$_6$): δ/ppm 166.03, 151.44, 129.71. 124.75, 124.26, 117.83, 70.19, 63.62, 60.13, 35.58, 31.51.

ESI-TOF-MS (*m/z*): $[M+Na]^+$ calcd. for $C_{38}H_{52}N_2NaO_6$: 655.3718; Found, 655.3716.

Isomer 2:

^{1}H-NMR (400 MHz, Acetone-*d*$_6$): δ/ppm 7.54 (t, *J* = 1.70 Hz, 1H), 7.10 (d, *J* = 1.64 Hz, 2H), 4.42–4.53 (m, 2H), 3.93 (t, *J* = 5.58 Hz, 1H) 3.81 (q, *J* = 5.20 Hz, 2H), 1.23 (s, 18H).

^{13}C-NMR (100 MHz, Acetone-d_6): δ/ppm 167.15, 151.86, 129.52, 123.57, 123.51, 116.82, 70.31, 61.98, 60.24, 35.60, 31.49.
ESI-TOF-MS (*m/z*): $[M+Na]^+$ calcd. for $C_{38}H_{52}N_2NaO_6$: 655.3718; Found, 655.3716.

BiACA-4-Int

BiACA-4-diol (130 mg, 0.20 mmol, a mixture of isomers) and 2-bromoisobutyryl bromide (190 mg, 0.82 mmol, 4.0 eq.) were dissolved in 10 mL of dry dichloromethane. The mixture was cooled down to 0 °C. To the mixture was added triethylamine (120 mg, 170 mL, 1.2 mmol, 6.0 eq.) dropwise. The mixture was allowed to warm up to room temperature and react overnight. The mixture was transferred to a separation funnel and the organic layer was extracted with EtOAc, washed with brine, collected, dried upon anhydrous $MgSO_4$, concentrated, and purified with column chromatography (silica gel, EtOAc / hexane = 1 / 8) to afford a yellowish solid **BiACA-4--Int** (160 mg, 0.17 mmol, 84%).

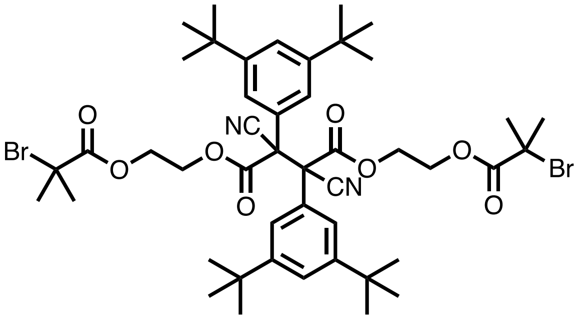

^{1}H-NMR (400 MHz, Acetone-d_6): δ/ppm 7.53 (s, 1H), 7.06 (s, 2H), 4.32-4.49 (m, 2H), 4.50–4.74 (m, 2H), 1.79 (s, 6H), 1.22 (s, 18H).
^{13}C-NMR (100 MHz, Acetone-d_6): δ/ppm 170.77, 166.11, 151.21, 150.82, 128.26, 123.84, 123.65, 122.32, 115.55, 65.55, 65.29, 62.82, 60.61, 55.87, 34.79, 30.69, 30.09.
ESI-TOF-MS (*m/z*): $[M+Na]^+$ calcd. for $C_{46}H_{62}Br_2N_2NaO_8$: 951.2765; Found, 951.2765.

Synthesis of PMMA-BiACA-4-PMMA (Scheme 2 (in main text))

BiACA-4-Int (64 mg, 0.068 mmol) and methyl methacrylate (4.1 g, 4.4 mL, 41 mmol, 600 eq.) were mixed in 7 mL of toluene at room temperature. The mixture was bubbled with N_2 for 30 minutes. To the mixture was added copper (I) bromide (25 mg, 0.17 mmol, 2.5 eq.) and 4,4′-Dinonyl-2,2′-dipyridyl (70 mg, 0.17 mmol, 2.5 eq.). The mixture was bubbled with N_2 for another 30 minutes. The mixture was heated to 60 °C for 2 hours. Then, to the mixture was added tributyltin hydride (240 mg, 41 mmol, 12 eq.), and the resulting mixture was heated for another 30 min. The mixture was cooled down to room temperature and quenched by adding $CHCl_3$. The copper complex was removed by passing the mixture through an active neutral alumina column. The resulting solution was concentrated and poured into methanol. The solid was collected by filtration to afford a white solid of **PMMA-BiACA-4-PMMA** (1200 mg, 74%, M_n = 24 kDa, PDI = 1.18). On the other hand, the addition of an excess amount of **BiACA-4-diol** followed by heating the mixture for another 90 minutes at 130 °C resulted in the reduction of molecular weight by half with narrow M_w/M_n (M_n = 14 kDa and M_w/M_n =

1.20). This result supported **BiACA-4** unit in the polymer produced from bifunctional ATRP still has the dynamic covalent property of the central C–C bond (Scheme S4).

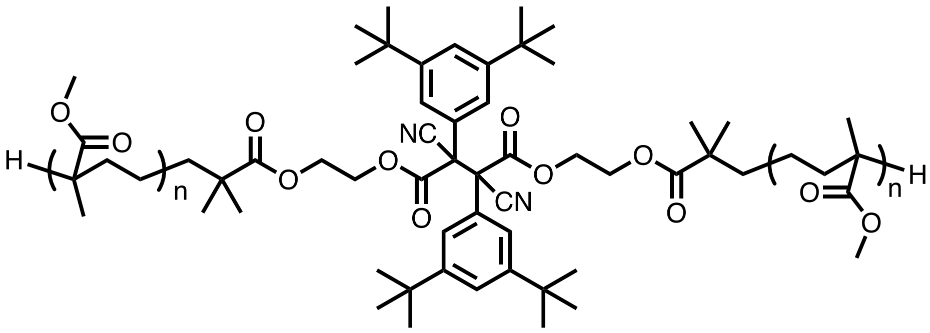

PMMA-BiACA-4PMMA

3. NMR spectra

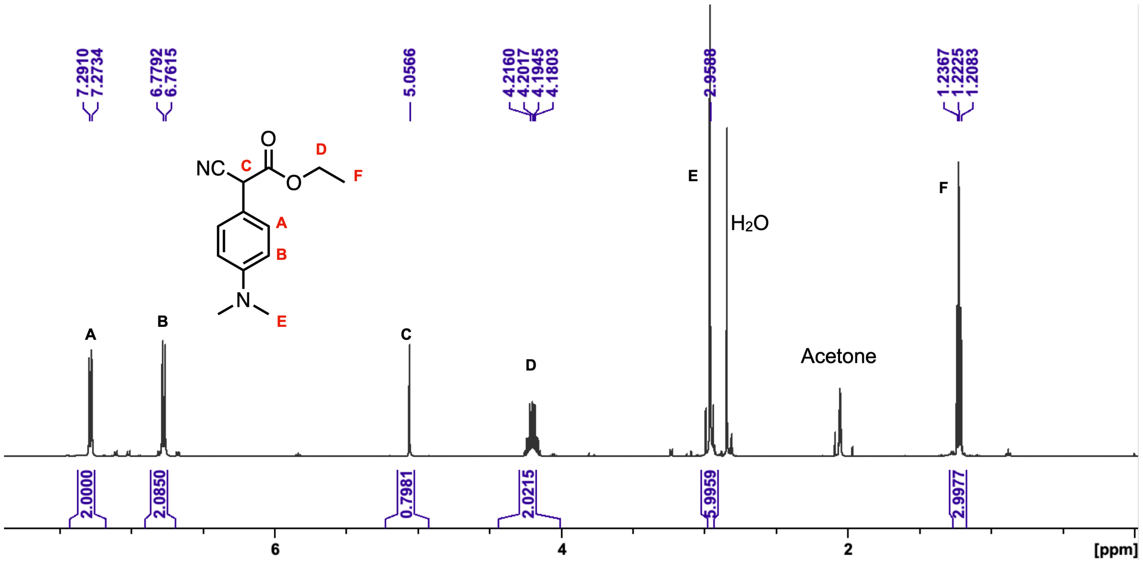

Fig. S2. ^{1}H NMR spectrum of **ACA-1** (500 MHz, 25 °C, acetone-d_6).

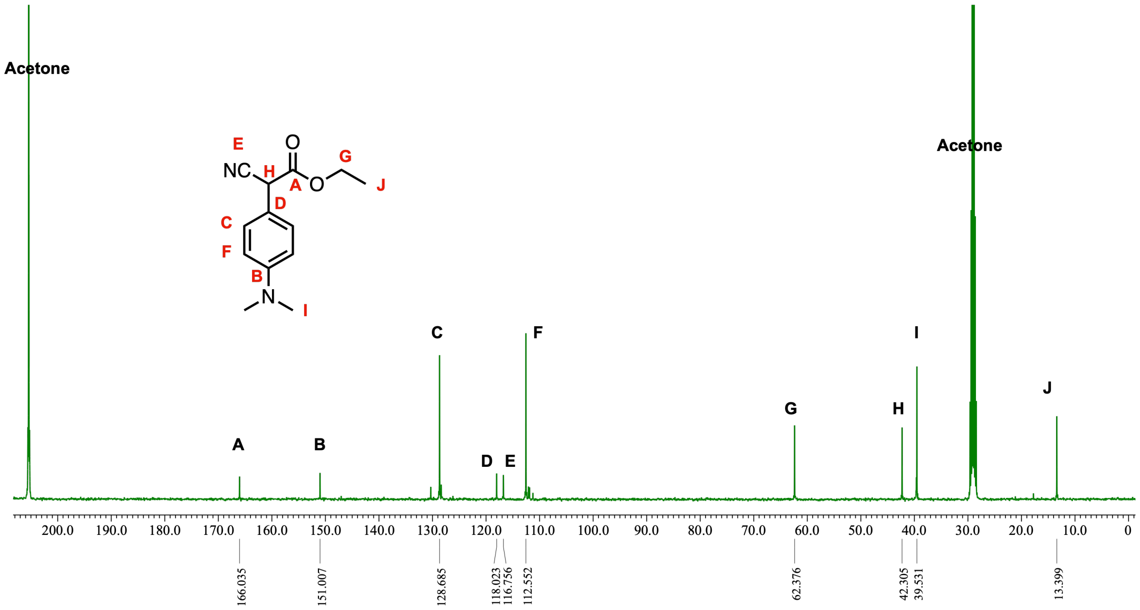

Fig. S3. ^{13}C NMR spectrum of **ACA-1** (100 MHz, 25 °C, acetone-d_6).

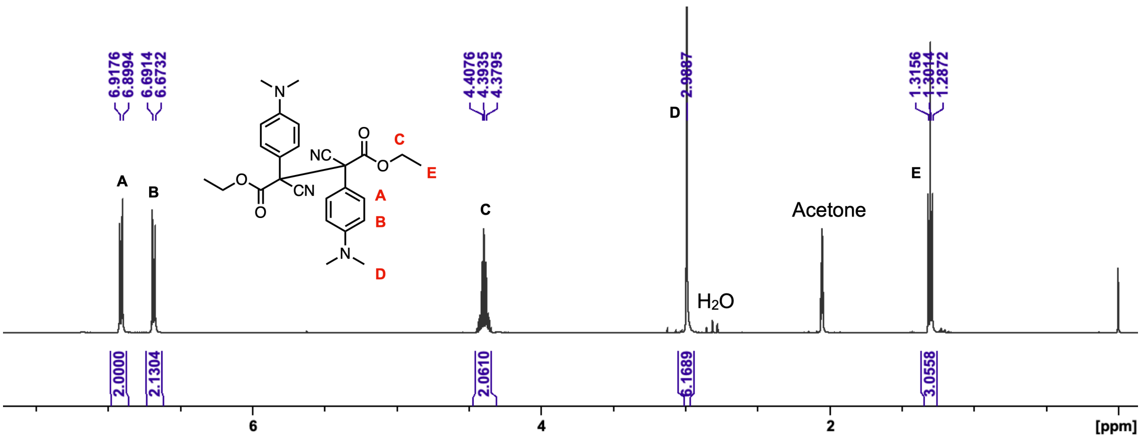

Fig. S4. ^{1}H NMR spectrum of **BiACA-1** (500 MHz, 25 °C, acetone-d_6).

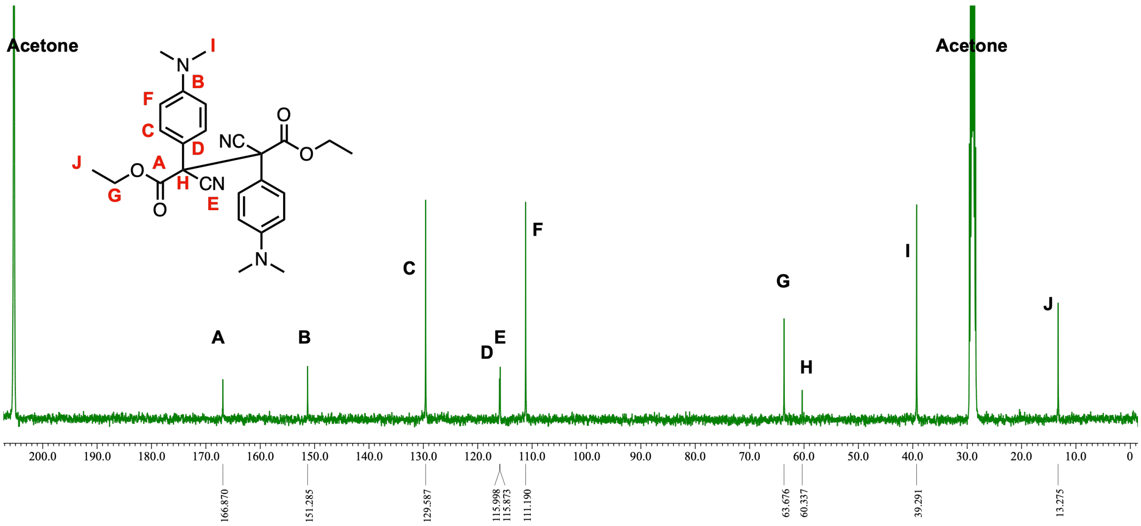

Fig. S5. ^{13}C NMR spectrum of **BiACA-1** (100 MHz, 25 °C, acetone-d_6).

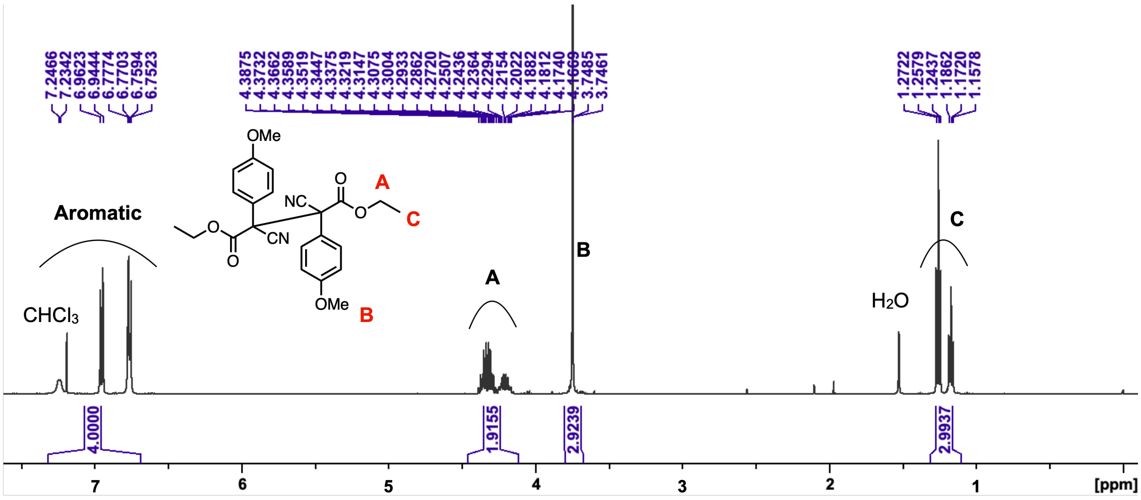

Fig. S6. ^{1}H NMR spectrum of **BiACA-2** (500 MHz, 25 °C, chloroform-d).

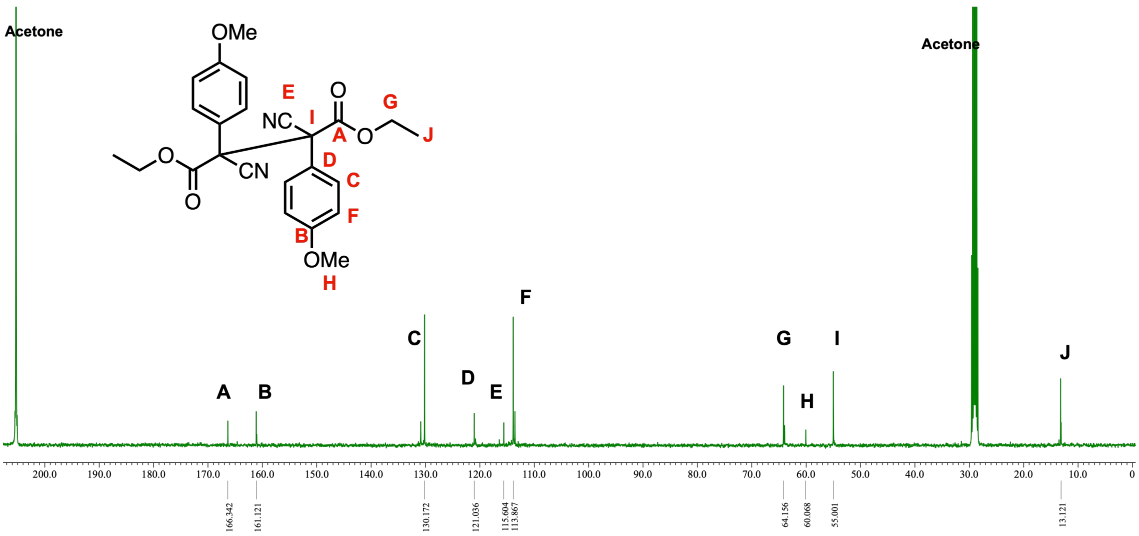

Fig. S7. ^{13}C NMR spectrum of **BiACA-2** (100 MHz, 25 °C, acetone-d_6).

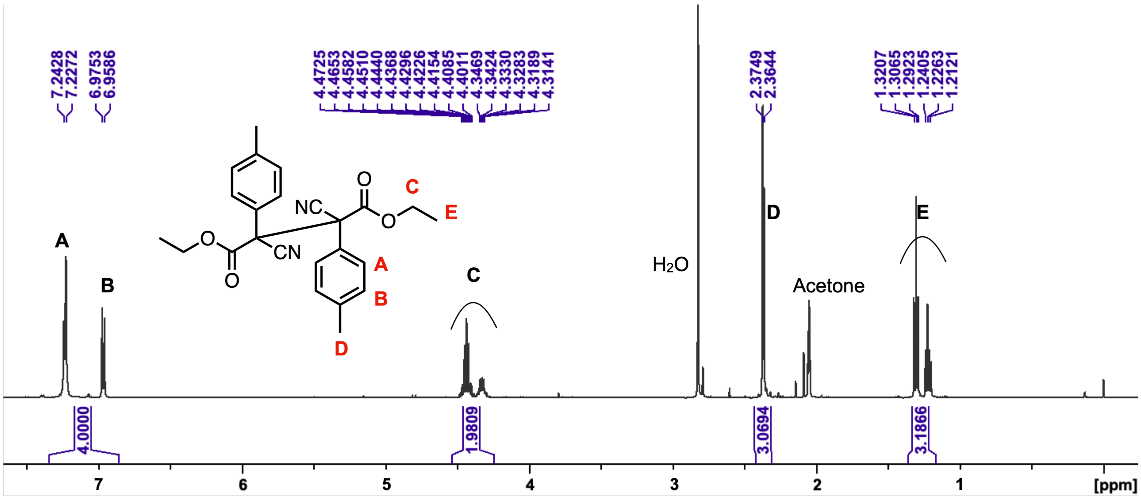

Fig. S8. ^{1}H NMR spectrum of **BiACA-3** (500 MHz, 25 °C, acetone-d_6).

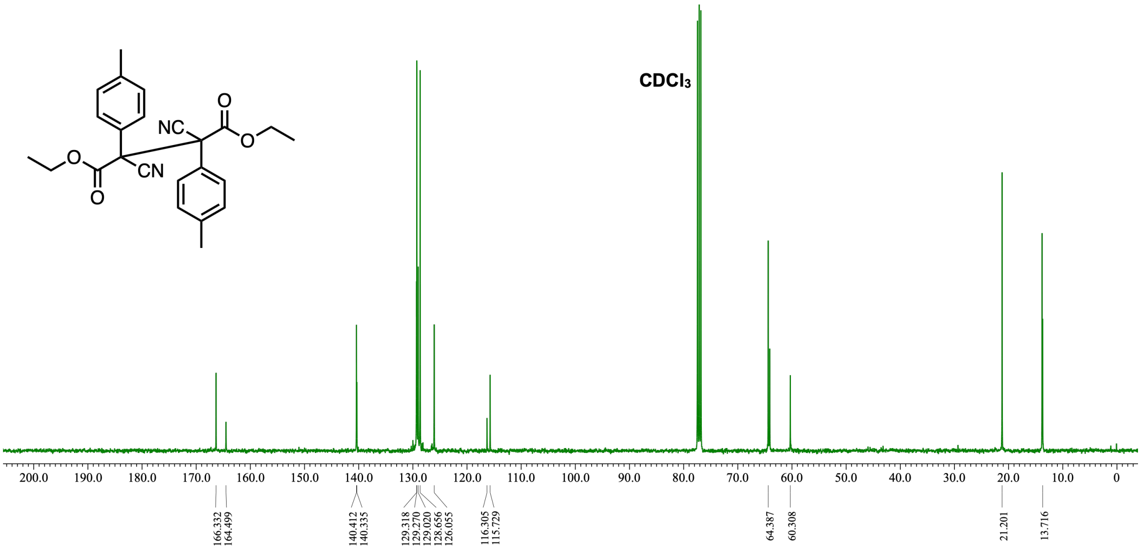

Fig. S9. ^{13}C NMR spectrum of **BiACA-3** (100 MHz, 25 °C, chloroform-d).

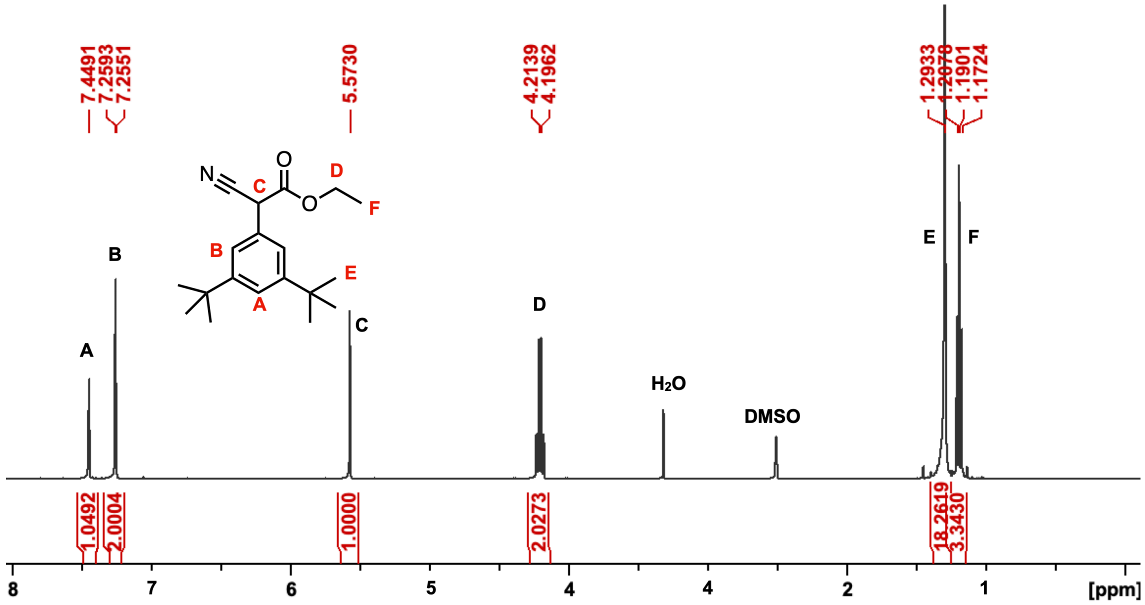

Fig. S10. ^{1}H NMR spectrum of **ACA-4** (500 MHz, 25 °C, DMSO-d_6).

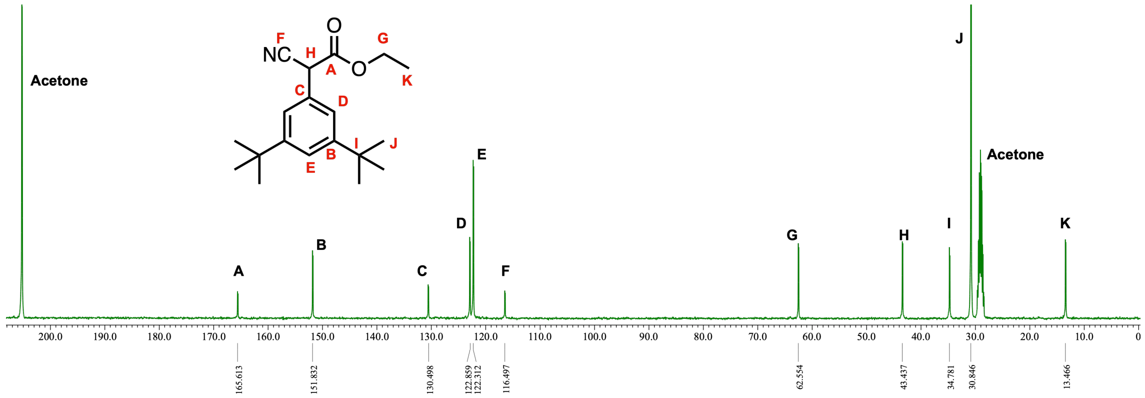

Fig. S11. ^{13}C NMR spectrum of **ACA-4** (100 MHz, 25 °C, acetone-d_6).

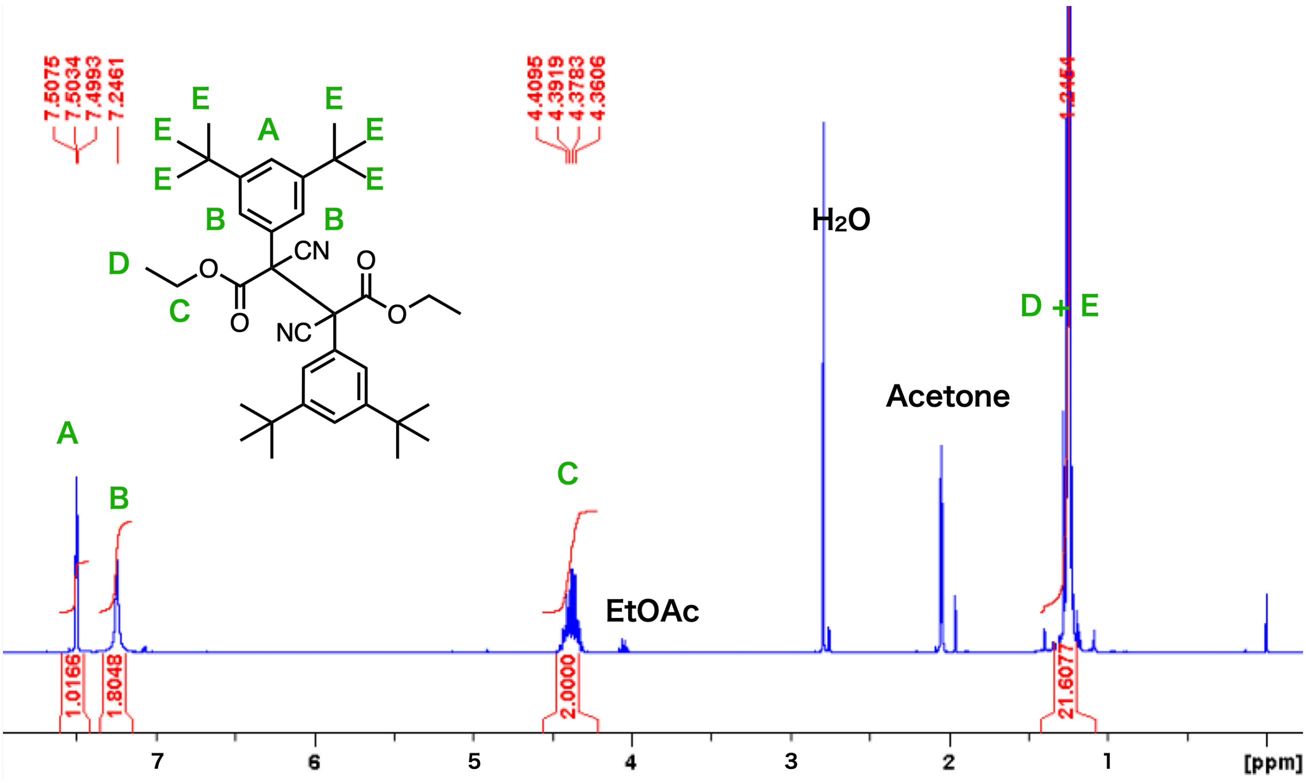

Fig. S12. ^{1}H NMR spectrum of **BiACA-4** (isomer 1, 400 MHz, 25 °C, acetone-d_6).

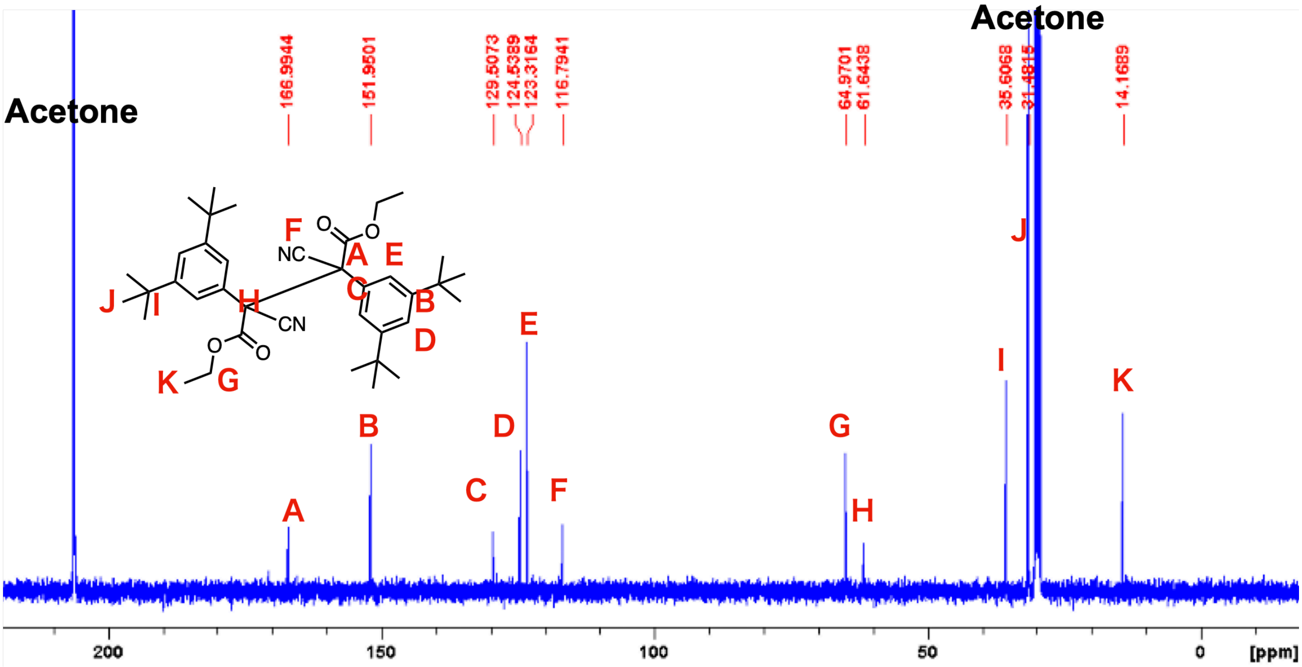

Fig. S13. ^{13}C NMR spectrum of **BiACA-4** (isomer 1, 100 MHz, 25 °C, acetone-d_6).

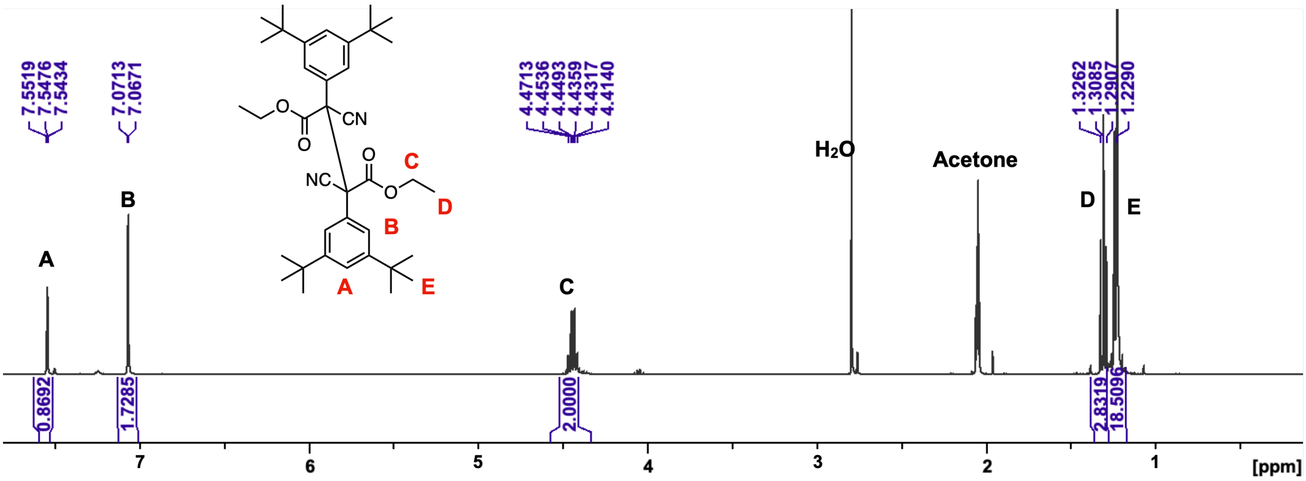

Fig. S14. ¹H NMR spectrum of **BiACA-4** (isomer 2, 400 MHz, 25 °C, acetone-d_6).

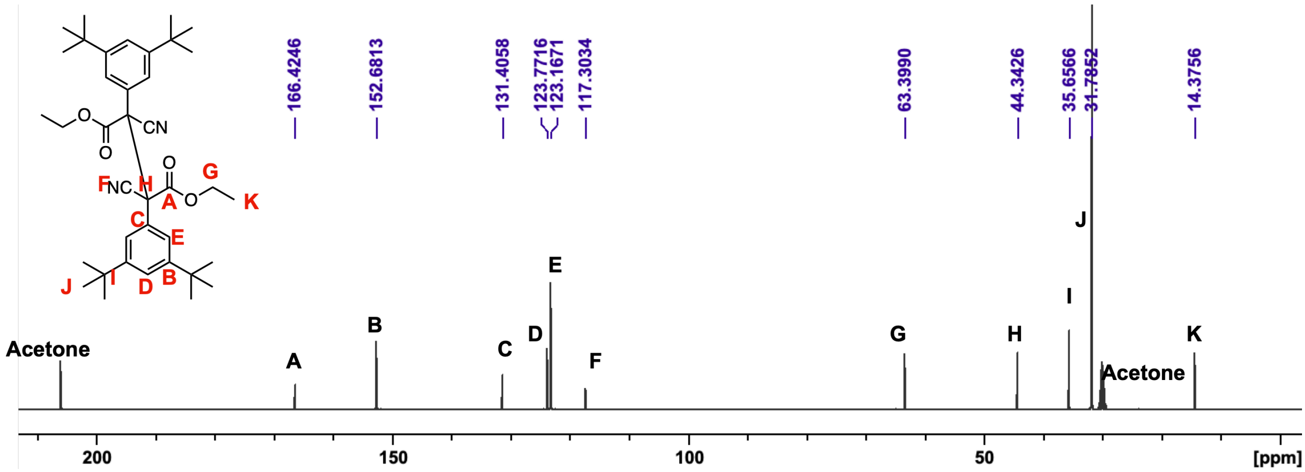

Fig. S15. ¹³C NMR spectrum of **BiACA-4** (isomer 2, 100 MHz, 25 °C, acetone-d_6).

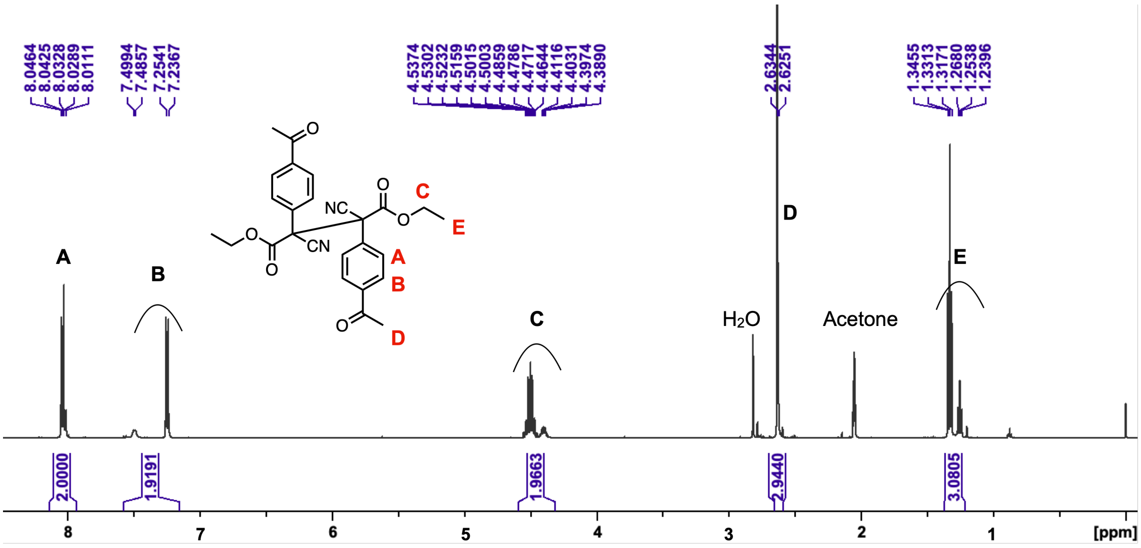

Fig. S16. ¹H NMR spectrum of **BiACA-5** (500 MHz, 25 °C, acetone-d_6).

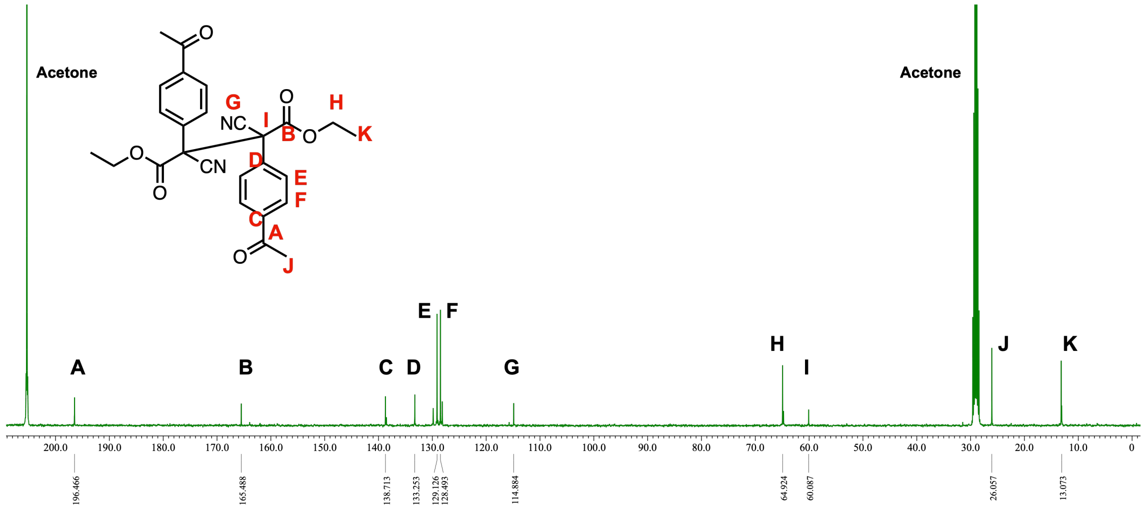

Fig. S17. ^{13}C NMR spectrum of **BiACA-5** (100 MHz, 25 °C, acetone-d_6).

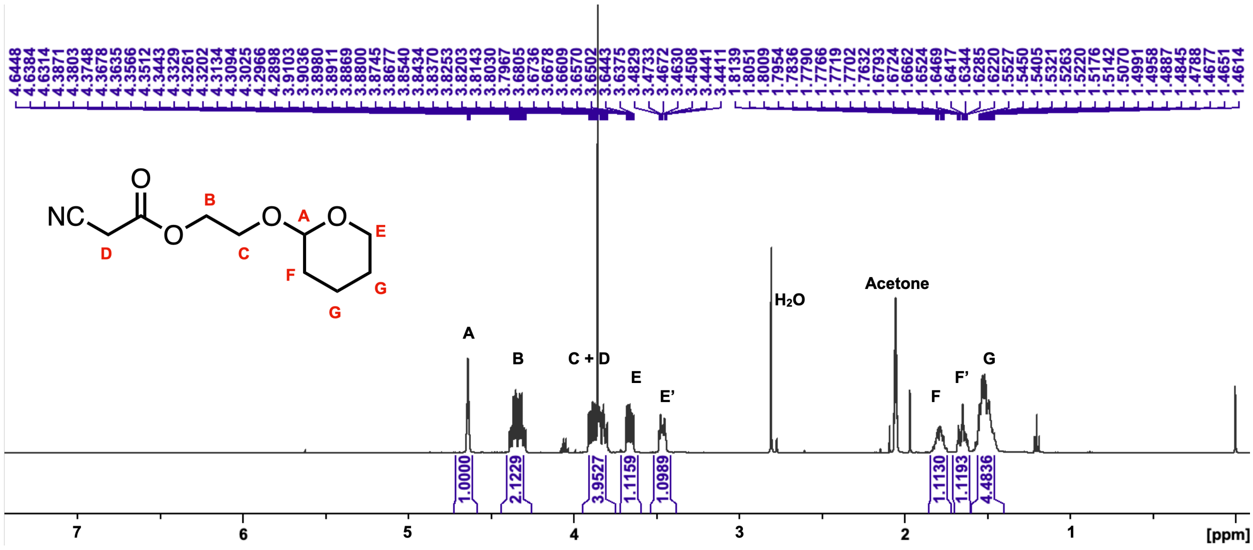

Fig. S18. ^{1}H NMR spectrum of **2** (25 °C, 400 MHz, acetone-d_6).

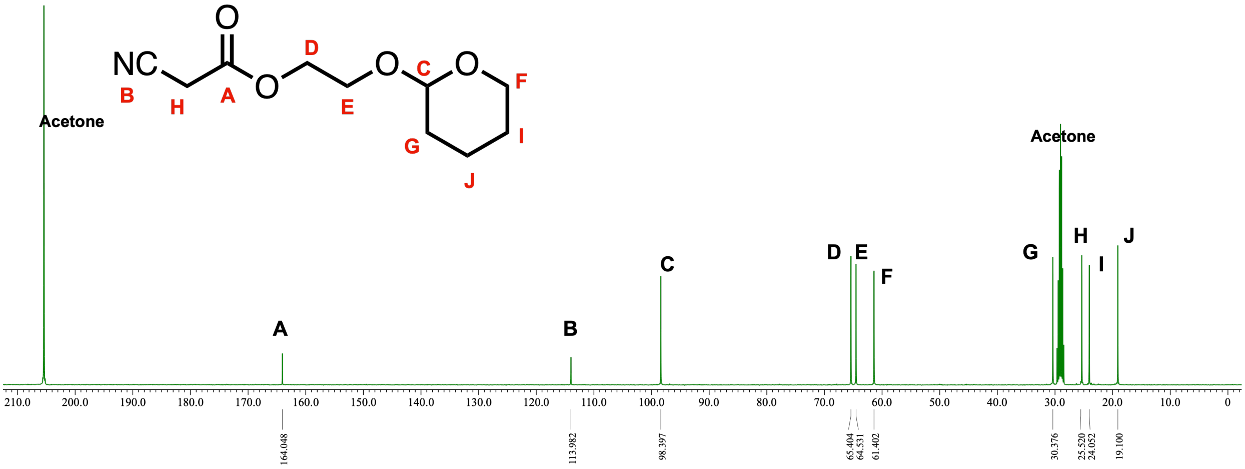

Fig. S19. ^{13}C NMR spectrum of **2** (25 °C, 100 MHz, acetone-d_6).

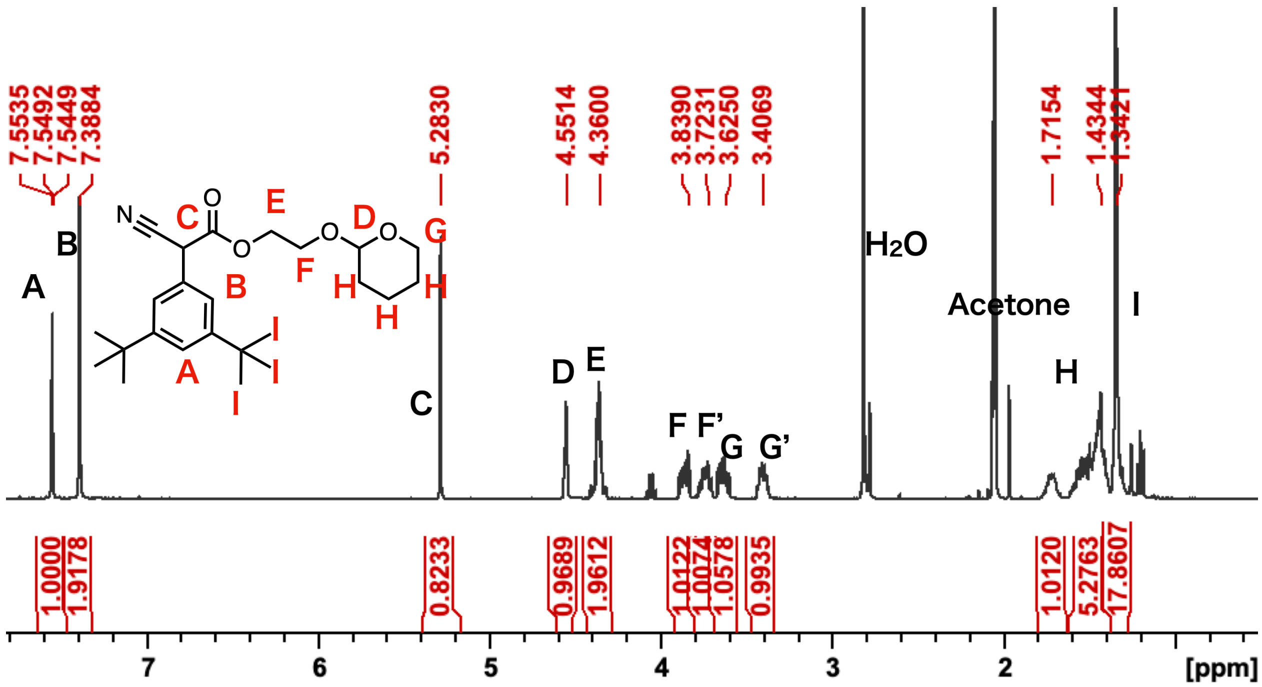

Fig. S20. ^{1}H NMR spectrum of **3** (25 °C, 400 MHz, acetone-d_6).

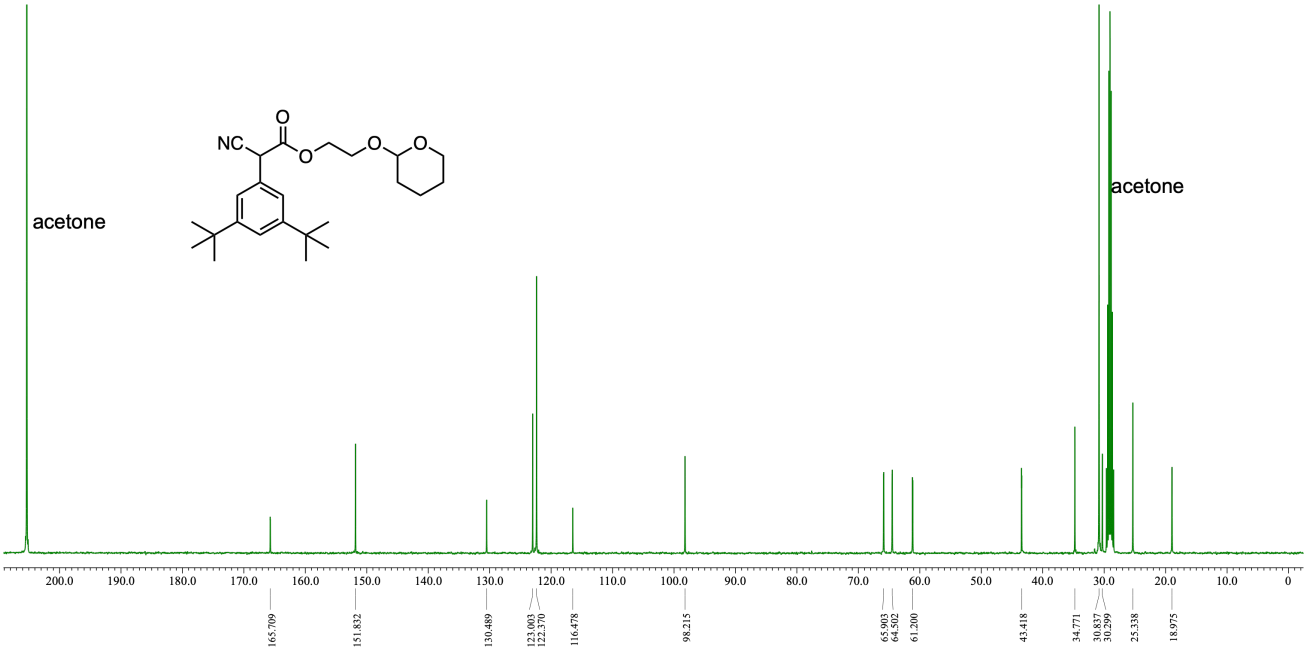

Fig. S21. ^{13}C NMR spectrum of **3** (25 °C, 100 MHz, acetone-d_6).

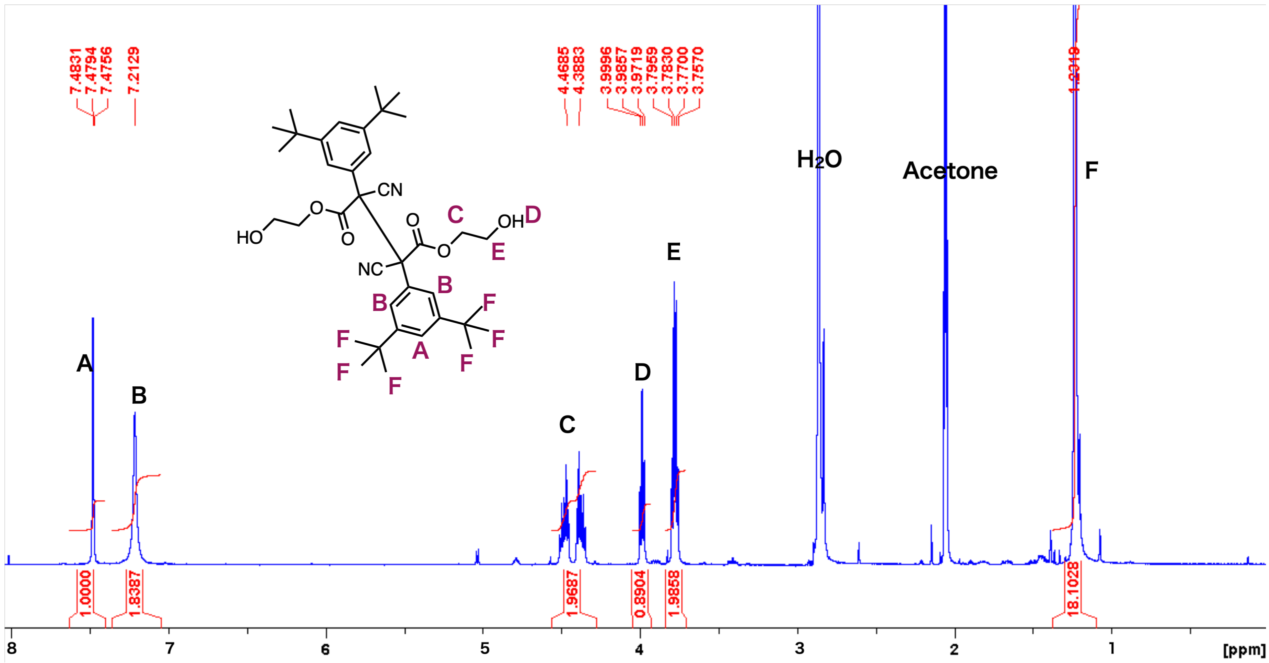

Fig. S22. ^{1}H NMR spectrum of **BiACA-4-diol** (isomer 1, 25 °C, 400 MHz, acetone-d_6).

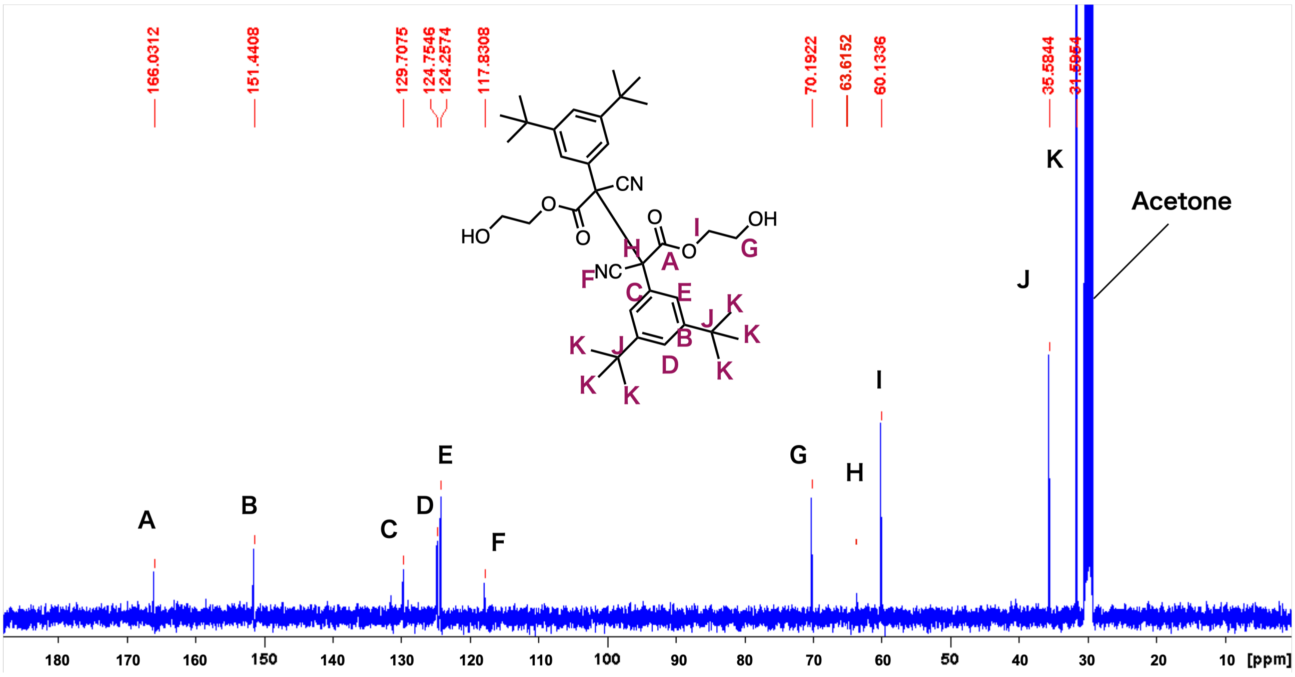

Fig. S23. ^{13}C NMR spectrum of **BiACA-4-diol** (isomer 1, 25 °C, 100 MHz, acetone-d_6).

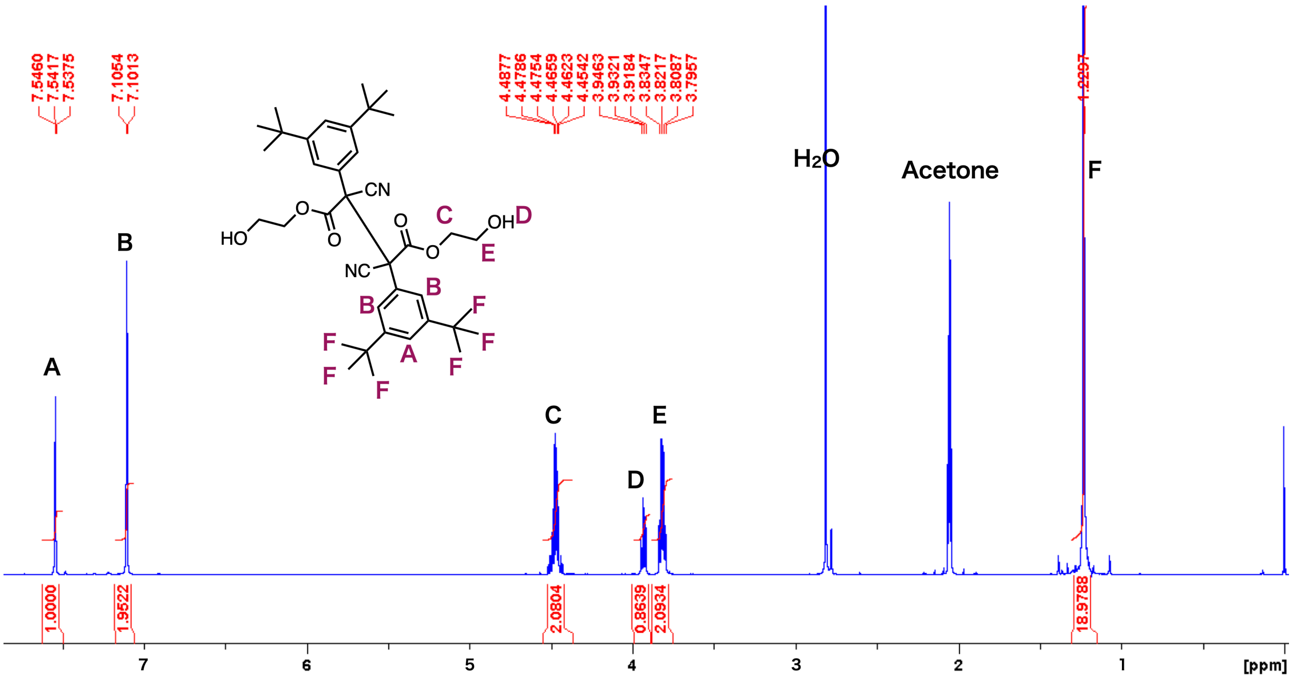

Fig. S24. ^{1}H NMR spectrum of **BiACA-4-diol** (isomer 2, 25 °C, 400 MHz, acetone-d_6).

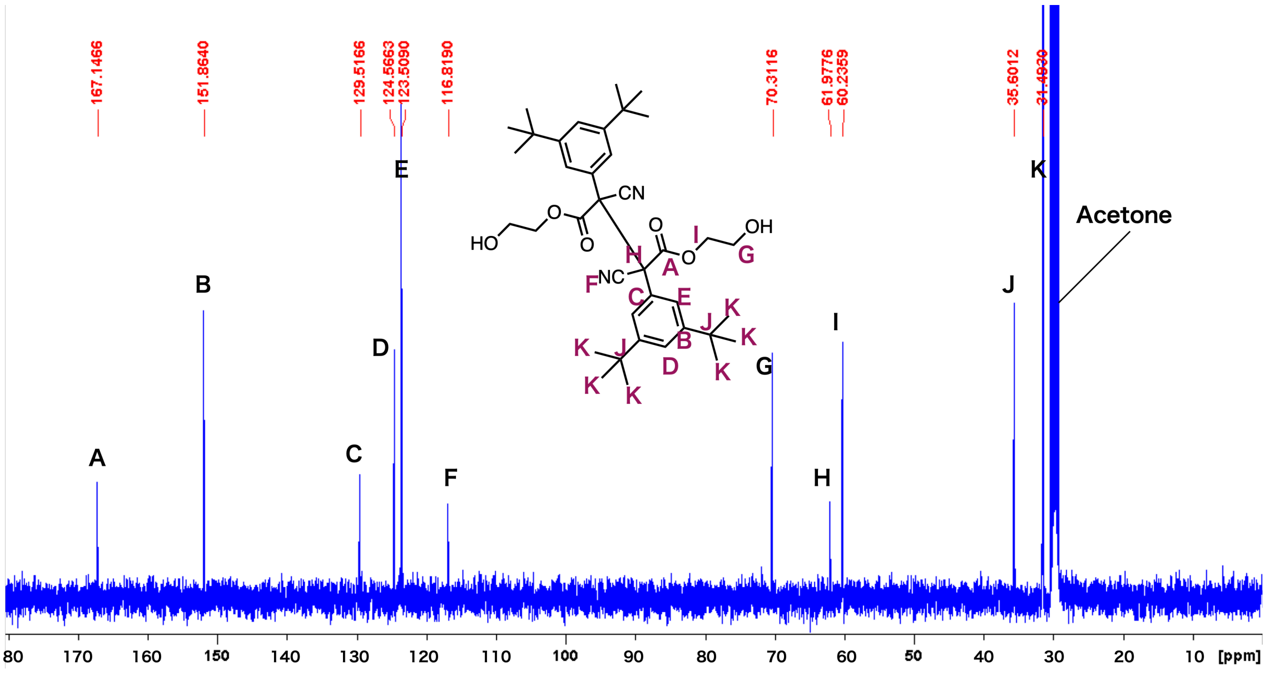

Fig. S25. ^{13}C NMR spectrum of **BiACA-4-diol** (isomer 2, 25 °C, 100 MHz, acetone-d_6).

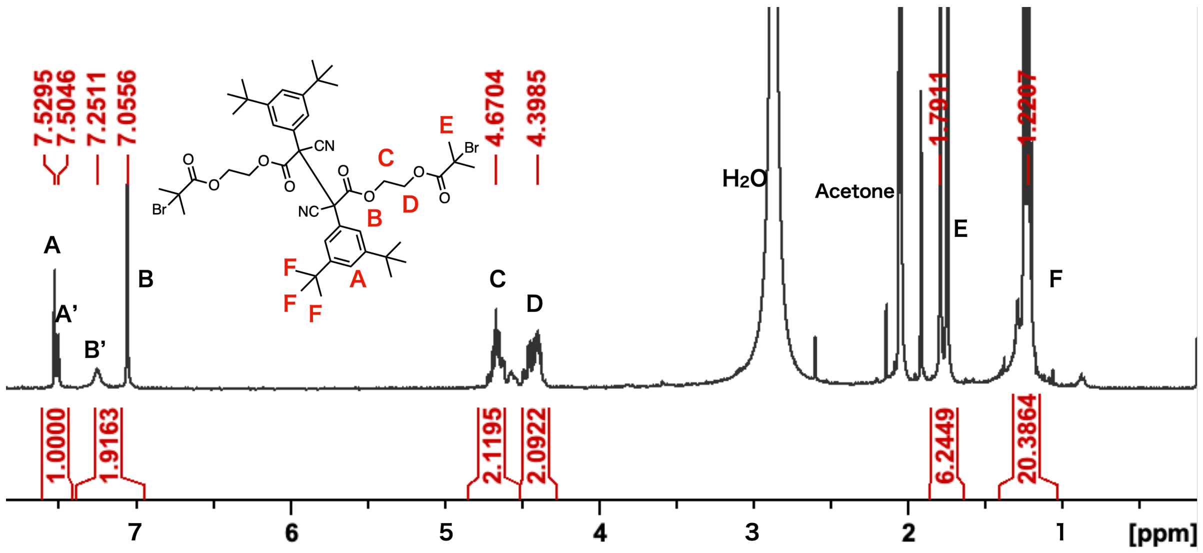

Fig. S26. ^{1}H NMR spectrum of **BiACA-4-Int** (25 °C, 400 MHz, acetone-d_6).

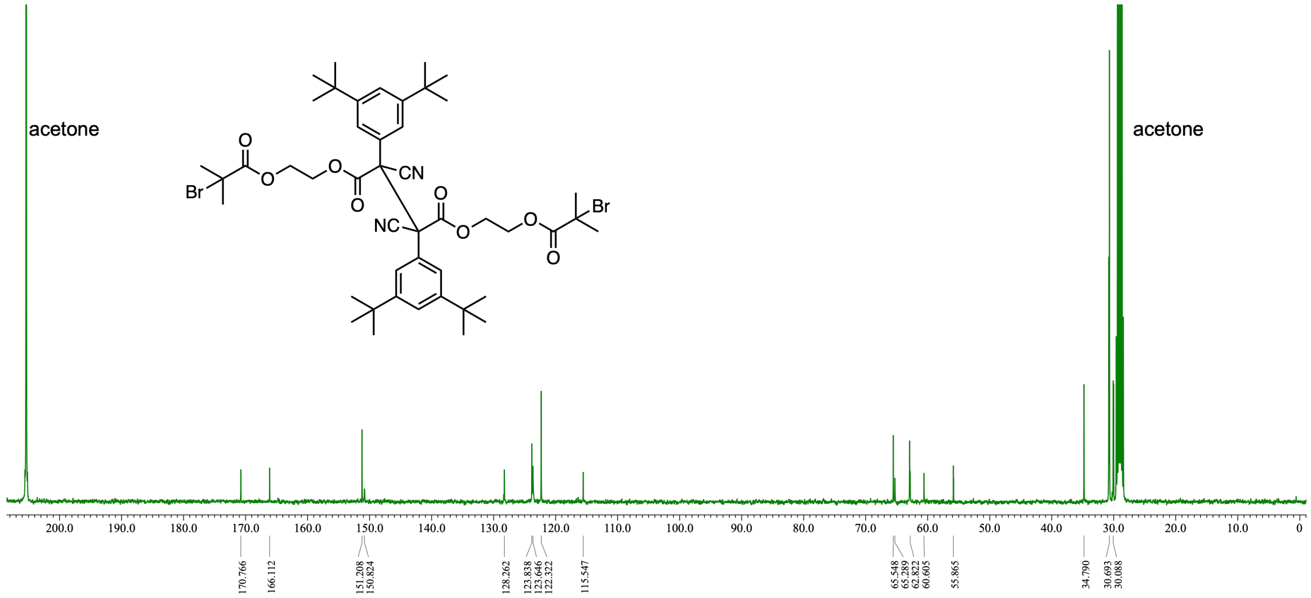

Fig. S27. ^{13}C NMR spectrum of **BiACA-4-Int** (25 °C, 100 MHz, acetone-d_6).

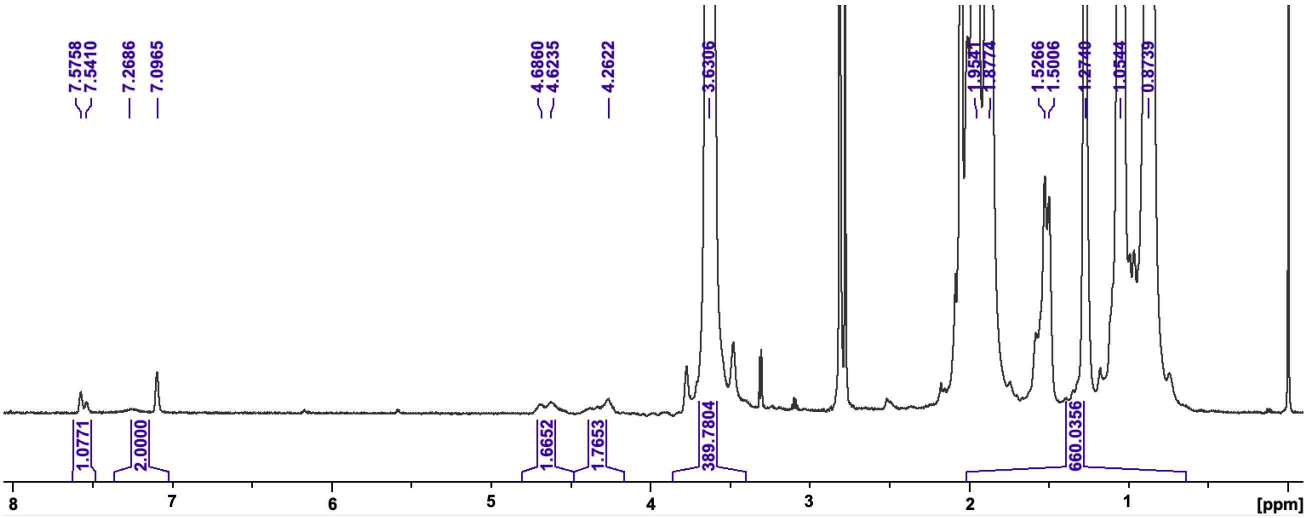

Fig. S28. ^{1}H NMR spectrum of **PMMA-BiACA-4-PMMA** (25 °C, 400 MHz, acetone-d_6).

4. EPR studies

5 mM anisole solutions of σ-dimers (20 mM for **BiACA-4** and **BiACA-5**, for better sensitivities) were charged in a 3 mm glass capillary with more than 43.5 mm height (effective measuring range), which was then sealed after freeze-thaw cycles. In all cases, the *g*-values of radical signals were determined as 2.003, suggesting the signals observed were carbon-centered radicals. The dissociation constants K_d were calculated, of which the natural logarithms ln K_d were plotted against $1/T$, where T was temperature in kelvin.

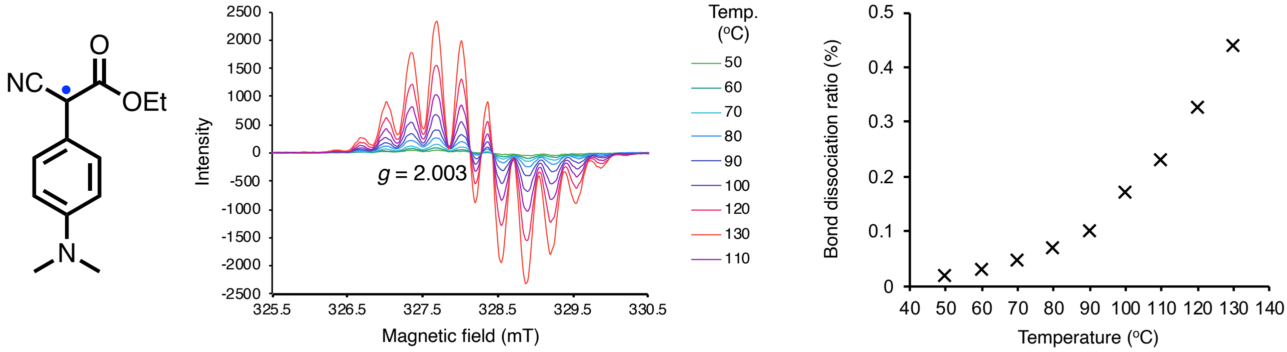

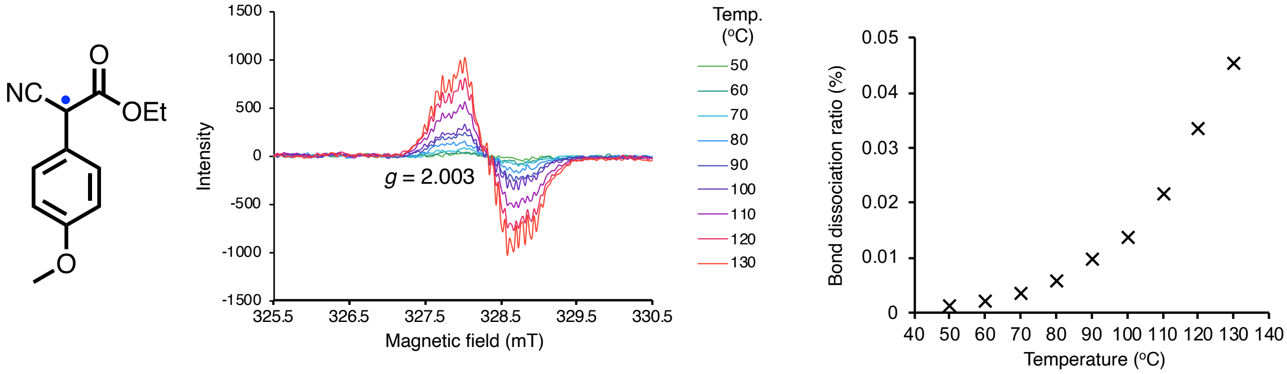

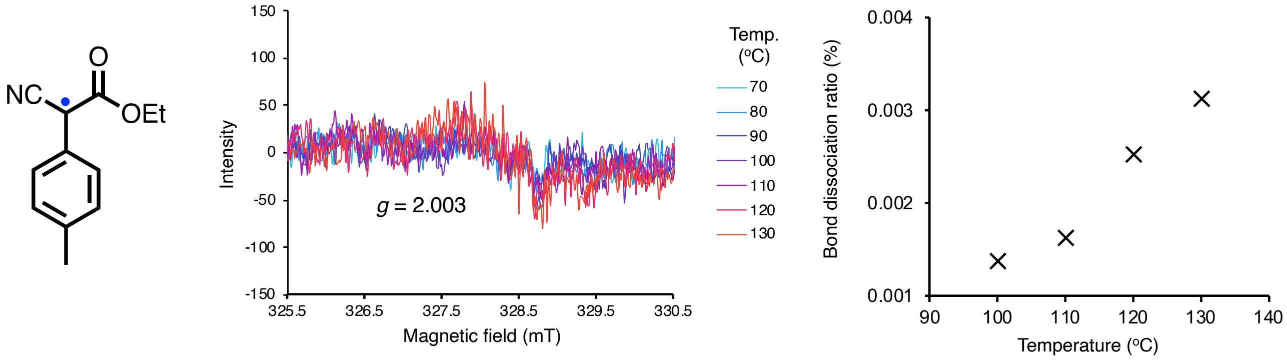

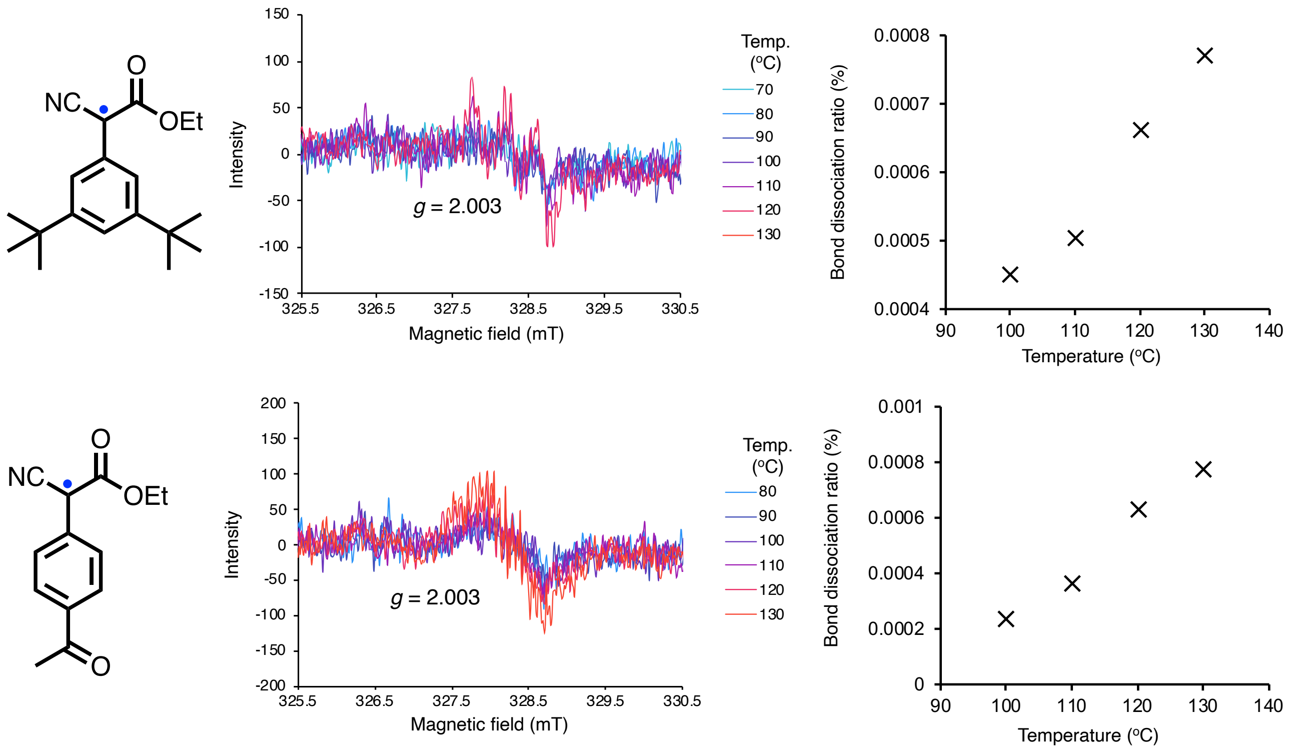

Fig. S29. VT-EPR measurement of **BiACAs** with bond dissociation ratio.

Correlation between computational values of RSE and experimental values of ln K_d.

The natural logarithms of K_d (ln K_d) at 100 °C in anisole of each compound were recorded with the computational results of RSE and *a*-SOMO levels in Table S1.

Table S1. Computational results of RSE, and SOMO energy, and experimental facts of ln K_d (100 °C, anisole)

Carbon-centered radicals	RSE (kJ / mol)	α-SOMO (eV)	ln K_d @100°C
**BiACA-1**	–145.34	–5.31	–16.68 ± 0.05
**BiACA-2**	–133.63	–5.89	–21.58 ± 0.03
**BiACA-3**	–126.69	–6.19	–26.20 ± 0.07
**BiACA-4**	–122.85	–6.20	–27.16 ± 0.09
**BiACA-5**	–122.17	–6.69	–28.42 ± 0.14

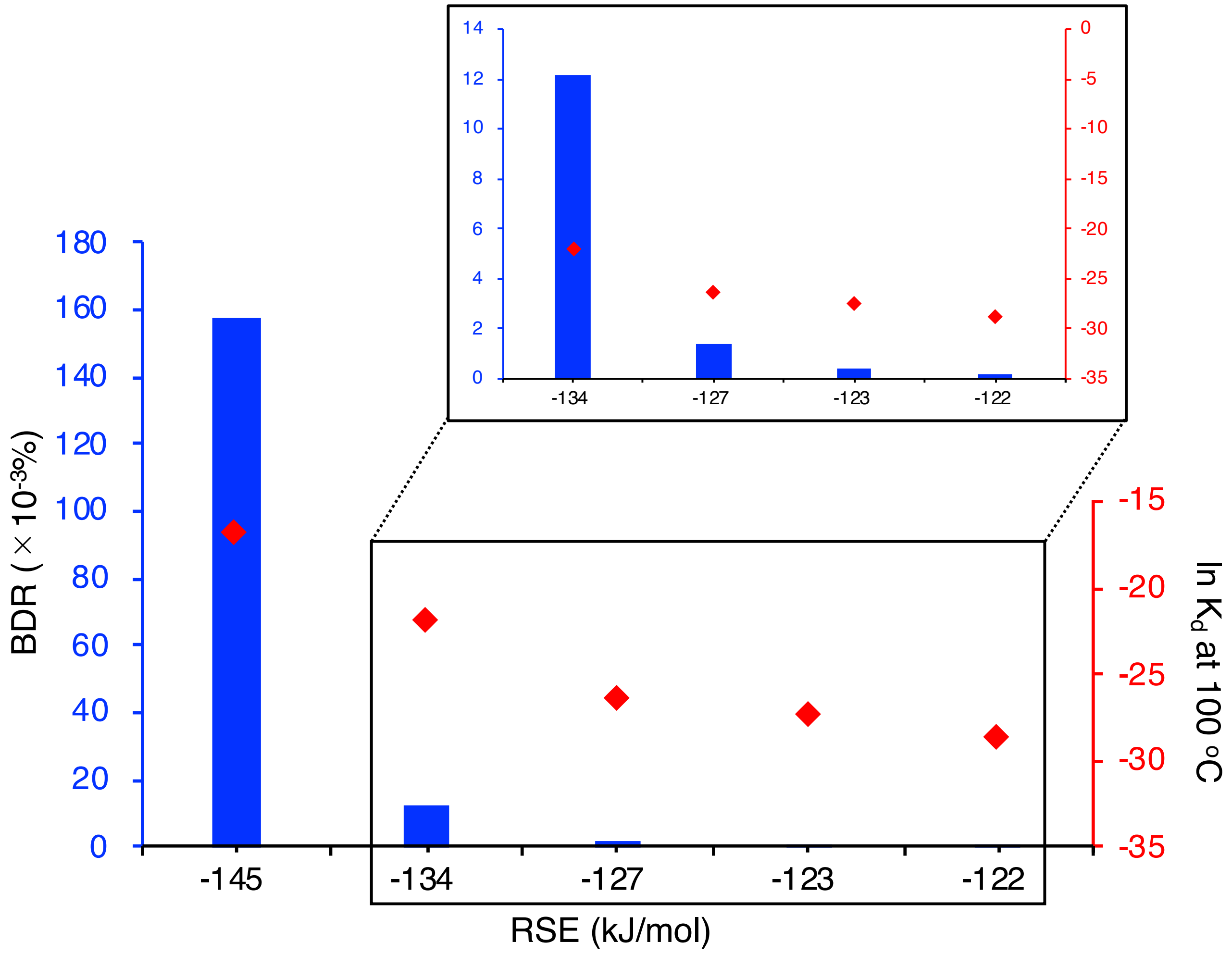

Fig. S30. Relationship between experimental values (BDR and ln K_d) and calculated values (RSE).

5. Mechanochemical reactivity and dynamic covalent property of PMMA-BiACA-4-PMMA

5-1. Grinding experiment

Grinding experiments were carried out at room temperature with a ball-mill device (Retsch Mixer Mill MM 400) with a frequency of 30 Hz. About 50 mg of **PMMA-BiACA-4-PMMA**, together with a stainless ball (d = 5 mm) was charged into the grinding jar. The sample was ground for 60 minutes. No change of optical properties was observed, e.g., color change or fluorescence. The ground sample was collected in an EPR 5 mm quartz capillary, and the capillary was sealed after being degassed. The EPR spectra of the ground samples were measured using a microwave power of 0.2 mW and field modulation of 0.2 mT with a time constant of 0.03 s and a sweep rate of 0.0625 mT/s at room temperature. The concentration of the radicals formed from the cleavage of **PMMA-BiACA-4-PMMA** unit was determined by comparing the area of the observed integral spectrum with a 0.02 mM solution of TEMPOL in benzene under the same experimental conditions. The Mn^{2+} signal was used as an auxiliary standard. The grinding experiments and the EPR measurements were conducted 3 times and the average was used for determination. About 0.102 ± 0.006 % of **PMMA-BiACA-4-PMMA** dissociated.

5-2. Heating experiment

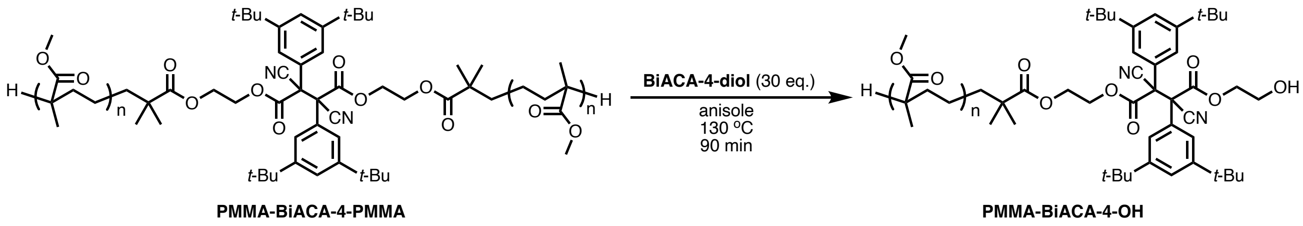

Scheme S4. Radical exchange between **PMMA-BiACA-4-PMMA** and excess amount of **BiACA-4-diol**.

18 mg of **PMMA-BiACA-4-PMMA** (M_n = 24 kDa, PDI = 1.18, 0.0008 mmol) was dissolved in 5 mL of anisole at room temperature. The system was bubbled with nitrogen for 30 minutes. Then, the system was sealed and heated to 130 °C for 90 minutes with an oil bath (first heating). After cooling down to room temperature, the resulting solution was sampled for GPC analysis. Then, to the mixture was added **BiACA-4-diol** (16 mg, 0.0245 mmol, 30 eq.). The mixture was bubbled with nitrogen for 30 minutes. The mixture was sealed and heated to 130 °C for 90 minutes with an oil bath (second heating). After cooling down to room temperature. The resultant was sampled for GPC analysis.

6. Results of computational studies

6-1. Plot of RSE and α-SOMO levels

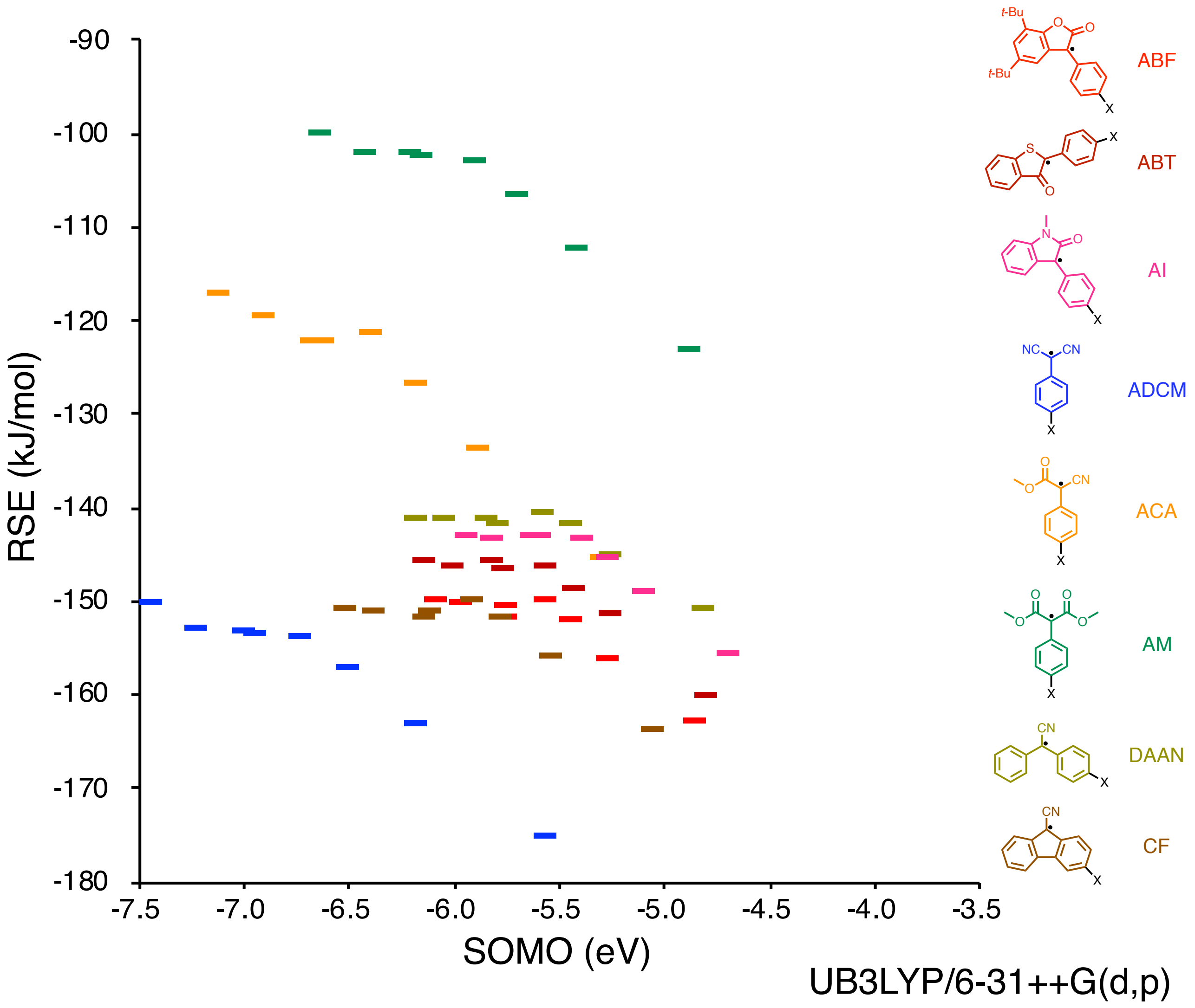

Fig. S31. The relationships between RSE and α-SOMO levels.

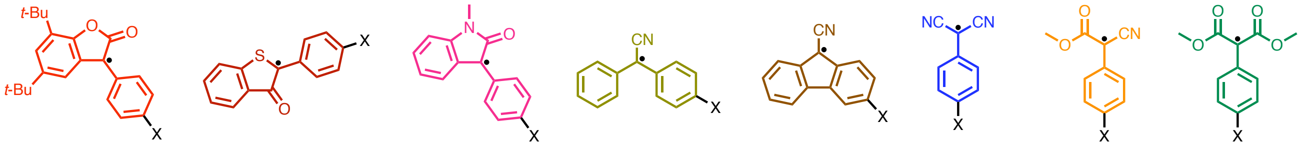

ABF ABT AI DAAN CF ADCM ACA AM

X	$-NMe_2$	-OMe	-Me	-H	-COOMe	-COMe	-CN	$-NO_2$
σ_p	-0.83	-0.27	-0.17	0.00	0.45	0.50	0.66	0.78

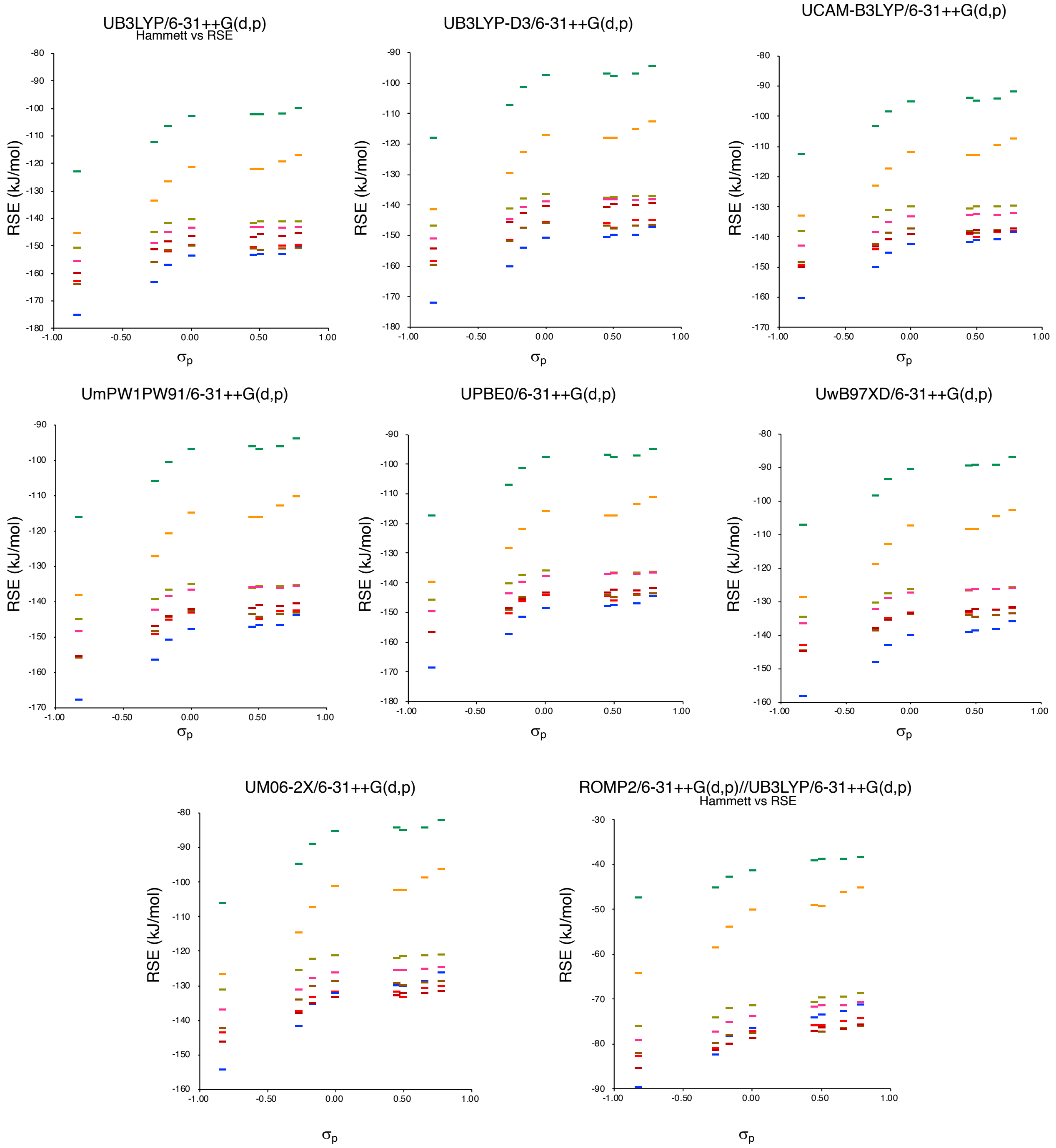

Fig. S32. The relationships between σ_p and RSEs of tested calculated levels. Colors of chemical structures were corresponding to plot colors.

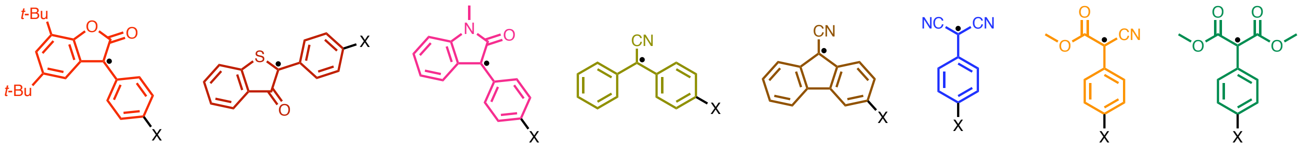

ABF ABT AI DAAN CF ADCM ACA AM

X	$-NMe_2$	-OMe	-Me	-H	-COOMe	-COMe	-CN	$-NO_2$
σ_p	-0.83	-0.27	-0.17	0.00	0.45	0.50	0.66	0.78

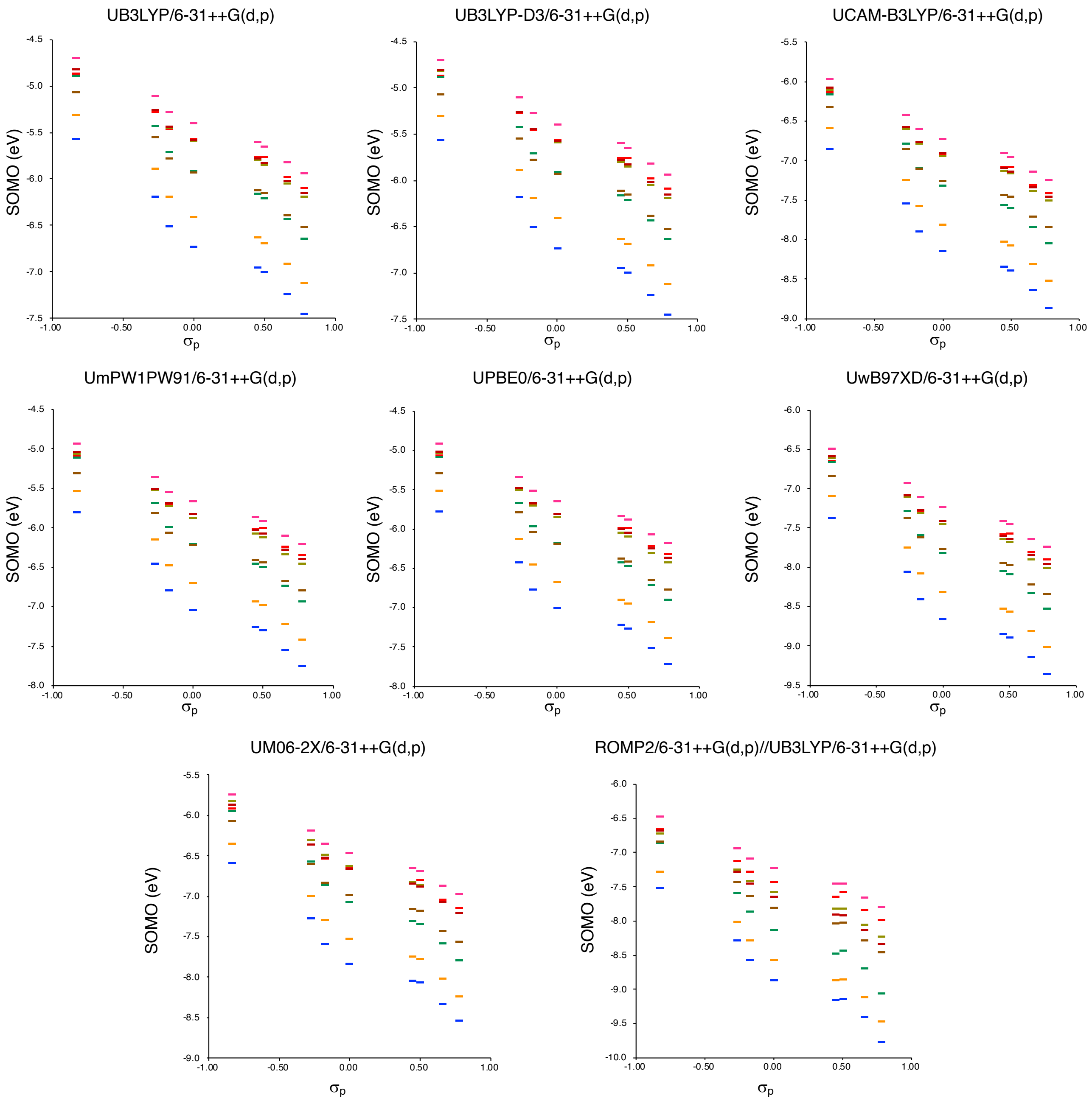

Fig. S33. The relationships between σ_p and α-SOMO levels of tested calculated levels. Colors of chemical structures were corresponding to plot colors.

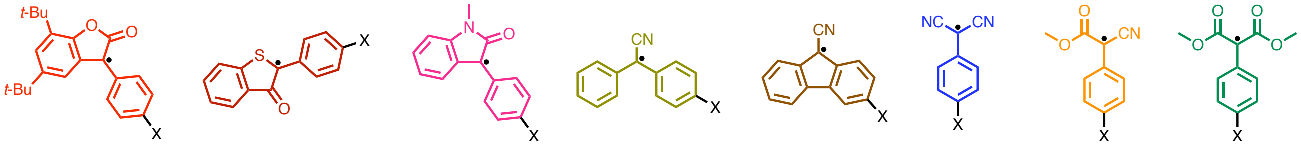

ABF ABT AI DAAN CF ADCM ACA AM

X	$-NMe_2$	-OMe	-Me	-H	-COOMe	-COMe	-CN	$-NO_2$
σ_p	-0.83	-0.27	-0.17	0.00	0.45	0.50	0.66	0.78

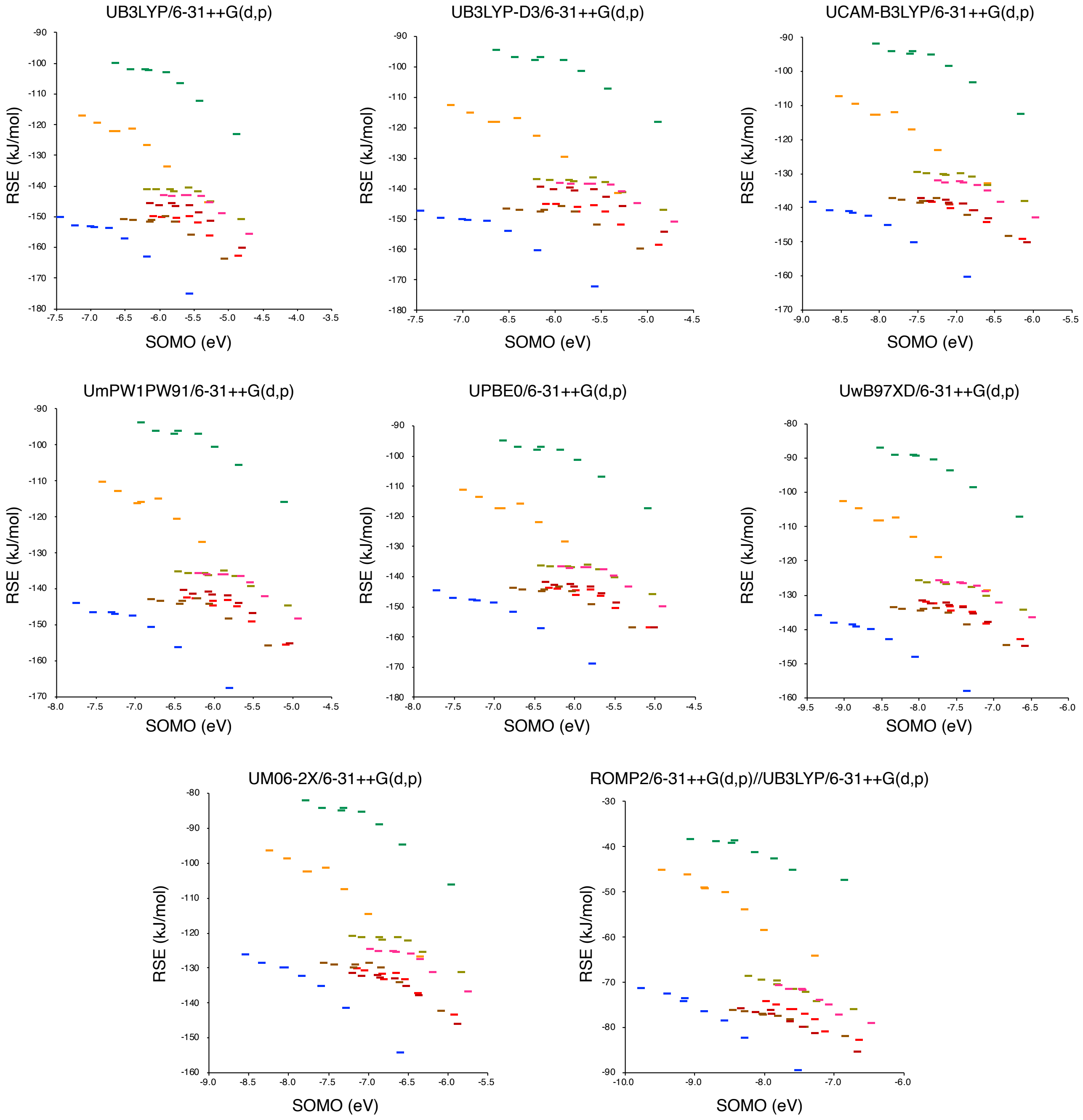

Fig. S34. The relationships between α-SOMO levels and RSEs of tested calculated levels. Colors of chemical structures were corresponding to plot colors.

6-2. Spin density

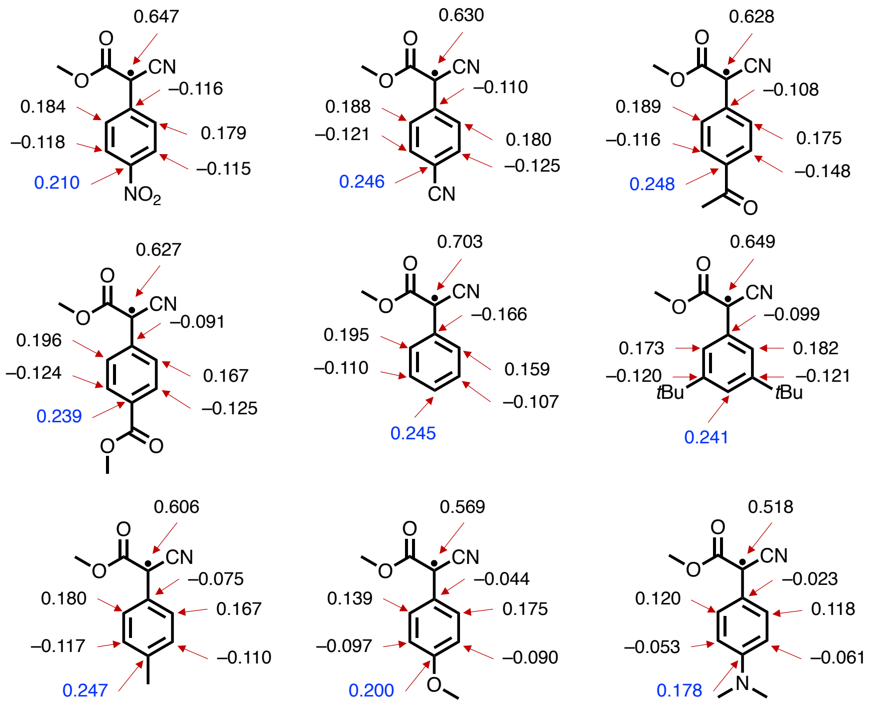

Fig. S35. Spin densities of a series of BiACA calculated at UB3LYP/6-31++G(d,p) level.

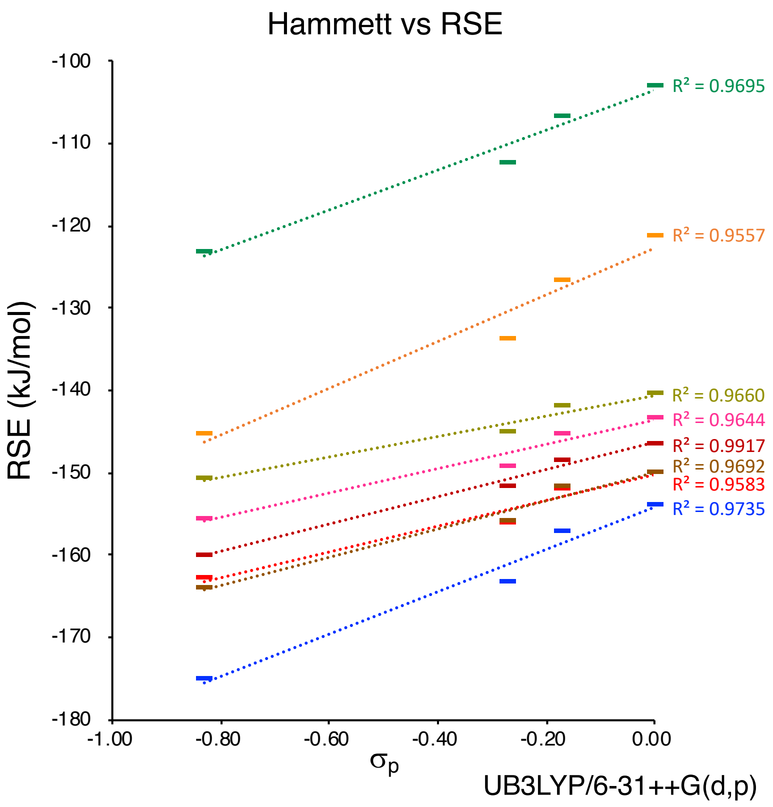

Fig. S36. Linear model of σ_p and RSE for $\sigma_p < 0$.

6-3. All calculated values of ABF, ABT, AI, ADCM, ACA, AM, DAAN, CF and these hydrogenated molecules

UB3LYP/6-31++G(d,p)

	Products	Hammett and Modified Swain-Lupton Constance	SOMO (a.u.)	HF	+CH4	Reactants	HF	+CH3	SOMO (eV)	RSE (kJ/mol)	spin_d of radical centre carbon
	CH4			-40.5261511	—	CH3	-39.8473763	—			
ABF	NO2	0.78	-0.22405	-1208.434991	-1248.961142200	NO2_H	-1209.05673	-1248.904106200	-6.10	-149.75	0.486
	CN	0.66	-0.21976	-1096.170283	-1136.696434100	CN_H	-1096.791905	-1136.639280800	-5.98	-150.06	0.490
	COMe	0.50	-0.21173	-1156.578355	-1197.104506000	COMe_H	-1157.199346	-1197.046721800	-5.76	-151.71	0.470
	COOMe	0.45	-0.21155	-1231.816963	-1272.343113600	COOMe_H	-1232.438458	-1272.285833800	-5.76	-150.39	0.489
	H	0.00	-0.20479	-1003.926279	-1044.452430400	H_H	-1004.547971	-1044.395347600	-5.57	-149.87	0.511
	Me	-0.17	-0.20045	-1043.24805	-1083.774201000	Me_H	-1043.868976	-1083.716352200	-5.45	-151.88	0.487
	OMe	-0.27	-0.19395	-1118.457709	-1158.983860100	OMe_H	-1119.077022	-1158.924398400	-5.28	-156.12	0.460
	NMe2	-0.83	-0.17892	-1137.908215	-1178.434365700	NMe2_H	-1138.524986	-1178.372362500	-4.87	-162.79	0.424
ABT	NO2	0.78	-0.22615	-1216.857882	-1257.384033100	NO2_H	-1217.481221	-1257.328597000	-6.15	-145.55	0.447
	CN	0.66	-0.22111	-1104.593314	-1145.119464600	CN_H	-1105.216378	-1145.063754700	-6.02	-146.27	0.444
	COMe	0.50	-0.21423	-1165.004305	-1205.530456400	COMe_H	-1165.627568	-1205.474944300	-5.83	-145.75	0.449
	COOMe	0.45	-0.21239	-1240.239948	-1280.766099500	COOMe_H	-1240.862874	-1280.710249900	-5.78	-146.63	0.466
	H	0.00	-0.20486	-1012.349257	-1052.875408200	H_H	-1012.972266	-1052.819642300	-5.57	-146.41	0.457
	Me	-0.17	-0.19995	-1051.671179	-1092.197329900	Me_H	-1052.293371	-1092.140747100	-5.44	-148.56	0.441
	OMe	-0.27	-0.1934	-1126.880533	-1167.406684200	OMe_H	-1127.501589	-1167.348965400	-5.26	-151.54	0.431
	NMe2	-0.83	-0.1769	-1146.331746	-1186.857896600	NMe2_H	-1146.949568	-1186.796944500	-4.81	-160.03	0.394
AI	NO2	0.78	-0.21846	-913.3573161	-953.883467200	NO2_H	-913.9815915	-953.828967800	-5.94	-143.09	0.522
	CN	0.66	-0.21402	-801.0924301	-841.618581200	CN_H	-801.7166041	-841.563980400	-5.82	-143.35	0.522
	COMe	0.50	-0.2075	-861.5030825	-902.029233600	COMe_H	-862.1273949	-901.974771200	-5.65	-142.99	0.526
	COOMe	0.45	-0.20573	-936.7383402	-977.264491300	COOMe_H	-937.3626523	-977.210028600	-5.60	-142.99	0.526
	H	0.00	-0.19827	-708.847599	-749.373750100	H_H	-709.4717756	-749.319151900	-5.40	-143.35	0.561
	Me	-0.17	-0.19397	-748.1691985	-788.695349600	Me_H	-748.7926474	-788.640023700	-5.28	-145.26	0.525
	OMe	-0.27	-0.18763	-823.378724	-863.904875100	OMe_H	-824.0007104	-863.848086700	-5.11	-149.10	0.513
	NMe2	-0.83	-0.17274	-842.8287557	-883.354906800	NMe2_H	-843.4482613	-883.295637600	-4.70	-155.61	0.481
ADCM	NO2	0.78	-0.27394	-659.9348857	-700.461036800	NO2_H	-660.5564052	-700.403781500	-7.45	-150.32	0.616
	CN	0.66	-0.26599	-547.6717721	-588.197923200	CN_H	-548.2923243	-588.139700600	-7.24	-152.86	0.601
	COMe	0.50	-0.25731	-608.0850178	-648.611168900	COMe_H	-608.7054939	-648.552870200	-7.00	-153.06	0.604
	COOMe	0.45	-0.25537	-683.3204602	-723.846611300	COOMe_H	-683.9407692	-723.788145500	-6.95	-153.50	0.606
	H	0.00	-0.24754	-455.4317289	-495.957880000	H_H	-456.0519073	-495.899283600	-6.74	-153.84	0.636
	Me	-0.17	-0.23916	-494.7546369	-535.280788000	Me_H	-495.3735829	-535.220959200	-6.51	-157.08	0.583
	OMe	-0.27	-0.22734	-569.9652483	-610.491399400	OMe_H	-570.5818482	-610.429224500	-6.19	-163.24	0.559
	NMe2	-0.83	-0.20466	-589.4192484	-629.945399500	NMe2_H	-590.0313433	-629.878719600	-5.57	-175.07	0.497
ACA	NO2	0.78	-0.26186	-795.5771502	-836.103301300	NO2_H	-796.2113812	-836.058757500	-7.13	-116.95	0.647
	CN	0.66	-0.25417	-683.3136811	-723.839832200	CN_H	-683.9469709	-723.794347200	-6.92	-119.42	0.630
	COMe	0.50	-0.24576	-743.7262706	-784.252421700	COMe_H	-744.3585128	-784.205889100	-6.69	-122.17	0.628
	COOMe	0.45	-0.24385	-818.9612119	-859.487363000	COOMe_H	-819.5934846	-859.440860900	-6.64	-122.09	0.627
	H	0.00	-0.23547	-591.0719675	-631.598118600	H_H	-591.7045669	-631.551943200	-6.41	-121.23	0.703
	Me	-0.17	-0.22762	-630.3947473	-670.920898400	Me_H	-631.0252695	-670.872645800	-6.19	-126.69	0.606
	OMe	-0.27	-0.2165	-705.6054966	-746.131647700	OMe_H	-706.2333737	-746.080750000	-5.89	-133.63	0.569
	NMe2	-0.83	-0.19513	-725.0586553	-765.584806400	NMe2_H	-725.6820713	-765.529447600	-5.31	-145.34	0.518
AM	NO2	0.78	-0.24396	-931.2182279	-971.744379000	NO2_H	-931.8589179	-971.706294200	-6.64	-99.99	0.666
	CN	0.66	-0.23645	-818.9542102	-859.480361300	CN_H	-819.5941661	-859.441542400	-6.43	-101.92	0.651
	COMe	0.50	-0.22828	-879.3654756	-919.891626700	COMe_H	-880.0053447	-919.852721000	-6.21	-102.15	0.658
	COOMe	0.45	-0.22629	-954.600704	-995.126855100	COOMe_H	-955.2405449	-995.087921200	-6.16	-102.22	0.662
	H	0.00	-0.21705	-726.7103905	-767.236541600	H_H	-727.3499791	-767.197355400	-5.91	-102.88	0.650
	Me	-0.17	-0.2098	-766.0327777	-806.558928800	Me_H	-766.6709585	-806.518334800	-5.71	-106.58	0.629
	OMe	-0.27	-0.19948	-841.2431884	-881.769339500	OMe_H	-841.8791707	-881.726547000	-5.43	-112.35	0.589
	NMe2	-0.83	-0.17953	-860.69508	-901.221231100	NMe2_H	-861.3269815	-901.174357800	-4.89	-123.07	0.514
DAAN	NO2	0.78	-0.22748	-798.7708674	-839.297018500	NO2_H	-799.3958973	-839.243273600	-6.19	-141.11	0.584
	CN	0.66	-0.22234	-686.5062697	-727.032420800	CN_H	-687.1312608	-726.978637100	-6.05	-141.21	0.587
	COMe	0.50	-0.21493	-746.9184266	-787.444577700	COMe_H	-747.5433913	-787.390767600	-5.85	-141.28	0.583
	COOMe	0.45	-0.2131	-822.1534933	-862.679644400	COOMe_H	-822.778309	-862.625685300	-5.80	-141.67	0.587
	H	0.00	-0.20534	-594.2629364	-634.789087500	H_H	-594.8882297	-634.735606000	-5.59	-140.42	0.576
	Me	-0.17	-0.20039	-633.584664	-674.110815100	Me_H	-634.20943	-674.056806300	-5.45	-141.80	0.591
	OMe	-0.27	-0.19337	-708.7939464	-749.320097500	OMe_H	-709.4174652	-749.264841500	-5.26	-145.07	0.556
	NMe2	-0.83	-0.17715	-728.2444098	-768.770560900	NMe2_H	-728.8657762	-768.713152500	-4.82	-150.73	0.550
CF	NO2	0.78	-0.23971	-797.5779237	-838.104074800	NO2_H	-798.1993139	-838.046690200	-6.52	-150.66	0.571
	CN	0.66	-0.2347	-685.3133817	-725.839532800	CN_H	-685.9346104	-725.781986700	-6.39	-151.09	0.565
	COMe	0.50	-0.22615	-745.7256823	-786.251833400	COMe_H	-746.3466456	-786.194021900	-6.15	-151.78	0.559
	COOMe	0.45	-0.22475	-820.9606833	-861.486834400	COOMe_H	-821.5819395	-861.429315800	-6.12	-151.02	0.566
	H	0.00	-0.21776	-593.0702374	-633.596388500	H_H	-593.6919134	-633.539289700	-5.93	-149.91	0.573
	Me	-0.17	-0.21244	-632.3917856	-672.917936700	Me_H	-633.0128217	-672.860198000	-5.78	-151.59	0.572
	OMe	-0.27	-0.20379	-707.6014727	-748.127623800	OMe_H	-708.2208465	-748.068222800	-5.55	-155.96	0.543
	NMe2	-0.83	-0.18631	-727.0524504	-767.578601500	NMe2_H	-727.6688094	-767.516185700	-5.07	-163.87	0.498
ACA	H (3,5-t-Bu2)	0.00	-0.22777	-905.6093774	-946.135528500	H (3,5-t-Bu2)_H	-906.2413594	-946.088735700	-6.20	-122.85	0.649

UB3LYP-D3/6-31++G(d,p)

	Products	Hammett and Modified Swain-Lupton Constance	SOMO (a.u.)	HF	+CH4	Reactants	HF	+CH3	SOMO (eV)	RSE (kJ/mol)	spin_d of radical centre carbon
	CH4			-40.526308300	—	CH3	-39.8474927	—			
ABF	NO2	0.78	-0.22398	-1208.492528	-1249.018836200	NO2_H	-1209.116085	-1248.963577400	-6.09	-145.08	0.486
	CN	0.66	-0.21968	-1096.225899	-1136.752207500	CN_H	-1096.849399	-1136.696892000	-5.98	-145.23	0.491
	COMe	0.50	-0.21162	-1156.638352	-1197.164660700	COMe_H	-1157.260949	-1197.108442100	-5.76	-147.60	0.469
	COOMe	0.45	-0.21149	-1231.877818	-1272.404126400	COOMe_H	-1232.501036	-1272.348529000	-5.75	-145.97	0.489
	H	0.00	-0.20473	-1003.980072	-1044.506380200	H_H	-1004.603445	-1044.450937400	-5.57	-145.57	0.511
	Me	-0.17	-0.20039	-1043.304619	-1083.830927100	Me_H	-1043.927222	-1083.774715100	-5.45	-147.58	0.487
	OMe	-0.27	-0.19394	-1118.515863	-1159.042171300	OMe_H	-1119.136875	-1158.984367900	-5.28	-151.76	0.461
	NMe2	-0.83	-0.17897	-1137.970497	-1178.496805100	NMe2_H	-1138.588943	-1178.436435900	-4.87	-158.50	0.435
ABT	NO2	0.78	-0.22619	-1216.88264	-1257.408948300	NO2_H	-1217.508359	-1257.355851500	-6.15	-139.41	0.448
	CN	0.66	-0.22111	-1104.616167	-1145.142475200	CN_H	-1105.241601	-1145.089093600	-6.02	-140.15	0.445
	COMe	0.50	-0.21427	-1165.031537	-1205.557845200	COMe_H	-1165.657151	-1205.504644100	-5.83	-139.68	0.450
	COOMe	0.45	-0.2124	-1240.268018	-1280.794326500	COOMe_H	-1240.893316	-1280.740809000	-5.78	-140.51	0.467
	H	0.00	-0.20487	-1012.370283	-1052.896591700	H_H	-1012.995622	-1052.843114600	-5.57	-140.40	0.458
	Me	-0.17	-0.19993	-1051.695001	-1092.221309400	Me_H	-1052.319482	-1092.166974200	-5.44	-142.66	0.442
	OMe	-0.27	-0.19335	-1126.905976	-1167.432284300	OMe_H	-1127.529292	-1167.376785000	-5.26	-145.71	0.432
	NMe2	-0.83	-0.17683	-1146.361299	-1186.887606800	NMe2_H	-1146.98133	-1186.828822900	-4.81	-154.34	0.395
AI	NO2	0.78	-0.2183	-913.3864107	-953.912719000	NO2_H	-914.012584	-953.860076700	-5.94	-138.21	0.522
	CN	0.66	-0.21388	-801.1196054	-841.645913700	CN_H	-801.7456774	-841.593170100	-5.82	-138.48	0.522
	COMe	0.50	-0.20741	-861.5346506	-902.060958900	COMe_H	-862.1607615	-902.008254200	-5.64	-138.38	0.525
	COOMe	0.45	-0.20561	-936.7707489	-977.297057200	COOMe_H	-937.3968607	-977.244353400	-5.59	-138.37	0.526
	H	0.00	-0.19816	-708.872954	-749.399262300	H_H	-709.498891	-749.346383700	-5.39	-138.83	0.562
	Me	-0.17	-0.19387	-748.1973187	-788.723627000	Me_H	-748.8225254	-788.670018100	-5.28	-140.75	0.526
	OMe	-0.27	-0.1876	-823.4084234	-863.934731700	OMe_H	-824.0321229	-863.879615600	-5.10	-144.71	0.514
	NMe2	-0.83	-0.17279	-842.8625025	-883.388810800	NMe2_H	-843.4838201	-883.331312800	-4.70	-150.96	0.483
ADCM	NO2	0.78	-0.27389	-659.9495857	-700.475894000	NO2_H	-660.5723464	-700.419839100	-7.45	-147.17	0.616
	CN	0.66	-0.26591	-547.6845751	-588.210883400	CN_H	-548.3063612	-588.153853900	-7.24	-149.73	0.600
	COMe	0.50	-0.25729	-608.1021731	-648.628481400	COMe_H	-608.7238854	-648.571378100	-7.00	-149.92	0.603
	COOMe	0.45	-0.25531	-683.3384646	-723.864772900	COOMe_H	-683.9599987	-723.807491400	-6.95	-150.39	0.606
	H	0.00	-0.24747	-455.4427083	-495.969016600	H_H	-456.0641058	-495.911598500	-6.73	-150.75	0.635
	Me	-0.17	-0.23905	-494.7683972	-535.294705500	Me_H	-495.3885425	-535.236035200	-6.50	-154.04	0.583
	OMe	-0.27	-0.22723	-569.9806273	-610.506935600	OMe_H	-570.5984146	-610.445907300	-6.18	-160.23	0.558
	NMe2	-0.83	-0.20454	-589.4387093	-629.965017600	NMe2_H	-590.0519743	-629.899467000	-5.57	-172.10	0.496
ACA	NO2	0.78	-0.26179	-795.5973134	-836.123621700	NO2_H	-796.2332132	-836.080705900	-7.12	-112.68	0.646
	CN	0.66	-0.25413	-683.3319399	-723.858248200	CN_H	-683.9668901	-723.814382800	-6.92	-115.17	0.629
	COMe	0.50	-0.24578	-743.7488948	-784.275203100	COMe_H	-744.3827028	-784.230195500	-6.69	-118.17	0.627
	COOMe	0.45	-0.24383	-818.9846809	-859.510989200	COOMe_H	-819.6185147	-859.466007400	-6.64	-118.10	0.626
	H	0.00	-0.23544	-591.0884025	-631.614710800	H_H	-591.7226347	-631.570127400	-6.41	-117.05	0.702
	Me	-0.17	-0.22756	-630.4139644	-670.940272700	Me_H	-631.0460211	-670.893513800	-6.19	-122.77	0.605
	OMe	-0.27	-0.21643	-705.6263436	-746.152651900	OMe_H	-706.2557282	-746.103220900	-5.89	-129.78	0.568
	NMe2	-0.83	-0.19506	-725.0836064	-765.609914700	NMe2_H	-725.7085057	-765.555998400	-5.31	-141.56	0.517
AM	NO2	0.78	-0.24394	-931.244116	-971.770424300	NO2_H	-931.886881	-971.734373700	-6.64	-94.65	0.661
	CN	0.66	-0.23639	-818.9782037	-859.504512000	CN_H	-819.6201351	-859.467627800	-6.43	-96.84	0.646
	COMe	0.50	-0.2282	-879.3941704	-919.920478700	COMe_H	-880.0357477	-919.883240400	-6.21	-97.77	0.656
	COOMe	0.45	-0.22628	-954.6298903	-995.156198600	COOMe_H	-955.271792	-995.119284700	-6.16	-96.92	0.658
	H	0.00	-0.21705	-726.7325556	-767.258863900	H_H	-727.3741574	-767.221650100	-5.91	-97.70	0.647
	Me	-0.17	-0.20975	-766.0577471	-806.584055400	Me_H	-766.6979095	-806.545402200	-5.71	-101.48	0.623
	OMe	-0.27	-0.19942	-841.2697813	-881.796089600	OMe_H	-841.9077391	-881.755231800	-5.43	-107.27	0.584
	NMe2	-0.83	-0.17945	-860.7257773	-901.252085600	NMe2_H	-861.3596316	-901.207124300	-4.88	-118.05	0.516
DAAN	NO2	0.78	-0.22742	-798.7948158	-839.321124100	NO2_H	-799.4214371	-839.268929800	-6.19	-137.04	0.583
	CN	0.66	-0.22225	-686.5283066	-727.054614900	CN_H	-687.1548798	-727.002372500	-6.05	-137.16	0.587
	COMe	0.50	-0.21488	-746.9448348	-787.471143100	COMe_H	-747.5713642	-787.418856900	-5.85	-137.28	0.583
	COOMe	0.45	-0.21303	-822.1807469	-862.707055200	COOMe_H	-822.8071308	-862.654623500	-5.80	-137.66	0.587
	H	0.00	-0.20527	-594.2831416	-634.809449900	H_H	-594.9099719	-634.757464600	-5.59	-136.49	0.575
	Me	-0.17	-0.20029	-633.6076454	-674.133953700	Me_H	-634.2339382	-674.081430900	-5.45	-137.90	0.591
	OMe	-0.27	-0.19329	-708.8185362	-749.344844500	OMe_H	-709.4435815	-749.291074200	-5.26	-141.17	0.555
	NMe2	-0.83	-0.17705	-728.2730807	-768.799389000	NMe2_H	-728.8959713	-768.743464000	-4.82	-146.83	0.552
CF	NO2	0.78	-0.23971	-797.5994321	-838.125740400	NO2_H	-798.2224182	-838.069910900	-6.52	-146.58	0.570
	CN	0.66	-0.23465	-685.3330007	-725.859309000	CN_H	-685.9558166	-725.803309300	-6.39	-147.03	0.564
	COMe	0.50	-0.22617	-745.7496744	-786.275982700	COMe_H	-746.3722288	-786.219721500	-6.15	-147.71	0.559
	COOMe	0.45	-0.22473	-820.9855314	-861.511839700	COOMe_H	-821.6083754	-861.455868100	-6.12	-146.95	0.565
	H	0.00	-0.21772	-593.0879682	-633.614276500	H_H	-593.7112279	-633.558720600	-5.92	-145.86	0.572
	Me	-0.17	-0.21237	-632.4123556	-672.938663900	Me_H	-633.0349751	-672.882467800	-5.78	-147.54	0.571
	OMe	-0.27	-0.20371	-707.623623	-748.149931300	OMe_H	-708.2445734	-748.092066100	-5.54	-151.93	0.542
	NMe2	-0.83	-0.18622	-727.0788323	-767.605140600	NMe2_H	-727.6967986	-767.544291300	-5.07	-159.76	0.497
ACA	H (3,5-t-Bu2)	0.00	-0.22783	-905.6584345	-946.184742800	H (3,5-t-Bu2)_H	-906.2917255	-946.139218200	-6.20	-119.52	0.649

UCAM-B3LYP/6-31++G(d,p)

	Products	Hammett and Modified Swain-Lupton Constance	SOMO (a.u.)	HF	+CH4	Reactants	HF	+CH3	SOMO (eV)	RSE (kJ/mol)	spin_d of radical centre carbon
	CH4			-40.4918788	—	CH3	-39.8158012	—			
ABF	NO2	0.78	-0.27255	-1207.814731	-1248.306610000	NO2_H	-1208.438324	-1248.254125100	-7.42	-137.80	0.564
	CN	0.66	-0.26872	-1095.572057	-1136.063935900	CN_H	-1096.195476	-1136.011276900	-7.31	-138.26	0.558
	COMe	0.50	-0.26013	-1155.954248	-1196.446126700	COMe_H	-1156.576955	-1196.392756400	-7.08	-140.12	0.539
	COOMe	0.45	-0.26039	-1231.174041	-1271.665919800	COOMe_H	-1231.797194	-1271.612994800	-7.09	-138.95	0.559
	H	0.00	-0.25412	-1003.36953	-1043.861408800	H_H	-1003.992741	-1043.808541700	-6.92	-138.80	0.579
	Me	-0.17	-0.2494	-1042.666012	-1083.157890800	Me_H	-1043.288551	-1083.104352100	-6.79	-140.57	0.559
	OMe	-0.27	-0.24259	-1117.855602	-1158.347480400	OMe_H	-1118.476813	-1158.292614000	-6.60	-144.05	0.534
	NMe2	-0.83	-0.22559	-1137.277762	-1177.769640600	NMe2_H	-1137.897085	-1177.712886200	-6.14	-149.01	0.498
ABT	NO2	0.78	-0.27431	-1216.447549	-1256.939428100	NO2_H	-1217.071449	-1256.887250100	-7.46	-136.99	0.508
	CN	0.66	-0.2698	-1104.205068	-1144.696947100	CN_H	-1104.828588	-1144.644389500	-7.34	-137.99	0.505
	COMe	0.50	-0.2624	-1164.590308	-1205.082186800	COMe_H	-1165.213973	-1205.029774600	-7.14	-137.61	0.508
	COOMe	0.45	-0.2609	-1239.806874	-1280.298752500	COOMe_H	-1240.430239	-1280.246040100	-7.10	-138.40	0.511
	H	0.00	-0.25395	-1012.002417	-1052.494296100	H_H	-1012.625638	-1052.441439500	-6.91	-138.78	0.513
	Me	-0.17	-0.24868	-1051.29904	-1091.790918500	Me_H	-1051.921529	-1091.737330100	-6.77	-140.70	0.499
	OMe	-0.27	-0.24187	-1126.488292	-1166.980170600	OMe_H	-1127.109927	-1166.925728500	-6.58	-142.94	0.488
	NMe2	-0.83	-0.22332	-1145.911186	-1186.403064700	NMe2_H	-1146.530143	-1186.345944500	-6.08	-149.97	0.458
AI	NO2	0.78	-0.26626	-912.9065478	-953.398426600	NO2_H	-913.5323568	-953.348158000	-7.25	-131.98	0.607
	CN	0.66	-0.26244	-800.6637801	-841.155658900	CN_H	-801.289371	-841.105172200	-7.14	-132.55	0.603
	COMe	0.50	-0.25546	-861.048653	-901.540531800	COMe_H	-861.6743458	-901.490147000	-6.95	-132.29	0.601
	COOMe	0.45	-0.25401	-936.2649095	-976.756788300	COOMe_H	-936.8905641	-976.706365300	-6.91	-132.39	0.600
	H	0.00	-0.24714	-708.460368	-748.952246800	H_H	-709.0857314	-748.901532600	-6.73	-133.15	0.636
	Me	-0.17	-0.24254	-747.756659	-788.248537800	Me_H	-748.3814057	-788.197206900	-6.60	-134.77	0.597
	OMe	-0.27	-0.236	-822.9461608	-863.438039600	OMe_H	-823.5696359	-863.385437100	-6.42	-138.11	0.593
	NMe2	-0.83	-0.2194	-842.3678739	-882.859752700	NMe2_H	-842.9895625	-882.805363700	-5.97	-142.80	0.563
ADCM	NO2	0.78	-0.32581	-659.6198586	-700.111737400	NO2_H	-660.2433149	-700.059116100	-8.87	-138.16	0.701
	CN	0.66	-0.31765	-547.3785964	-587.870475200	CN_H	-548.0011366	-587.816937800	-8.64	-140.56	0.680
	COMe	0.50	-0.30836	-607.7662644	-648.258143200	COMe_H	-608.3886553	-648.204456500	-8.39	-140.95	0.675
	COOMe	0.45	-0.3069	-682.9826033	-723.474482100	COOMe_H	-683.6048114	-723.420612600	-8.35	-141.43	0.678
	H	0.00	-0.29937	-455.1799838	-495.671862600	H_H	-455.8019275	-495.617728700	-8.15	-142.13	0.720
	Me	-0.17	-0.29011	-494.4775635	-534.969442300	Me_H	-495.0984181	-534.914219300	-7.89	-144.99	0.658
	OMe	-0.27	-0.27743	-569.6678957	-610.159774500	OMe_H	-570.2868292	-610.102630400	-7.55	-150.03	0.630
	NMe2	-0.83	-0.25199	-589.0935262	-629.585405000	NMe2_H	-589.7085909	-629.524392100	-6.86	-160.19	0.562
ACA	NO2	0.78	-0.31348	-795.2176106	-835.709489400	NO2_H	-795.852877	-835.668678200	-8.53	-107.15	0.715
	CN	0.66	-0.30559	-682.97601	-723.467888800	CN_H	-683.6104127	-723.426213900	-8.32	-109.42	0.700
	COMe	0.50	-0.29657	-743.362984	-783.854862800	COMe_H	-743.9961695	-783.811970700	-8.07	-112.61	0.694
	COOMe	0.45	-0.29513	-818.5788204	-859.070699200	COOMe_H	-819.2120432	-859.027844400	-8.03	-112.52	0.693
	H	0.00	-0.2871	-590.7757095	-631.267588300	H_H	-591.4091762	-631.224977400	-7.81	-111.87	0.783
	Me	-0.17	-0.27835	-630.0731608	-670.565039600	Me_H	-630.7046661	-670.520467300	-7.57	-117.02	0.669
	OMe	-0.27	-0.26633	-705.2636881	-745.755566900	OMe_H	-705.892944	-745.708745200	-7.25	-122.93	0.635
	NMe2	-0.83	-0.24218	-724.6884275	-765.180306300	NMe2_H	-725.3139338	-765.129735000	-6.59	-132.78	0.583
AM	NO2	0.78	-0.29593	-930.8138432	-971.305722000	NO2_H	-931.4550073	-971.270808500	-8.05	-91.67	0.714
	CN	0.66	-0.28819	-818.5718597	-859.063738500	CN_H	-819.2121677	-859.027968900	-7.84	-93.91	0.696
	COMe	0.50	-0.27937	-878.9577124	-919.449591200	COMe_H	-879.5977166	-919.413517800	-7.60	-94.71	0.711
	COOMe	0.45	-0.27797	-954.1735304	-994.665409200	COOMe_H	-954.8138679	-994.629669100	-7.56	-93.84	0.712
	H	0.00	-0.2692	-726.3694217	-766.861300500	H_H	-727.009375	-766.825176200	-7.33	-94.84	0.691
	Me	-0.17	-0.26089	-765.6665323	-806.158411100	Me_H	-766.3051772	-806.120978400	-7.10	-98.28	0.668
	OMe	-0.27	-0.24957	-840.8568397	-881.348718500	OMe_H	-841.4936215	-881.309422700	-6.79	-103.17	0.628
	NMe2	-0.83	-0.22645	-860.2804313	-900.772310100	NMe2_H	-860.9137487	-900.729549900	-6.16	-112.27	0.560
DAAN	NO2	0.78	-0.27599	-798.3615133	-838.853392100	NO2_H	-798.9883219	-838.804123100	-7.51	-129.36	0.649
	CN	0.66	-0.2715	-686.1190476	-726.610926400	CN_H	-686.7456993	-726.561500500	-7.39	-129.77	0.654
	COMe	0.50	-0.26353	-746.50549	-786.997368800	COMe_H	-747.1321088	-786.947910000	-7.17	-129.85	0.643
	COOMe	0.45	-0.2621	-821.721535	-862.213413800	COOMe_H	-822.34796	-862.163761200	-7.13	-130.36	0.645
	H	0.00	-0.255	-593.9171911	-634.409069900	H_H	-594.5438857	-634.359686900	-6.94	-129.66	0.616
	Me	-0.17	-0.24967	-633.2136485	-673.705527300	Me_H	-633.8398753	-673.655676500	-6.79	-130.88	0.652
	OMe	-0.27	-0.24245	-708.402821	-748.894699800	OMe_H	-709.0281256	-748.843926800	-6.60	-133.30	0.656
	NMe2	-0.83	-0.22447	-727.8251697	-768.317048500	NMe2_H	-728.4486978	-768.264499000	-6.11	-137.97	0.617
CF	NO2	0.78	-0.288	-797.1741738	-837.666052600	NO2_H	-797.7980283	-837.613829500	-7.84	-137.11	0.650
	CN	0.66	-0.28342	-684.9316479	-725.423526700	CN_H	-685.555272	-725.371073200	-7.71	-137.72	0.644
	COMe	0.50	-0.27438	-745.3182765	-785.810155300	COMe_H	-745.9416415	-785.757442700	-7.47	-138.40	0.636
	COOMe	0.45	-0.2733	-820.5342508	-861.026129600	COOMe_H	-821.1578531	-860.973654300	-7.44	-137.77	0.642
	H	0.00	-0.26676	-592.7299246	-633.221803400	H_H	-593.3537653	-633.169566500	-7.26	-137.15	0.646
	Me	-0.17	-0.26106	-632.0262041	-672.518082900	Me_H	-632.6494938	-672.465295000	-7.10	-138.59	0.648
	OMe	-0.27	-0.25205	-707.2157517	-747.707630500	OMe_H	-707.8377271	-747.653528300	-6.86	-142.05	0.623
	NMe2	-0.83	-0.23237	-726.6383617	-767.130240500	NMe2_H	-727.258015	-767.073816200	-6.32	-148.14	0.584
ACA	H (3,5-t-Bu2)	0.00	-0.27965	-905.1156514	-945.607530200	H (3,5-t-Bu2)_H	-905.7485234	-945.564324600	-7.61	-113.44	0.713

UmPW1PW91/6-31++G(d,p)

	Products	Hammett and Modified Swain-Lupton Constance	SOMO (a.u.)	HF	+CH4	Reactants	HF	+CH3	SOMO (eV)	RSE (kJ/mol)	spin_d of radical centre carbon
	CH4			-40.51084	–	CH3	-39.8336875	–			
ABF	NO2	0.78	-0.233	-1208.151077	-1248.661916700	NO2_H	-1208.773995	-1248.607682500	-6.34	-142.39	0.544
	CN	0.66	-0.22921	-1095.910041	-1136.420881200	CN_H	-1096.532828	-1136.366515200	-6.24	-142.74	0.539
	COMe	0.50	-0.22082	-1156.30616	-1196.816999800	COMe_H	-1156.928167	-1196.761854200	-6.01	-144.78	0.522
	COOMe	0.45	-0.22086	-1231.525549	-1272.036388800	COOMe_H	-1232.148084	-1271.981771000	-6.01	-143.40	0.543
	H	0.00	-0.21416	-1003.692539	-1044.203378600	H_H	-1004.315196	-1044.148883700	-5.83	-143.08	0.567
	Me	-0.17	-0.2096	-1043.005429	-1083.516269100	Me_H	-1043.627326	-1083.461013600	-5.70	-145.07	0.543
	OMe	-0.27	-0.20282	-1118.195067	-1158.705907100	OMe_H	-1118.815455	-1158.649142800	-5.52	-149.03	0.512
	NMe2	-0.83	-0.18685	-1137.643019	-1178.153859400	NMe2_H	-1138.260982	-1178.094669500	-5.08	-155.40	0.473
ABT	NO2	0.78	-0.23492	-1216.667293	-1257.178133100	NO2_H	-1217.290972	-1257.124659700	-6.39	-140.39	0.488
	CN	0.66	-0.23044	-1104.426463	-1144.937303300	CN_H	-1105.049799	-1144.883486500	-6.27	-141.30	0.484
	COMe	0.50	-0.22326	-1164.825538	-1205.336378000	COMe_H	-1165.448986	-1205.282673800	-6.08	-141.00	0.490
	COOMe	0.45	-0.22156	-1240.041848	-1280.552687800	COOMe_H	-1240.665029	-1280.498716500	-6.03	-141.70	0.491
	H	0.00	-0.21414	-1012.208908	-1052.719747800	H_H	-1012.832017	-1052.665704000	-5.83	-141.89	0.498
	Me	-0.17	-0.20903	-1051.521953	-1092.032792900	Me_H	-1052.144228	-1091.977915000	-5.69	-144.08	0.481
	OMe	-0.27	-0.20225	-1126.711298	-1167.222137500	OMe_H	-1127.332499	-1167.166186300	-5.50	-146.90	0.470
	NMe2	-0.83	-0.18501	-1146.159985	-1186.670824900	NMe2_H	-1146.778018	-1186.611705100	-5.03	-155.22	0.435
AI	NO2	0.78	-0.22777	-913.1399275	-953.650767500	NO2_H	-913.7654268	-953.599114300	-6.20	-135.62	0.590
	CN	0.66	-0.2239	-800.8987994	-841.409639400	CN_H	-801.5241317	-841.357819200	-6.09	-136.05	0.587
	COMe	0.50	-0.21708	-861.2975236	-901.808363600	COMe_H	-861.9229122	-901.756599700	-5.91	-135.91	0.593
	COOMe	0.45	-0.21545	-936.5135115	-977.024351500	COOMe_H	-937.1388865	-976.972574000	-5.86	-135.94	0.593
	H	0.00	-0.20811	-708.6804846	-749.191324600	H_H	-709.3056225	-749.139310000	-5.66	-136.56	0.632
	Me	-0.17	-0.2036	-747.9931795	-788.504019500	Me_H	-748.6176259	-788.451313400	-5.54	-138.38	0.587
	OMe	-0.27	-0.19696	-823.1827238	-863.693563800	OMe_H	-823.805694	-863.639381500	-5.36	-142.26	0.574
	NMe2	-0.83	-0.18106	-842.6302237	-883.141063700	NMe2_H	-843.2508698	-883.084557300	-4.93	-148.36	0.540
ADCM	NO2	0.78	-0.28486	-659.7627769	-700.273616900	NO2_H	-660.3851366	-700.218824100	-7.75	-143.86	0.701
	CN	0.66	-0.27723	-547.5232701	-588.034110100	CN_H	-548.1446351	-587.978322600	-7.54	-146.47	0.674
	COMe	0.50	-0.26821	-607.924666	-648.435506000	COMe_H	-608.5459644	-648.379651900	-7.30	-146.65	0.674
	COOMe	0.45	-0.26646	-683.1407861	-723.651626100	COOMe_H	-683.7619158	-723.595603300	-7.25	-147.09	0.677
	H	0.00	-0.2586	-455.3097618	-495.820601800	H_H	-455.930724	-495.764411500	-7.04	-147.53	0.717
	Me	-0.17	-0.24966	-494.6237757	-535.134615700	Me_H	-495.2435482	-535.077235700	-6.79	-150.65	0.654
	OMe	-0.27	-0.23718	-569.8142509	-610.325090900	OMe_H	-570.4318895	-610.265577000	-6.45	-156.25	0.628
	NMe2	-0.83	-0.21331	-589.2657082	-629.776548200	NMe2_H	-589.879026	-629.712713500	-5.80	-167.60	0.557
ACA	NO2	0.78	-0.27254	-795.3739619	-835.884801900	NO2_H	-796.0090881	-835.842775600	-7.42	-110.34	0.722
	CN	0.66	-0.26519	-683.1341002	-723.644940200	CN_H	-683.768306	-723.601993500	-7.22	-112.76	0.703
	COMe	0.50	-0.25639	-743.5348197	-784.045659700	COMe_H	-744.167742	-784.001429500	-6.98	-116.13	0.700
	COOMe	0.45	-0.25468	-818.7504373	-859.261277300	COOMe_H	-819.3833949	-859.217082400	-6.93	-116.03	0.699
	H	0.00	-0.24624	-590.918886	-631.429726000	H_H	-591.552293	-631.385980500	-6.70	-114.85	0.798
	Me	-0.17	-0.23784	-630.2327542	-670.743594200	Me_H	-630.8639579	-670.697645400	-6.47	-120.64	0.676
	OMe	-0.27	-0.22603	-705.4233936	-745.934233600	OMe_H	-706.0521257	-745.885813200	-6.15	-127.13	0.638
	NMe2	-0.83	-0.20349	-724.8739347	-765.384774700	NMe2_H	-725.4984522	-765.332139700	-5.54	-138.19	0.586
AM	NO2	0.78	-0.25469	-930.9837425	-971.494582500	NO2_H	-931.6251187	-971.458806200	-6.93	-93.93	0.732
	CN	0.66	-0.24755	-818.7434234	-859.254263400	CN_H	-819.3839532	-859.217640700	-6.74	-96.15	0.711
	COMe	0.50	-0.23885	-879.1430422	-919.653882200	COMe_H	-879.783282	-919.616969500	-6.50	-96.91	0.726
	COOMe	0.45	-0.23718	-954.3586269	-994.869466900	COOMe_H	-954.9991931	-994.832880600	-6.45	-96.06	0.728
	H	0.00	-0.22788	-726.5260032	-767.036843200	H_H	-727.1662529	-766.999940400	-6.20	-96.89	0.713
	Me	-0.17	-0.22004	-765.8394962	-806.350336200	Me_H	-766.4783875	-806.312075000	-5.99	-100.45	0.686
	OMe	-0.27	-0.20896	-841.0298155	-881.540655500	OMe_H	-841.6666663	-881.500353800	-5.69	-105.81	0.645
	NMe2	-0.83	-0.18765	-860.479109	-900.989949000	NMe2_H	-861.1120816	-900.945769100	-5.11	-115.99	0.573
DAAN	NO2	0.78	-0.23726	-798.5723814	-839.083221400	NO2_H	-799.1979861	-839.031673600	-6.46	-135.34	0.641
	CN	0.66	-0.23273	-686.3315392	-726.842379200	CN_H	-686.9570151	-726.790702600	-6.33	-135.68	0.646
	COMe	0.50	-0.22495	-746.731779	-787.242619000	COMe_H	-747.3572562	-787.190943700	-6.12	-135.67	0.642
	COOMe	0.45	-0.22329	-821.9475577	-862.458397700	COOMe_H	-822.5728611	-862.406548600	-6.08	-136.13	0.647
	H	0.00	-0.21566	-594.1147233	-634.625563300	H_H	-594.7404495	-634.574137000	-5.87	-135.02	0.626
	Me	-0.17	-0.21043	-633.4276062	-673.938446200	Me_H	-634.0527762	-673.886463700	-5.73	-136.48	0.647
	OMe	-0.27	-0.20306	-708.6168002	-749.127640200	OMe_H	-709.2409025	-749.074590000	-5.53	-139.28	0.648
	NMe2	-0.83	-0.1858	-728.0648134	-768.575653400	NMe2_H	-728.6868317	-768.520519200	-5.06	-144.75	0.609
CF	NO2	0.78	-0.24984	-797.3850524	-837.895892400	NO2_H	-798.0077319	-837.841419400	-6.80	-143.02	0.643
	CN	0.66	-0.24532	-685.1441751	-725.655015100	CN_H	-685.7666431	-725.600330600	-6.68	-143.57	0.633
	COMe	0.50	-0.23643	-745.5446589	-786.055498900	COMe_H	-746.1668443	-786.000531800	-6.43	-144.32	0.627
	COOMe	0.45	-0.23519	-820.7603816	-861.271221600	COOMe_H	-821.3828531	-861.216540600	-6.40	-143.57	0.634
	H	0.00	-0.2283	-592.9275979	-633.438437900	H_H	-593.5504238	-633.384111300	-6.21	-142.63	0.640
	Me	-0.17	-0.22267	-632.2403025	-672.751142500	Me_H	-632.8625011	-672.696188600	-6.06	-144.28	0.639
	OMe	-0.27	-0.21355	-707.4299316	-747.940771600	OMe_H	-708.0505637	-747.884251200	-5.81	-148.39	0.610
	NMe2	-0.83	-0.19501	-726.8784327	-767.389272700	NMe2_H	-727.4962061	-767.329893600	-5.31	-155.90	0.564
ACA	H (3,5-t-Bu2)	0.00	-0.23827	-905.3869509	-945.897790900	H (3,5-t-Bu2)_H	-906.0195331	-945.853220600	-6.48	-117.02	0.726

UBPE0/6-31++G(d,p)

	Products	Hammett and Modified Swain-Lupton Constance	SOMO (a.u.)	HF	+CH4	Reactants	HF	+CH3	SOMO (eV)	RSE (kJ/mol)	spin_d of radical centre carbon
	CH4			-40.4637934	–	CH3	-39.7885723	–			
ABF	NO2	0.78	-0.23202	-1207.055532	-1247.519324900	NO2_H	-1207.676124	-1247.464696600	-6.31	-143.43	0.536
	CN	0.66	-0.22833	-1094.88881	-1135.352603300	CN_H	-1095.509266	-1135.297838300	-6.21	-143.79	0.532
	COMe	0.50	-0.21992	-1155.2317	-1195.695493200	COMe_H	-1155.851339	-1195.639911400	-5.98	-145.93	0.516
	COOMe	0.45	-0.21999	-1230.395222	-1270.859015200	COOMe_H	-1231.015406	-1270.803978700	-5.99	-144.50	0.535
	H	0.00	-0.21334	-1002.748894	-1043.212686900	H_H	-1003.369203	-1043.157775500	-5.81	-144.17	0.560
	Me	-0.17	-0.20876	-1042.021814	-1082.485607600	Me_H	-1042.641345	-1082.429917600	-5.68	-146.21	0.534
	OMe	-0.27	-0.20194	-1117.155649	-1157.619442800	OMe_H	-1117.77364	-1157.562212400	-5.50	-150.26	0.505
	NMe2	-0.83	-0.18605	-1136.572742	-1177.036535800	NMe2_H	-1137.188265	-1176.976837400	-5.06	-156.74	0.465
ABT	NO2	0.78	-0.2339	-1215.790198	-1256.253991400	NO2_H	-1216.411443	-1256.200014800	-6.36	-141.72	0.480
	CN	0.66	-0.22954	-1103.623673	-1144.087466700	CN_H	-1104.244577	-1144.033149300	-6.25	-142.61	0.475
	COMe	0.50	-0.22236	-1163.969552	-1204.433345700	COMe_H	-1164.590549	-1204.379120800	-6.05	-142.37	0.481
	COOMe	0.45	-0.22068	-1239.129968	-1279.593761000	COOMe_H	-1239.750697	-1279.539269000	-6.01	-143.07	0.481
	H	0.00	-0.21331	-1011.483704	-1051.947497000	H_H	-1012.104361	-1051.892933400	-5.80	-143.26	0.489
	Me	-0.17	-0.2082	-1050.756771	-1091.220564100	Me_H	-1051.376606	-1091.165178100	-5.67	-145.42	0.472
	OMe	-0.27	-0.20141	-1125.890305	-1166.354098500	OMe_H	-1126.509016	-1166.297588000	-5.48	-148.37	0.462
	NMe2	-0.83	-0.18427	-1145.30811	-1185.771903400	NMe2_H	-1145.923629	-1185.712200800	-5.01	-156.75	0.426
AI	NO2	0.78	-0.22683	-912.3317143	-952.795507700	NO2_H	-912.9549391	-952.743511400	-6.17	-136.52	0.582
	CN	0.66	-0.22306	-800.1649039	-840.628697300	CN_H	-800.7879597	-840.576532000	-6.07	-136.96	0.580
	COMe	0.50	-0.21624	-860.5104352	-900.974228600	COMe_H	-861.1335222	-900.922094500	-5.88	-136.88	0.584
	COOMe	0.45	-0.21461	-935.6705235	-976.134316900	COOMe_H	-936.2935985	-976.082170800	-5.84	-136.91	0.584
	H	0.00	-0.2073	-708.0241772	-748.487970600	H_H	-708.6470185	-748.435590800	-5.64	-137.52	0.624
	Me	-0.17	-0.20277	-747.2969008	-787.760694200	Me_H	-747.919033	-787.707605300	-5.52	-139.39	0.579
	OMe	-0.27	-0.1961	-822.4306408	-862.894434200	OMe_H	-823.0512616	-862.839833900	-5.34	-143.35	0.567
	NMe2	-0.83	-0.18026	-841.8472842	-882.311077600	NMe2_H	-842.4655417	-882.254114000	-4.91	-149.56	0.532
ADCM	NO2	0.78	-0.28355	-659.1908638	-699.654657200	NO2_H	-659.8111065	-699.599678800	-7.72	-144.35	0.694
	CN	0.66	-0.27608	-547.0256302	-587.489423600	CN_H	-547.6448889	-587.433461200	-7.51	-146.93	0.664
	COMe	0.50	-0.26707	-607.3738276	-647.837621000	COMe_H	-607.9929017	-647.781474000	-7.27	-147.41	0.668
	COOMe	0.45	-0.26533	-682.5340482	-722.997841600	COOMe_H	-683.1529433	-722.941515600	-7.22	-147.88	0.670
	H	0.00	-0.25755	-454.8896889	-495.353482300	H_H	-455.5084248	-495.296997100	-7.01	-148.30	0.705
	Me	-0.17	-0.24858	-494.1637308	-534.627524200	Me_H	-494.7812583	-534.569830600	-6.76	-151.47	0.643
	OMe	-0.27	-0.23613	-569.2984189	-609.762212300	OMe_H	-569.9137803	-609.702352600	-6.43	-157.16	0.622
	NMe2	-0.83	-0.21239	-588.7190033	-629.182796700	NMe2_H	-589.3300053	-629.118577600	-5.78	-168.61	0.551
ACA	NO2	0.78	-0.27128	-794.6929873	-835.156780700	NO2_H	-795.3258899	-835.114462200	-7.38	-111.11	0.714
	CN	0.66	-0.2641	-682.5274152	-722.991208600	CN_H	-683.1593988	-722.947971100	-7.19	-113.52	0.696
	COMe	0.50	-0.25532	-742.874933	-783.338726400	COMe_H	-743.505559	-783.294131300	-6.95	-117.08	0.693
	COOMe	0.45	-0.25361	-818.0346567	-858.498450100	COOMe_H	-818.6653044	-858.453876700	-6.90	-117.03	0.692
	H	0.00	-0.24526	-590.3897794	-630.853572800	H_H	-591.0209405	-630.809512800	-6.67	-115.68	0.786
	Me	-0.17	-0.23684	-629.6636767	-670.127470100	Me_H	-630.2925552	-670.081127500	-6.44	-121.67	0.669
	OMe	-0.27	-0.22505	-704.7985263	-745.262319700	OMe_H	-705.4248965	-745.213468800	-6.12	-128.26	0.631
	NMe2	-0.83	-0.20262	-724.2181995	-764.681992900	NMe2_H	-724.8403164	-764.628888700	-5.51	-139.43	0.579
AM	NO2	0.78	-0.25352	-930.1935817	-970.657375100	NO2_H	-930.8327299	-970.621302200	-6.90	-94.71	0.723
	CN	0.66	-0.24654	-818.0275765	-858.491369900	CN_H	-818.6658742	-858.454446500	-6.71	-96.94	0.701
	COMe	0.50	-0.23786	-878.37398	-918.837773400	COMe_H	-879.0120072	-918.800579500	-6.47	-97.65	0.717
	COOMe	0.45	-0.2362	-953.5336733	-993.997466700	COOMe_H	-954.1720202	-993.960592500	-6.43	-96.81	0.719
	H	0.00	-0.22699	-725.8877368	-766.351530200	H_H	-726.5257656	-766.314337900	-6.18	-97.65	0.704
	Me	-0.17	-0.21914	-765.1612705	-805.625063900	Me_H	-765.7979146	-805.586486900	-5.96	-101.28	0.676
	OMe	-0.27	-0.2081	-840.2958156	-880.759609000	OMe_H	-840.930364	-880.718936300	-5.66	-106.79	0.634
	NMe2	-0.83	-0.1869	-859.7142814	-900.178074800	NMe2_H	-860.3448791	-900.133451400	-5.09	-117.16	0.564
DAAN	NO2	0.78	-0.23609	-797.8582498	-838.322043200	NO2_H	-798.4816281	-838.270200400	-6.42	-136.11	0.637
	CN	0.66	-0.23168	-685.6917119	-726.155505300	CN_H	-686.314956	-726.103528300	-6.30	-136.47	0.641
	COMe	0.50	-0.22391	-746.0387578	-786.502551200	COMe_H	-746.6620143	-786.450586600	-6.09	-136.43	0.637
	COOMe	0.45	-0.22226	-821.1986371	-861.662430500	COOMe_H	-821.821714	-861.610286300	-6.05	-136.90	0.641
	H	0.00	-0.21467	-593.5524783	-634.016271700	H_H	-594.1759913	-633.964563600	-5.84	-135.76	0.620
	Me	-0.17	-0.20942	-632.825388	-673.289181400	Me_H	-633.4483328	-673.236905100	-5.70	-137.25	0.642
	OMe	-0.27	-0.20203	-707.9587738	-748.422567200	OMe_H	-708.5806353	-748.369207600	-5.50	-140.10	0.642
	NMe2	-0.83	-0.18488	-727.3759205	-767.839713900	NMe2_H	-727.9956654	-767.784237700	-5.03	-145.65	0.604
CF	NO2	0.78	-0.24881	-796.6766491	-837.140442500	NO2_H	-797.2972211	-837.085793400	-6.77	-143.48	0.635
	CN	0.66	-0.24441	-684.5100758	-724.973869200	CN_H	-685.1304309	-724.919003200	-6.65	-144.05	0.626
	COMe	0.50	-0.23553	-744.8573677	-785.321161100	COMe_H	-745.4774407	-785.266013000	-6.41	-144.79	0.620
	COOMe	0.45	-0.2343	-820.0171942	-860.480987600	COOMe_H	-820.6375524	-860.426124700	-6.38	-144.04	0.627
	H	0.00	-0.22745	-592.3710848	-632.834878200	H_H	-592.9918064	-632.780378700	-6.19	-143.09	0.634
	Me	-0.17	-0.2218	-631.6438175	-672.107610900	Me_H	-632.2638973	-672.052469600	-6.04	-144.77	0.633
	OMe	-0.27	-0.21266	-706.777641	-747.241434400	OMe_H	-707.3961251	-747.184697400	-5.79	-148.96	0.603
	NMe2	-0.83	-0.19423	-726.1953035	-766.659096900	NMe2_H	-726.8108887	-766.599461000	-5.29	-156.57	0.556
ACA	H (3,5-t-Bu2)	0.00	-0.23742	-904.5392235	-945.003016900	H (3,5-t-Bu2)_H	-905.1694815	-944.958053800	-6.46	-118.05	0.719

UwB97XD/6-31++G(d,p)

	Products	Hammett and Modified Swain-Lupton Constance	SOMO (a.u.)	HF	+CH4	Reactants	HF	+CH3	SOMO (eV)	RSE (kJ/mol)	spin_d of radical centre carbon
	CH4			-40.5083773	–	CH3	-39.8278672	–			
ABF	NO2	0.78	-0.29059	-1208.05176	-1248.560137400	NO2_H	-1208.682032	-1248.509899200	-7.91	-131.90	0.540
	CN	0.66	-0.28711	-1095.816636	-1136.325013600	CN_H	-1096.446759	-1136.274626000	-7.81	-132.29	0.544
	COMe	0.50	-0.27844	-1156.212558	-1196.720935400	COMe_H	-1156.841828	-1196.669695100	-7.58	-134.53	0.531
	COOMe	0.45	-0.27872	-1231.428013	-1271.936390100	COOMe_H	-1232.057782	-1271.885648800	-7.58	-133.22	0.547
	H	0.00	-0.27271	-1003.608549	-1044.116926600	H_H	-1004.23828	-1044.066147500	-7.42	-133.32	0.560
	Me	-0.17	-0.26809	-1042.918823	-1083.427200000	Me_H	-1043.54794	-1083.375807200	-7.30	-134.93	0.537
	OMe	-0.27	-0.26139	-1118.104877	-1158.613253800	OMe_H	-1118.732731	-1158.560598200	-7.11	-138.25	0.521
	NMe2	-0.83	-0.24449	-1137.550436	-1178.058813100	NMe2_H	-1138.176526	-1178.004392900	-6.65	-142.88	0.494
ABT	NO2	0.78	-0.29255	-1216.554888	-1257.063265000	NO2_H	-1217.185314	-1257.013181000	-7.96	-131.50	0.492
	CN	0.66	-0.28834	-1104.319982	-1144.828359200	CN_H	-1104.95005	-1144.777917100	-7.85	-132.44	0.489
	COMe	0.50	-0.28087	-1164.718775	-1205.227152700	COMe_H	-1165.348966	-1205.176832700	-7.64	-132.12	0.493
	COOMe	0.45	-0.27935	-1239.931181	-1280.439558400	COOMe_H	-1240.561067	-1280.388934600	-7.60	-132.91	0.492
	H	0.00	-0.2726	-1012.111816	-1052.620192800	H_H	-1012.741476	-1052.569343600	-7.42	-133.50	0.490
	Me	-0.17	-0.26737	-1051.42225	-1091.930627300	Me_H	-1052.051179	-1091.879045900	-7.28	-135.43	0.481
	OMe	-0.27	-0.26048	-1126.608048	-1167.116425300	OMe_H	-1127.236078	-1167.063944900	-7.09	-137.79	0.470
	NMe2	-0.83	-0.24206	-1146.054377	-1186.562753800	NMe2_H	-1146.679776	-1186.507643600	-6.59	-144.69	0.439
AI	NO2	0.78	-0.28448	-913.04734	-953.555717300	NO2_H	-913.6799281	-953.507795300	-7.74	-125.82	0.583
	CN	0.66	-0.28102	-800.8121418	-841.320519100	CN_H	-801.4445425	-841.272409700	-7.65	-126.31	0.582
	COMe	0.50	-0.27401	-861.2105876	-901.718964900	COMe_H	-861.8430139	-901.670881100	-7.46	-126.24	0.575
	COOMe	0.45	-0.27253	-936.4226836	-976.931060900	COOMe_H	-937.0550507	-976.882917900	-7.42	-126.40	0.576
	H	0.00	-0.26592	-708.6032152	-749.111592500	H_H	-709.2352039	-749.063071100	-7.24	-127.39	0.601
	Me	-0.17	-0.26142	-747.9133138	-788.421691100	Me_H	-748.5447242	-788.372591400	-7.11	-128.91	0.582
	OMe	-0.27	-0.25497	-823.0992584	-863.607635700	OMe_H	-823.7294668	-863.557334000	-6.94	-132.07	0.571
	NMe2	-0.83	-0.23855	-842.544363	-883.052740300	NMe2_H	-843.1729001	-883.000767300	-6.49	-136.46	0.539
ADCM	NO2	0.78	-0.3439	-659.6956786	-700.204055900	NO2_H	-660.3244229	-700.152290100	-9.36	-135.91	0.675
	CN	0.66	-0.33616	-547.4618924	-587.970269700	CN_H	-548.0897794	-587.917646600	-9.15	-138.16	0.635
	COMe	0.50	-0.32687	-607.8631051	-648.371482400	COMe_H	-608.4908368	-648.318704000	-8.89	-138.57	0.653
	COOMe	0.45	-0.32533	-683.075315	-723.583692300	COOMe_H	-683.702822	-723.530689200	-8.85	-139.16	0.653
	H	0.00	-0.31808	-455.2578012	-495.766178500	H_H	-455.8849887	-495.712855900	-8.66	-140.00	0.649
	Me	-0.17	-0.30892	-494.5691746	-535.077551900	Me_H	-495.1952876	-535.023154800	-8.41	-142.82	0.607
	OMe	-0.27	-0.2962	-569.7560851	-610.264462400	OMe_H	-570.3802418	-610.208109000	-8.06	-147.96	0.606
	NMe2	-0.83	-0.27086	-589.2051616	-629.713538900	NMe2_H	-589.8254889	-629.653356100	-7.37	-158.01	0.544
ACA	NO2	0.78	-0.33142	-795.3029251	-835.811302400	NO2_H	-795.9443564	-835.772223600	-9.02	-102.60	0.695
	CN	0.66	-0.32395	-683.0687995	-723.577176800	CN_H	-683.7094522	-723.537319400	-8.82	-104.65	0.678
	COMe	0.50	-0.3149	-743.4693182	-783.977695500	COMe_H	-744.1086024	-783.936469600	-8.57	-108.24	0.675
	COOMe	0.45	-0.31341	-818.6810172	-859.189394500	COOMe_H	-819.3202841	-859.148151300	-8.53	-108.28	0.672
	H	0.00	-0.30565	-590.8629815	-631.371358800	H_H	-591.5026109	-631.330478100	-8.32	-107.33	0.717
	Me	-0.17	-0.29699	-630.1742207	-670.682598000	Me_H	-630.8117222	-670.639589400	-8.08	-112.92	0.648
	OMe	-0.27	-0.28494	-705.3613135	-745.869690800	OMe_H	-705.9965058	-745.824373000	-7.75	-118.98	0.612
	NMe2	-0.83	-0.26087	-724.809478	-765.317855300	NMe2_H	-725.4409963	-765.268863500	-7.10	-128.63	0.562
AM	NO2	0.78	-0.31334	-930.9090693	-971.417446600	NO2_H	-931.5564188	-971.384286000	-8.53	-87.06	0.692
	CN	0.66	-0.30607	-818.6745547	-859.182932000	CN_H	-819.321151	-859.149018200	-8.33	-89.04	0.675
	COMe	0.50	-0.29721	-879.0736473	-919.582024600	COMe_H	-879.7202093	-919.548076500	-8.09	-89.13	0.679
	COOMe	0.45	-0.29571	-954.2857014	-994.794078700	COOMe_H	-954.9321992	-994.760066400	-8.05	-89.30	0.683
	H	0.00	-0.28725	-726.4665996	-766.974976900	H_H	-727.1126806	-766.940547800	-7.82	-90.39	0.666
	Me	-0.17	-0.27908	-765.7774658	-806.285843100	Me_H	-766.422326	-806.250193200	-7.59	-93.60	0.648
	OMe	-0.27	-0.26773	-840.9642567	-881.472634000	OMe_H	-841.6072778	-881.435145000	-7.29	-98.43	0.611
	NMe2	-0.83	-0.24477	-860.4111608	-900.919538100	NMe2_H	-861.0509126	-900.878779800	-6.66	-107.01	0.547
DAAN	NO2	0.78	-0.29438	-798.4883985	-838.996775800	NO2_H	-799.1210451	-838.948912300	-8.01	-125.67	0.643
	CN	0.66	-0.29021	-686.2535005	-726.761877800	CN_H	-686.8859793	-726.713846500	-7.90	-126.11	0.649
	COMe	0.50	-0.28217	-746.6534577	-787.161835000	COMe_H	-747.2859289	-787.113796100	-7.68	-126.13	0.647
	COOMe	0.45	-0.28073	-821.8653621	-862.373739400	COOMe_H	-822.4975887	-862.325455900	-7.64	-126.77	0.643
	H	0.00	-0.27384	-594.0461023	-634.554479600	H_H	-594.6785291	-634.506396300	-7.45	-126.24	0.611
	Me	-0.17	-0.26856	-633.3563947	-673.864772000	Me_H	-633.9883544	-673.816221600	-7.31	-127.47	0.648
	OMe	-0.27	-0.26126	-708.5421766	-749.050553900	OMe_H	-709.1731075	-749.000974700	-7.11	-130.17	0.597
	NMe2	-0.83	-0.24317	-727.9879128	-768.496290100	NMe2_H	-728.617223	-768.445090200	-6.62	-134.43	0.619
CF	NO2	0.78	-0.30657	-797.2948491	-837.803226400	NO2_H	-797.9245491	-837.752416300	-8.34	-133.40	0.630
	CN	0.66	-0.30226	-685.059886	-725.568263300	CN_H	-685.6893631	-725.517230300	-8.22	-133.99	0.631
	COMe	0.50	-0.29318	-745.4600797	-785.968457000	COMe_H	-746.0893052	-785.917172400	-7.98	-134.65	0.623
	COOMe	0.45	-0.29205	-820.6719163	-861.180293600	COOMe_H	-821.3013466	-861.129213800	-7.95	-134.11	0.624
	H	0.00	-0.28567	-592.8525936	-633.360970900	H_H	-593.4822034	-633.310070600	-7.77	-133.64	0.631
	Me	-0.17	-0.27998	-632.1627623	-672.671139600	Me_H	-632.7918428	-672.619710000	-7.62	-135.03	0.632
	OMe	-0.27	-0.27086	-707.3487847	-747.857162000	OMe_H	-707.9765516	-747.804418800	-7.37	-138.48	0.606
	NMe2	-0.83	-0.25127	-726.79502	-767.303397300	NMe2_H	-727.4205181	-767.248385300	-6.84	-144.43	0.562
ACA	H (3,5-t-Bu2)	0.00	-0.2983	-905.3222044	-945.830581700	H (3,5-t-Bu2)_H	-905.9609411	-945.788808300	-8.12	-109.68	0.683

UM06-2X/6-31++G(d,p)

	Products	Hammett and Modified Swain-Lupton Constance	SOMO (a.u.)	HF	+CH4	Reactants	HF	+CH3	SOMO (eV)	RSE (kJ/mol)	spin_d of radical centre carbon
	CH4			-40.4879458	–	CH3	-39.8119135	–			
ABF	NO2	0.78	-0.26257	-1207.906606	-1248.394552200	NO2_H	-1208.533091	-1248.345004300	-7.14	-130.09	0.515
	CN	0.66	-0.25882	-1095.689232	-1136.177177500	CN_H	-1096.315568	-1136.127481600	-7.04	-130.48	0.499
	COMe	0.50	-0.25004	-1156.062338	-1196.550284000	COMe_H	-1156.687657	-1196.499570200	-6.80	-133.15	0.472
	COOMe	0.45	-0.25071	-1231.276882	-1271.764827600	COOMe_H	-1231.902828	-1271.714741500	-6.82	-131.50	0.501
	H	0.00	-0.24458	-1003.473529	-1043.961475200	H_H	-1004.099492	-1043.911405400	-6.66	-131.46	0.533
	Me	-0.17	-0.2402	-1042.773319	-1083.261264800	Me_H	-1043.39862	-1083.210533900	-6.54	-133.19	0.518
	OMe	-0.27	-0.23406	-1117.958644	-1158.446590200	OMe_H	-1118.582462	-1158.394375200	-6.37	-137.09	0.488
	NMe2	-0.83	-0.21747	-1137.391626	-1177.879571800	NMe2_H	-1138.013112	-1177.825025600	-5.92	-143.21	0.440
ABT	NO2	0.78	-0.26469	-1216.480415	-1256.968360300	NO2_H	-1217.106464	-1256.918377000	-7.20	-131.23	0.479
	CN	0.66	-0.26023	-1104.263233	-1144.751178900	CN_H	-1104.888931	-1144.700844400	-7.08	-132.15	0.479
	COMe	0.50	-0.25285	-1164.639213	-1205.127159000	COMe_H	-1165.264975	-1205.076888200	-6.88	-131.99	0.488
	COOMe	0.45	-0.25161	-1239.850698	-1280.338643300	COOMe_H	-1240.476204	-1280.288117600	-6.85	-132.66	0.494
	H	0.00	-0.2448	-1012.047362	-1052.535307600	H_H	-1012.672713	-1052.484626500	-6.66	-133.06	0.487
	Me	-0.17	-0.23985	-1051.34728	-1091.835225900	Me_H	-1051.971899	-1091.783812900	-6.53	-134.98	0.477
	OMe	-0.27	-0.23371	-1126.53229	-1167.020235300	OMe_H	-1127.155845	-1166.967758500	-6.36	-137.78	0.461
	NMe2	-0.83	-0.21575	-1145.965941	-1186.453886700	NMe2_H	-1146.586381	-1186.398294400	-5.87	-145.96	0.434
AI	NO2	0.78	-0.25627	-912.971081	-953.459026800	NO2_H	-913.5996781	-953.411591600	-6.97	-124.54	0.542
	CN	0.66	-0.25251	-800.7535995	-841.241545300	CN_H	-801.3820218	-841.193935300	-6.87	-125.00	0.540
	COMe	0.50	-0.24556	-861.1292429	-901.617188700	COMe_H	-861.7576192	-901.569532700	-6.68	-125.12	0.539
	COOMe	0.45	-0.24432	-936.3404338	-976.828379600	COOMe_H	-936.9687856	-976.780699100	-6.65	-125.19	0.540
	H	0.00	-0.23759	-708.5369951	-749.024940900	H_H	-709.165068	-748.976981500	-6.47	-125.92	0.581
	Me	-0.17	-0.23332	-747.8366004	-788.324546200	Me_H	-748.4640631	-788.275976600	-6.35	-127.52	0.550
	OMe	-0.27	-0.22742	-823.0217731	-863.509718900	OMe_H	-823.6478607	-863.459774200	-6.19	-131.13	0.538
	NMe2	-0.83	-0.21117	-842.4542255	-882.942171300	NMe2_H	-843.0781712	-882.890084700	-5.75	-136.75	0.504
ADCM	NO2	0.78	-0.31406	-659.6639093	-700.151855100	NO2_H	-660.2919139	-700.103827400	-8.55	-126.10	0.662
	CN	0.66	-0.30615	-547.4479841	-587.935929900	CN_H	-548.0750654	-587.886978900	-8.33	-128.52	0.670
	COMe	0.50	-0.29672	-607.8263509	-648.314296700	COMe_H	-608.452928	-648.264841500	-8.07	-129.84	0.631
	COOMe	0.45	-0.29559	-683.0375678	-723.525513600	COOMe_H	-683.6642042	-723.476117700	-8.04	-129.69	0.642
	H	0.00	-0.28795	-455.2360891	-495.724034900	H_H	-455.8618141	-495.673727600	-7.84	-132.08	0.713
	Me	-0.17	-0.27913	-494.5370019	-535.024947700	Me_H	-495.1615598	-534.973473300	-7.60	-135.15	0.654
	OMe	-0.27	-0.26748	-569.7233787	-610.211324500	OMe_H	-570.3455657	-610.157479200	-7.28	-141.37	0.567
	NMe2	-0.83	-0.24244	-589.1602456	-629.648191400	NMe2_H	-589.7776073	-629.589520800	-6.60	-154.04	0.478
ACA	NO2	0.78	-0.30276	-795.2475068	-835.735452600	NO2_H	-795.8868771	-835.698790600	-8.24	-96.26	0.670
	CN	0.66	-0.29504	-683.0312136	-723.519159400	CN_H	-683.6697269	-723.481640400	-8.03	-98.51	0.653
	COMe	0.50	-0.28589	-743.4088934	-783.896839200	COMe_H	-744.0459873	-783.857900800	-7.78	-102.23	0.643
	COOMe	0.45	-0.28481	-818.6196575	-859.107603300	COOMe_H	-819.2567498	-859.068663300	-7.75	-102.24	0.647
	H	0.00	-0.27675	-590.8177092	-631.305655000	H_H	-591.4551749	-631.267088400	-7.53	-101.26	0.751
	Me	-0.17	-0.26839	-630.1184984	-670.606444200	Me_H	-630.7536881	-670.565601600	-7.30	-107.23	0.612
	OMe	-0.27	-0.2573	-705.3050492	-745.792995000	OMe_H	-705.9375129	-745.749426400	-7.00	-114.39	0.570
	NMe2	-0.83	-0.23347	-724.7410005	-765.228946300	NMe2_H	-725.3688407	-765.180754200	-6.35	-126.53	0.492
AM	NO2	0.78	-0.2864	-930.8295619	-971.317507700	NO2_H	-931.4743274	-971.286240900	-7.79	-82.09	0.688
	CN	0.66	-0.27871	-818.6128287	-859.100774500	CN_H	-819.2568013	-859.068714800	-7.58	-84.17	0.671
	COMe	0.50	-0.26976	-878.9892888	-919.477234600	COMe_H	-879.6330411	-919.444954600	-7.34	-84.75	0.683
	COOMe	0.45	-0.26873	-954.2001105	-994.688056300	COOMe_H	-954.8441572	-994.656070700	-7.31	-83.98	0.683
	H	0.00	-0.26007	-726.3972376	-766.885183400	H_H	-727.0408046	-766.852718100	-7.08	-85.24	0.658
	Me	-0.17	-0.2521	-765.6976936	-806.185639400	Me_H	-766.3399212	-806.151834700	-6.86	-88.75	0.628
	OMe	-0.27	-0.24165	-840.8839594	-881.371905200	OMe_H	-841.5239885	-881.335902000	-6.58	-94.53	0.569
	NMe2	-0.83	-0.21876	-860.3187724	-900.806718200	NMe2_H	-860.9544547	-900.766368200	-5.95	-105.94	0.485
DAAN	NO2	0.78	-0.26465	-798.4306588	-838.918604600	NO2_H	-799.0607118	-838.872625300	-7.20	-120.72	0.602
	CN	0.66	-0.26009	-686.2135025	-726.701448300	CN_H	-686.8433934	-726.655306900	-7.08	-121.14	0.609
	COMe	0.50	-0.25211	-746.5906329	-787.078578700	COMe_H	-747.2205097	-787.032423200	-6.86	-121.18	0.591
	COOMe	0.45	-0.25094	-821.8016001	-862.289545900	COOMe_H	-822.4312742	-862.243187700	-6.83	-121.71	0.598
	H	0.00	-0.2438	-593.9983563	-634.486302100	H_H	-594.6283247	-634.440238200	-6.63	-120.94	0.577
	Me	-0.17	-0.23872	-633.2981426	-673.786088400	Me_H	-633.9276222	-673.739535700	-6.50	-122.22	0.608
	OMe	-0.27	-0.23201	-708.4831	-748.971045800	OMe_H	-709.1114555	-748.923369000	-6.31	-125.18	0.599
	NMe2	-0.83	-0.21414	-727.9162478	-768.404193600	NMe2_H	-728.5423792	-768.354292700	-5.83	-131.01	0.542
CF	NO2	0.78	-0.27778	-797.2457818	-837.733727600	NO2_H	-797.872905	-837.684818500	-7.56	-128.41	0.605
	CN	0.66	-0.27316	-685.0285723	-725.516518100	CN_H	-685.6554646	-725.467378100	-7.43	-129.02	0.599
	COMe	0.50	-0.26406	-745.4059146	-785.893860400	COMe_H	-746.0325563	-785.844469800	-7.19	-129.68	0.586
	COOMe	0.45	-0.26326	-820.6168374	-861.104783200	COOMe_H	-821.2437086	-861.055622100	-7.16	-129.07	0.597
	H	0.00	-0.25664	-592.8135767	-633.301522500	H_H	-593.440722	-633.252635500	-6.98	-128.35	0.598
	Me	-0.17	-0.25117	-632.1132224	-672.601168200	Me_H	-632.7398029	-672.551716400	-6.83	-129.84	0.603
	OMe	-0.27	-0.24272	-707.2985403	-747.786486100	OMe_H	-707.9235757	-747.735489200	-6.60	-133.89	0.565
	NMe2	-0.83	-0.22353	-726.7324219	-767.220367700	NMe2_H	-727.3543475	-767.166261000	-6.08	-142.06	0.508
ACA	H (3,5-t-Bu2)	0.00	-0.27022	-905.1942275	-945.682173300	H (3,5-t-Bu2)_H	-905.8308763	-945.642789800	-7.35	-103.40	0.660

ROMP2/6-31++G(d,p)//UB3LYP/6-31++G(d,p)

	Products	Hammett and Modified Swain-Lupton Constance	SOMO (a.u.)	HF	+CH4	Reactants	HF	+CH3	SOMO (eV)	RSE (kJ/mol)	spin_d of radical centre carbon
	CH4			-40.2019604	—	CH3	-39.5625665	—			
ABF	NO2	0.78	-0.29338	-1200.909497	-1241.111457800	NO2_H	-1201.520632	-1241.083198600	-7.98	-74.19	0.571
	CN	0.66	-0.28799	-1089.172176	-1129.374136800	CN_H	-1089.783033	-1129.345599800	-7.84	-74.92	0.572
	COMe	0.50	-0.27833	-1149.213675	-1189.415635400	COMe_H	-1149.824137	-1189.386703000	-7.57	-75.96	0.564
	COOMe	0.45	-0.28055	-1224.092898	-1264.294858700	COOMe_H	-1224.703381	-1264.265947100	-7.63	-75.91	0.582
	H	0.00	-0.27273	-997.4409893	-1037.642949700	H_H	-998.0510161	-1037.613582600	-7.42	-77.10	0.602
	Me	-0.17	-0.26748	-1036.481253	-1076.683213400	Me_H	-1037.090833	-1076.653399600	-7.28	-78.28	0.571
	OMe	-0.27	-0.26181	-1111.326719	-1151.528679000	OMe_H	-1111.935322	-1151.497888200	-7.12	-80.84	0.555
	NMe2	-0.83	-0.24414	-1130.529489	-1170.731449400	NMe2_H	-1131.137373	-1170.699939700	-6.64	-82.73	0.530
ABT	NO2	0.78	-0.30617	-1211.259313	-1251.461273800	NO2_H	-1211.869879	-1251.432445300	-8.33	-75.69	0.625
	CN	0.66	-0.29857	-1099.522079	-1139.724039700	CN_H	-1100.1323	-1139.694866700	-8.12	-76.59	0.621
	COMe	0.50	-0.29057	-1159.566875	-1199.768835200	COMe_H	-1160.177216	-1199.739782100	-7.91	-76.28	0.621
	COOMe	0.45	-0.29043	-1234.442548	-1274.644508400	COOMe_H	-1235.052571	-1274.615137900	-7.90	-77.11	0.620
	H	0.00	-0.28063	-1007.790692	-1047.992652500	H_H	-1008.400124	-1047.962690200	-7.64	-78.67	0.611
	Me	-0.17	-0.27373	-1046.831044	-1087.033004300	Me_H	-1047.440013	-1087.002579900	-7.45	-79.88	0.602
	OMe	-0.27	-0.26724	-1121.676154	-1161.878114000	OMe_H	-1122.284605	-1161.847171600	-7.27	-81.24	0.588
	NMe2	-0.83	-0.24511	-1140.879563	-1181.081523700	NMe2_H	-1141.486466	-1181.049032100	-6.67	-85.31	0.550
AI	NO2	0.78	-0.28637	-907.8332545	-948.035214900	NO2_H	-908.4457337	-948.008300200	-7.79	-70.66	0.591
	CN	0.66	-0.28108	-796.095855	-836.297815400	CN_H	-796.7080122	-836.270578700	-7.65	-71.51	0.593
	COMe	0.50	-0.27398	-856.1403235	-896.342283900	COMe_H	-856.7525147	-896.315081200	-7.46	-71.42	0.596
	COOMe	0.45	-0.27361	-931.0157361	-971.217696500	COOMe_H	-931.6278348	-971.190401300	-7.45	-71.66	0.600
	H	0.00	-0.26518	-704.3638818	-744.565842200	H_H	-704.9751431	-744.537709600	-7.22	-73.86	0.614
	Me	-0.17	-0.26016	-743.4040022	-783.605962600	Me_H	-744.0148311	-783.577397600	-7.08	-75.00	0.592
	OMe	-0.27	-0.25478	-818.2493616	-858.451322000	OMe_H	-818.8593295	-858.421896000	-6.93	-77.26	0.580
	NMe2	-0.83	-0.23763	-837.4517927	-877.653753100	NMe2_H	-838.0610738	-877.623640300	-6.47	-79.06	0.557
ADCM	NO2	0.78	-0.35858	-656.0568358	-696.258796200	NO2_H	-656.6690951	-696.231661600	-9.76	-71.24	0.657
	CN	0.66	-0.34539	-544.3205686	-584.522529000	CN_H	-544.9323127	-584.494879200	-9.40	-72.59	0.647
	COMe	0.50	-0.33575	-604.3681141	-644.570074500	COMe_H	-604.9795306	-644.542097100	-9.14	-73.45	0.641
	COOMe	0.45	-0.33639	-679.2435362	-719.445496600	COOMe_H	-679.8547099	-719.417276400	-9.15	-74.09	0.645
	H	0.00	-0.32563	-452.593889	-492.795849400	H_H	-453.2041768	-492.766743300	-8.86	-76.42	0.632
	Me	-0.17	-0.31503	-491.6351532	-531.837113600	Me_H	-492.2446962	-531.807262700	-8.57	-78.37	0.617
	OMe	-0.27	-0.3044	-566.4812543	-606.683214700	OMe_H	-567.0892935	-606.651860000	-8.28	-82.32	0.592
	NMe2	-0.83	-0.27627	-585.6872449	-625.889205300	NMe2_H	-586.2925474	-625.855113900	-7.52	-89.51	0.537
ACA	NO2	0.78	-0.34785	-790.974443	-831.176403400	NO2_H	-791.5966614	-831.159227900	-9.47	-45.09	0.692
	CN	0.66	-0.3347	-679.2377426	-719.439703000	CN_H	-679.8595775	-719.422144000	-9.11	-46.10	0.682
	COMe	0.50	-0.32524	-739.284482	-779.486442400	COMe_H	-739.9050989	-779.467665400	-8.85	-49.30	0.675
	COOMe	0.45	-0.32594	-814.1593425	-854.361302900	COOMe_H	-814.7800411	-854.342607600	-8.87	-49.08	0.679
	H	0.00	-0.31464	-587.5091388	-627.711099200	H_H	-588.129509	-627.692075500	-8.56	-49.95	0.664
	Me	-0.17	-0.3044	-626.5501651	-666.752125500	Me_H	-627.1690331	-666.731599600	-8.28	-53.89	0.648
	OMe	-0.27	-0.29436	-701.3964069	-741.598367300	OMe_H	-702.0135438	-741.576110300	-8.01	-58.44	0.621
	NMe2	-0.83	-0.26718	-720.6012362	-760.803196600	NMe2_H	-721.216207	-760.778773500	-7.27	-64.12	0.565
AM	NO2	0.78	-0.33284	-925.8921164	-966.094076800	NO2_H	-926.5168835	-966.079450000	-9.06	-38.40	0.744
	CN	0.66	-0.31929	-814.1548432	-854.356803600	CN_H	-814.7794524	-854.342018900	-8.69	-38.82	0.732
	COMe	0.50	-0.30983	-874.1998319	-914.401792300	COMe_H	-874.8244957	-914.387062200	-8.43	-38.67	0.726
	COOMe	0.45	-0.31113	-949.0752878	-989.277248200	COOMe_H	-949.6997427	-989.262309200	-8.47	-39.22	0.738
	H	0.00	-0.29883	-722.4239006	-762.625861000	H_H	-723.0475682	-762.610134700	-8.13	-41.29	0.719
	Me	-0.17	-0.28868	-761.4643328	-801.666293200	Me_H	-762.0874531	-801.650019600	-7.86	-42.73	0.700
	OMe	-0.27	-0.27884	-836.3099639	-876.511924300	OMe_H	-836.9321642	-876.494730700	-7.59	-45.14	0.666
	NMe2	-0.83	-0.25194	-855.5128123	-895.714772700	NMe2_H	-856.1341336	-895.696700100	-6.86	-47.45	0.593
DAAN	NO2	0.78	-0.30224	-793.8986475	-834.100607900	NO2_H	-794.5119239	-834.074490400	-8.22	-68.57	0.666
	CN	0.66	-0.29565	-682.1615572	-722.363517600	CN_H	-682.7745111	-722.337077600	-8.05	-69.42	0.596
	COMe	0.50	-0.28732	-742.207592	-782.409552400	COMe_H	-742.8204775	-782.383044000	-7.82	-69.60	0.667
	COOMe	0.45	-0.2873	-817.0828358	-857.284796200	COOMe_H	-817.6953607	-857.257927200	-7.82	-70.54	0.671
	H	0.00	-0.27838	-590.4312353	-630.633195700	H_H	-591.0433969	-630.605963400	-7.58	-71.50	0.672
	Me	-0.17	-0.27233	-629.4714771	-669.673437500	Me_H	-630.0834154	-669.645981900	-7.41	-72.08	0.664
	OMe	-0.27	-0.26625	-704.3166372	-744.518597600	OMe_H	-704.9278137	-744.490380200	-7.25	-74.08	0.659
	NMe2	-0.83	-0.24675	-723.5196232	-763.721583600	NMe2_H	-724.1300601	-763.692626600	-6.71	-76.03	0.636
CF	NO2	0.78	-0.31074	-792.7463433	-832.948303700	NO2_H	-793.356731	-832.919297500	-8.46	-76.16	0.614
	CN	0.66	-0.3044	-681.0090447	-721.211005100	CN_H	-681.6193371	-721.181903600	-8.28	-76.41	0.615
	COMe	0.50	-0.2947	-741.0553441	-781.257304500	COMe_H	-741.6653076	-781.227874100	-8.02	-77.27	0.615
	COOMe	0.45	-0.29504	-815.93057	-856.132530400	COOMe_H	-816.5406079	-856.103174400	-8.03	-77.07	0.618
	H	0.00	-0.2867	-589.2787973	-629.480757700	H_H	-589.8886763	-629.451242800	-7.80	-77.49	0.618
	Me	-0.17	-0.28036	-628.3187623	-668.520722700	Me_H	-628.9283861	-668.490952600	-7.63	-78.16	0.612
	OMe	-0.27	-0.27256	-703.1639571	-743.365917500	OMe_H	-703.7729554	-743.335521900	-7.42	-79.80	0.596
	NMe2	-0.83	-0.25116	-722.3666054	-762.568565800	NMe2_H	-722.9747752	-762.537341700	-6.83	-81.98	0.559
ACA	H (3,5-t-Bu2)	0.00	-0.30675	-899.802151	-940.004111400	H (3,5-t-Bu2)_H	-900.4223943	-939.984960800	-8.35	-50.28	0.663

7. CoGEF studies

7-1. Details of CoGEF calculation method

CoGEF calculations were performed following Beyer's method[6] using Gaussian 16 program package.[1] The distance between the two methyl groups was constrained and increased by increments of 0.1 Å at B3LYP/6-31G(d) level. In order to obtain the energy profile at higher calculation level, after done the calculation, the one previous structure before reaching the top of energy profile was extracted, and it was optimized at UB3LYP/6-31++G(d,p) levels with bond freezing of two methyl groups. Further CoGEF calculation were performed with increments of 0.05 Å at UB3LYP/6-31++G(d) level. The energy of obtained structures at each mechanophores were normalized by each calculated levels of initial structure 0 kcal/mol. F_{max} values were calculated from the slope by two points structures preceding the abrupt attenuation in energy. Obtained normalized energies (a.u.) were convert to nJ/mol, and the values were divided by the Avogadro constant. Calculated values (nJ) were divided by displacement (Δm) to provided force of nJ/m (nN).

The geometries were given by stick type model (atom color: gray = carbon, red = oxygen, blue = nitrogen, white = hydrogen).

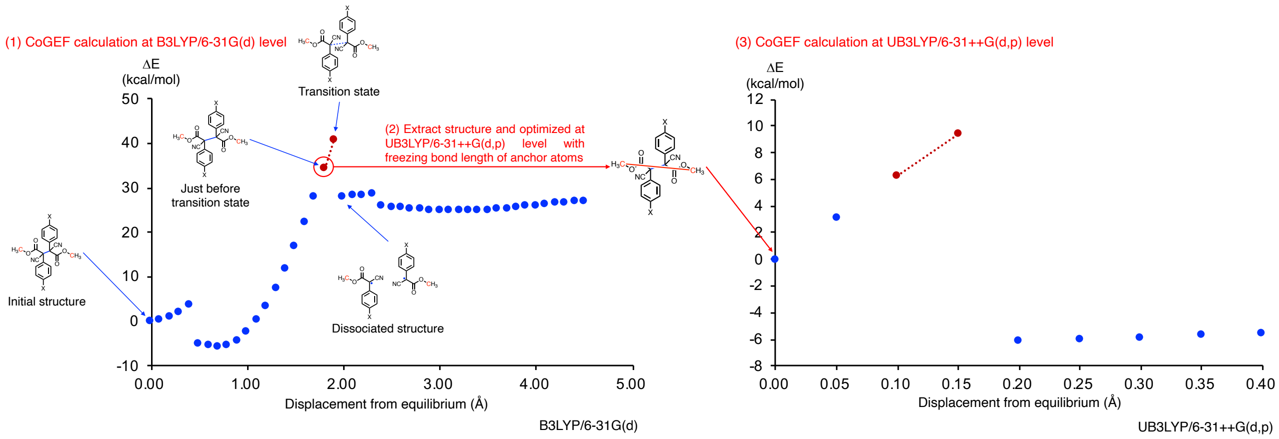

Fig. S37. Illustration of a sequence of CoGEF calculation at UB3LYP/6-31++G(d,p) level.

7-2. Potential energy curves calculated by CoGEF method at B3LYP/6-31G(d) level

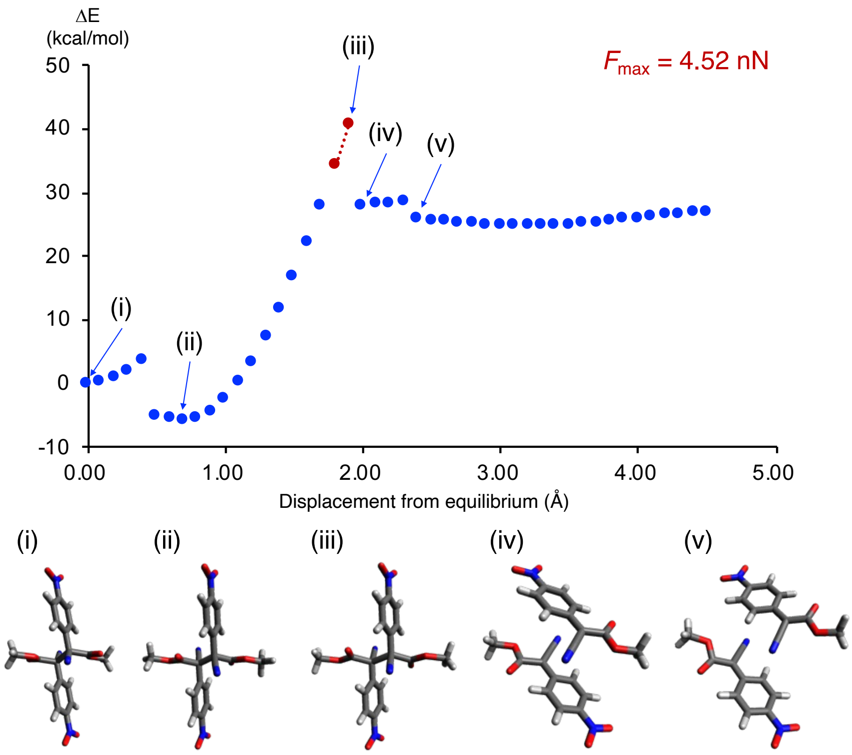

Fig. S38. Potential energy curve of BiACA-NO_2 with corresponding structures at initial state, F_{max} and after central C–C bond dissociation.

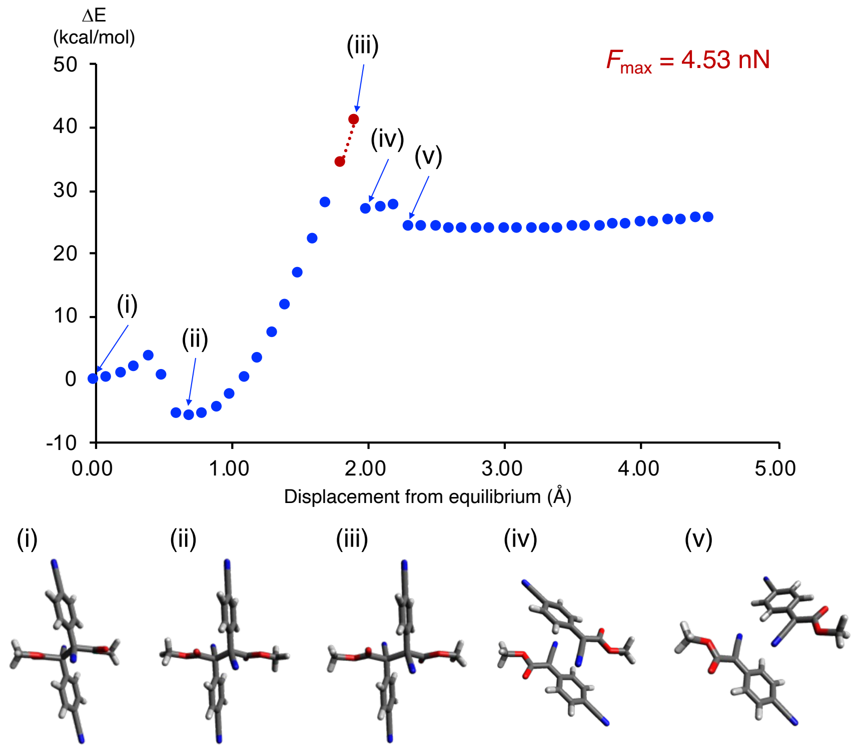

Fig. S39. Potential energy curve of BiACA-CN with corresponding structures at initial state, F_{max} and after central C–C bond dissociation.

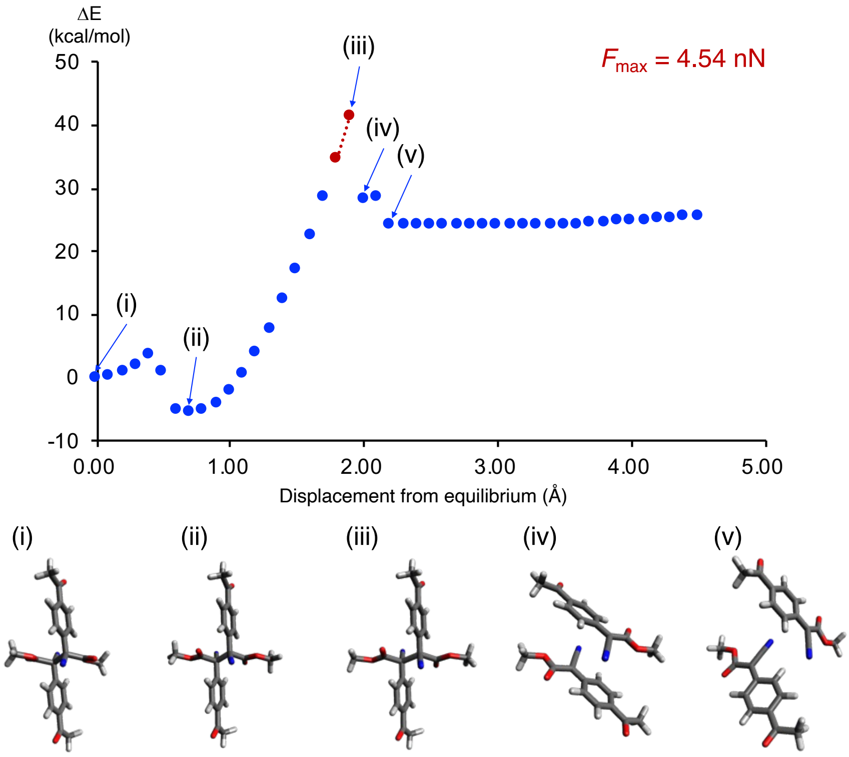

Fig. S40. Potential energy curve of BiACA-COMe with corresponding structures at initial state, F_{max} and after central C–C bond dissociation.

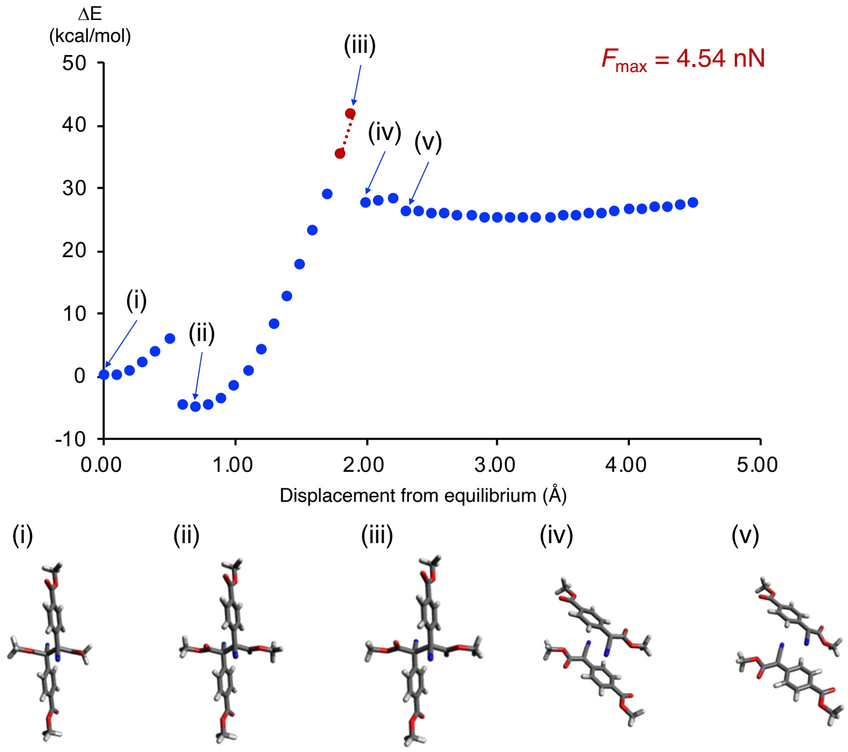

Fig. S41. Potential energy curve of BiACA-COOMe with corresponding structures at initial state, F_{max} and after central C–C bond dissociation.

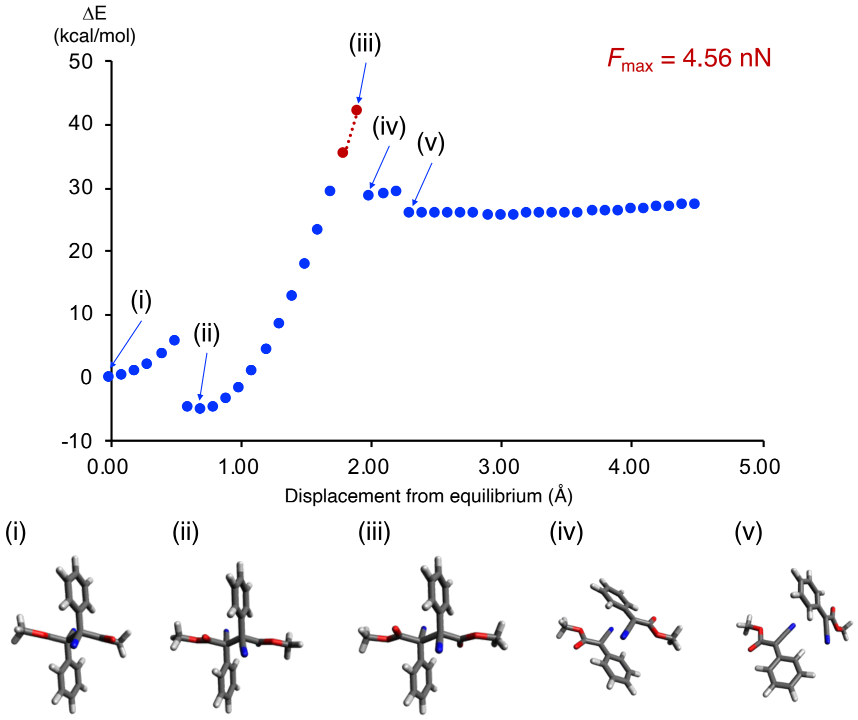

Fig. S42. Potential energy curve of BiACA-H with corresponding structures at initial state, F_{max} and after central C–C bond dissociation.

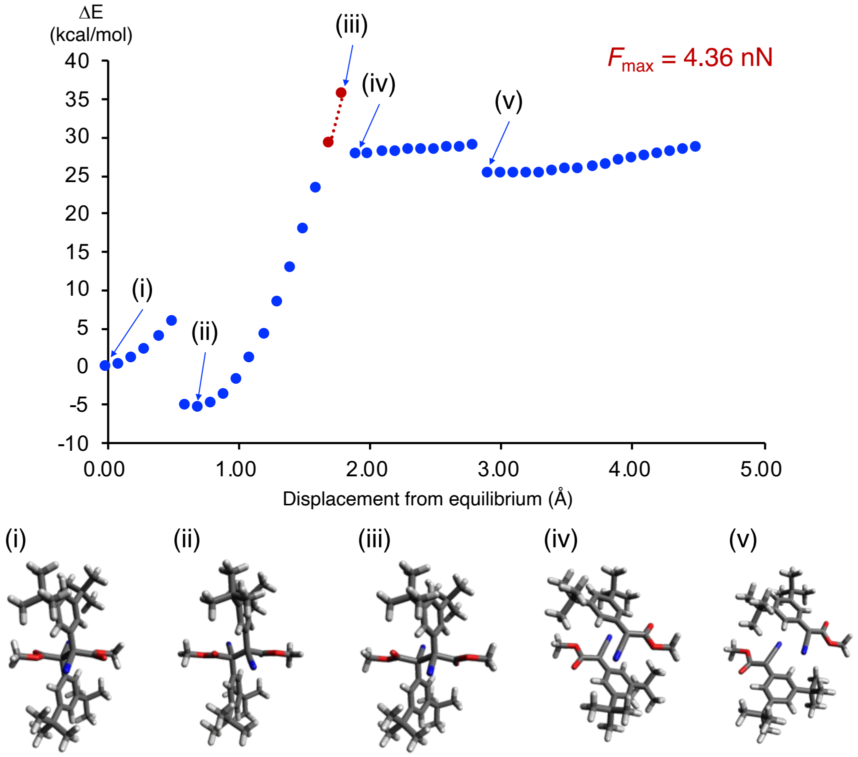

Fig. S43. Potential energy curve of BiACA-H (3,5-*t*-Bu_2) with corresponding structures at initial state, F_{max} and after central C–C bond dissociation.

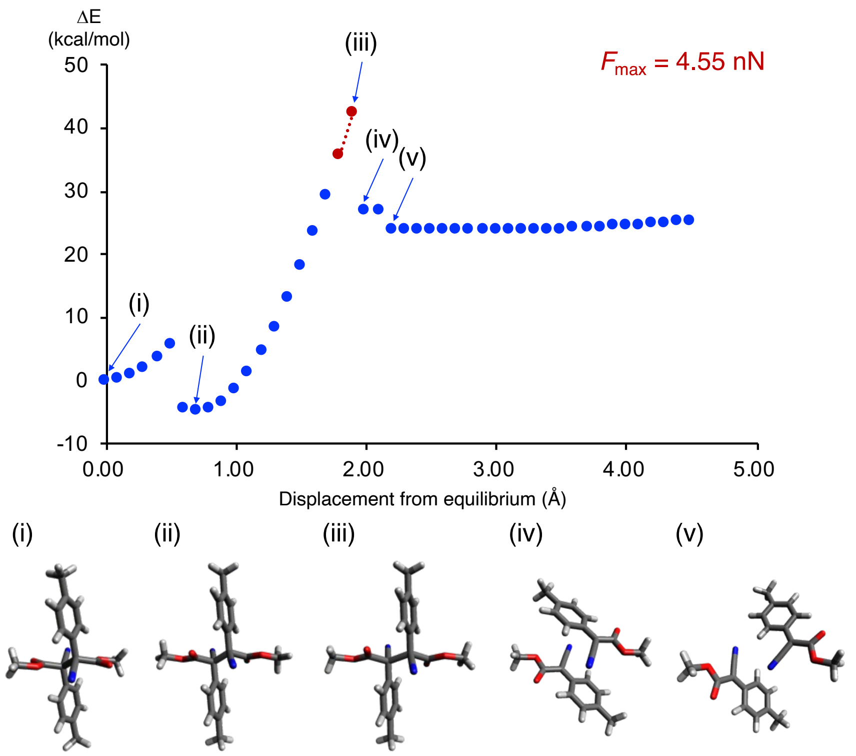

Fig. S44. Potential energy curve of BiACA-Me with corresponding structures at initial state, $F_{\max}$ and after central C–C bond dissociation.

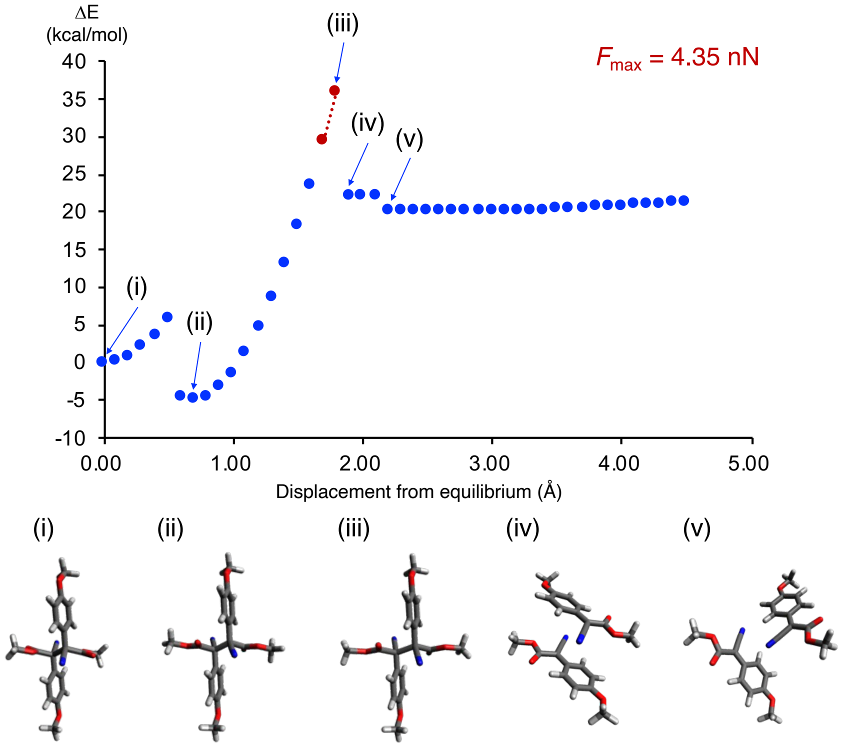

Fig. S45. Potential energy curve of BiACA-OMe with corresponding structures at initial state, $F_{\max}$ and after central C–C bond dissociation.

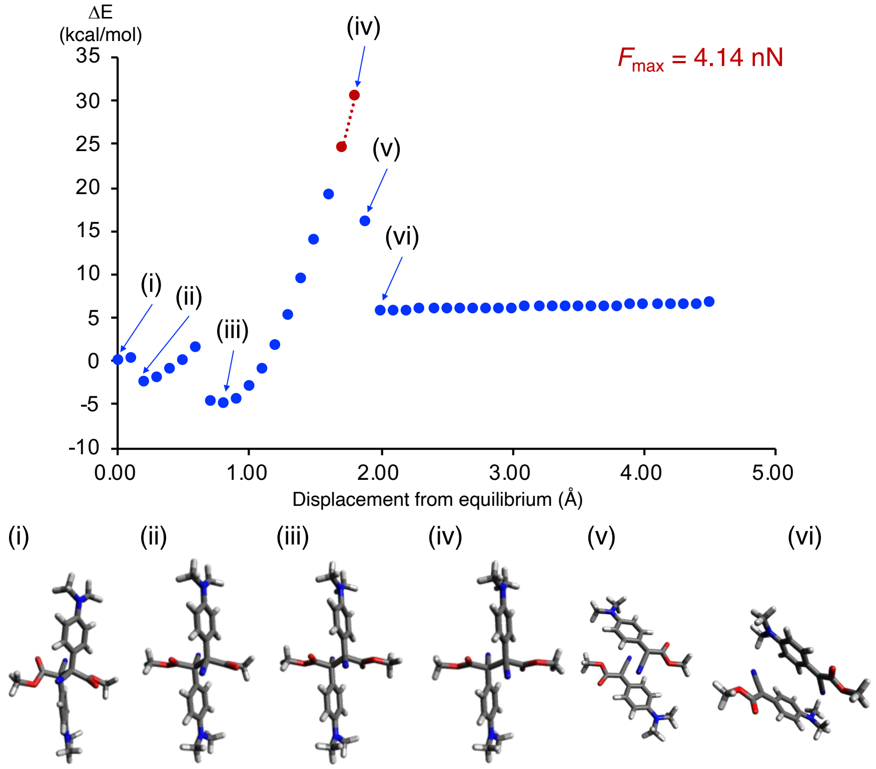

Fig. S46. Potential energy curve of BiACA-NMe_2 with corresponding structures at initial state, F_{max} and after central C–C bond dissociation.

7-3. Potential energy curves calculated by CoGEF method at UB3LYP/6-31G++(d,p) level

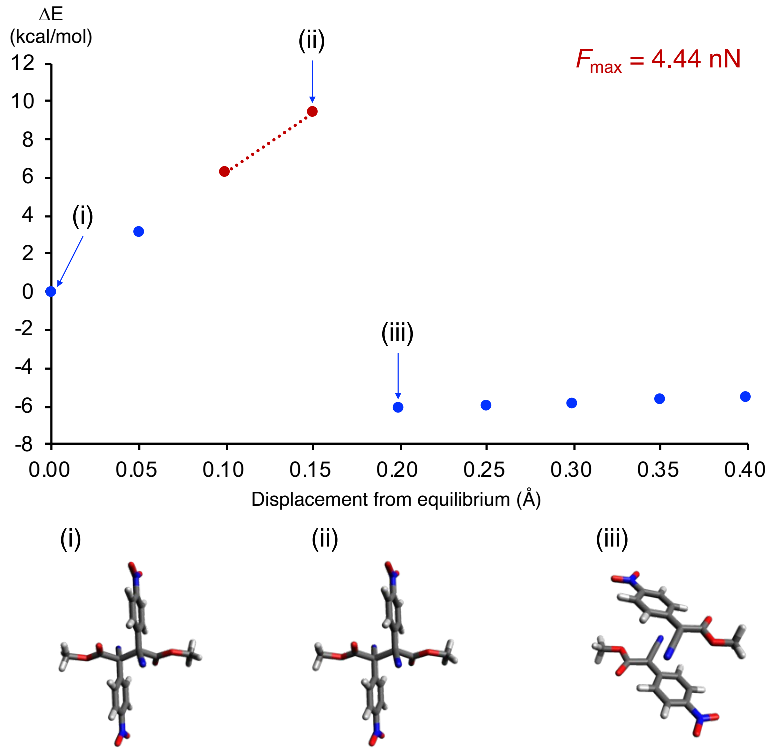

Fig. S47. Potential energy curve of BiACA-NO_2 with corresponding structures at initial state, F_{max} and after central C–C bond dissociation.

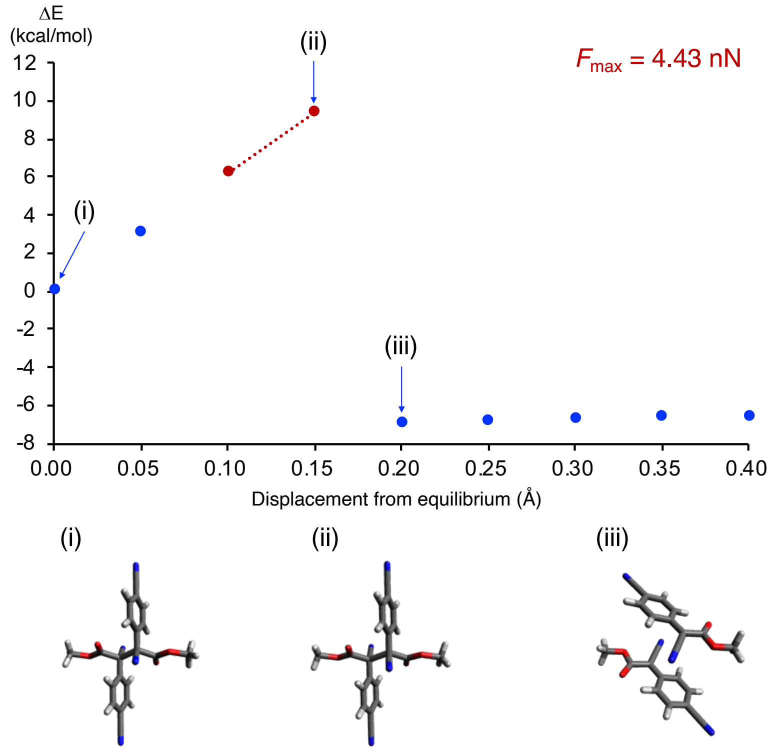

Fig. S48. Potential energy curve of BiACA-CN with corresponding structures at initial state, F_{max} and after central C–C bond dissociation.

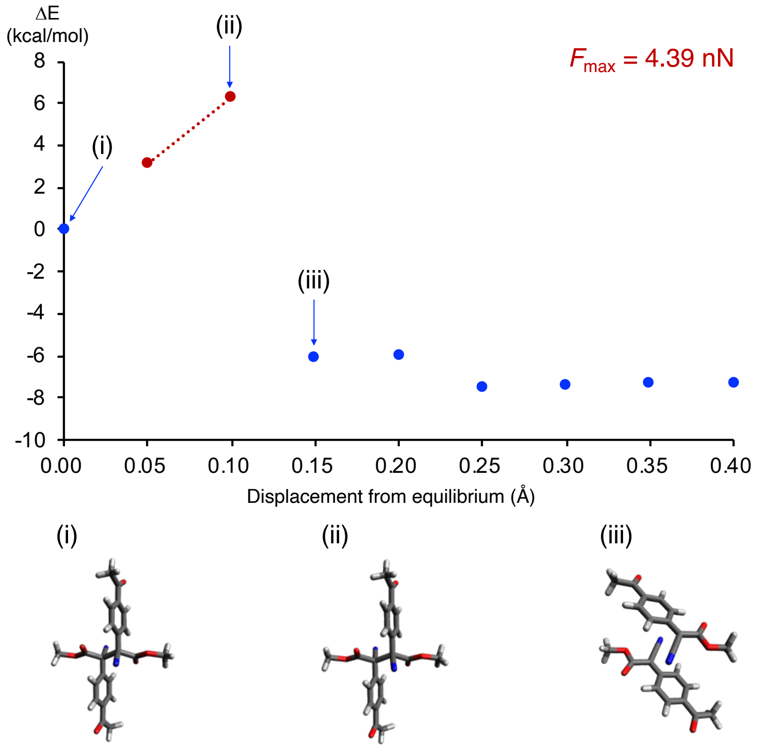

Fig. S49. Potential energy curve of BiACA-COMe with corresponding structures at initial state, F_{max} and after central C–C bond dissociation.

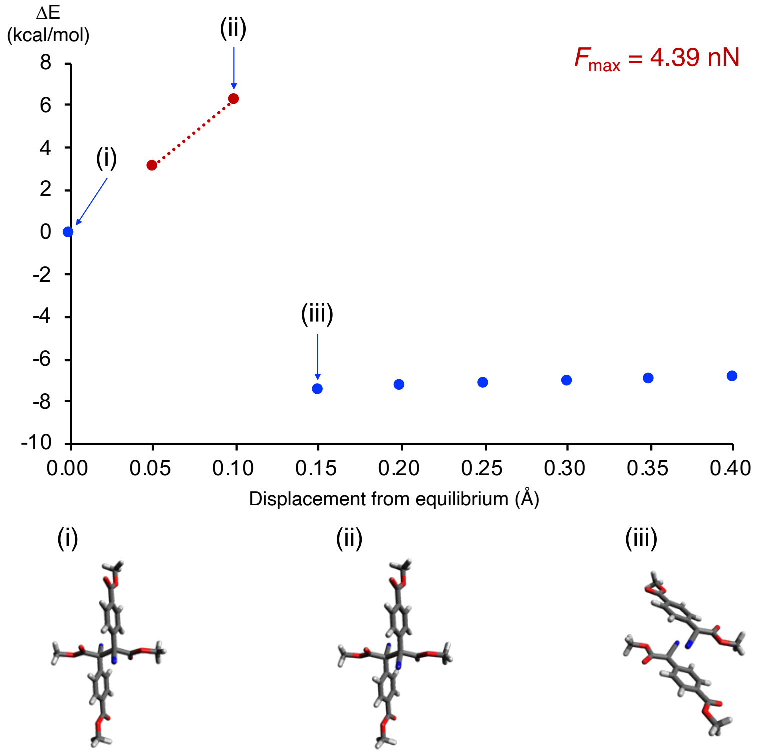

Fig. S50. Potential energy curve of BiACA-COOMe with corresponding structures at initial state, F_{max} and after central C–C bond dissociation.

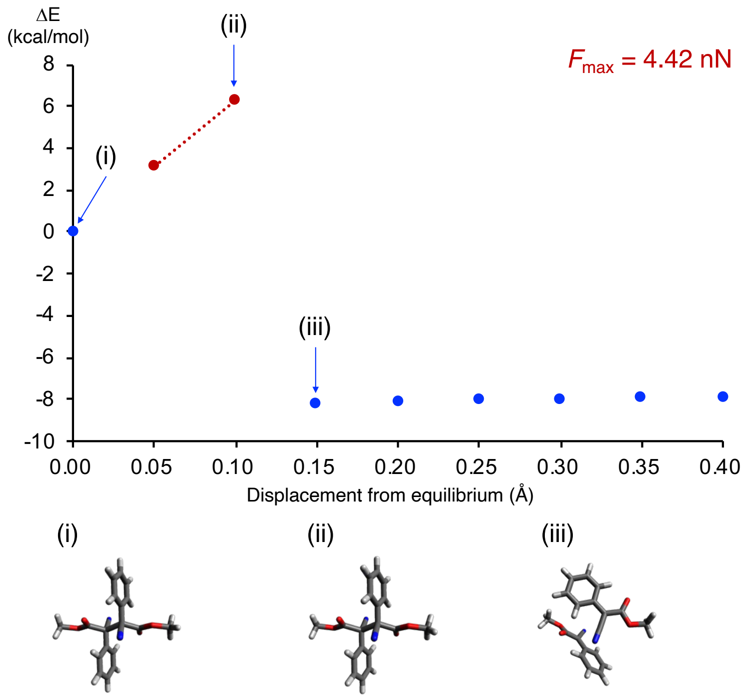

Fig. S51. Potential energy curve of BiACA-H with corresponding structures at initial state, F_{max} and after central C–C bond dissociation.

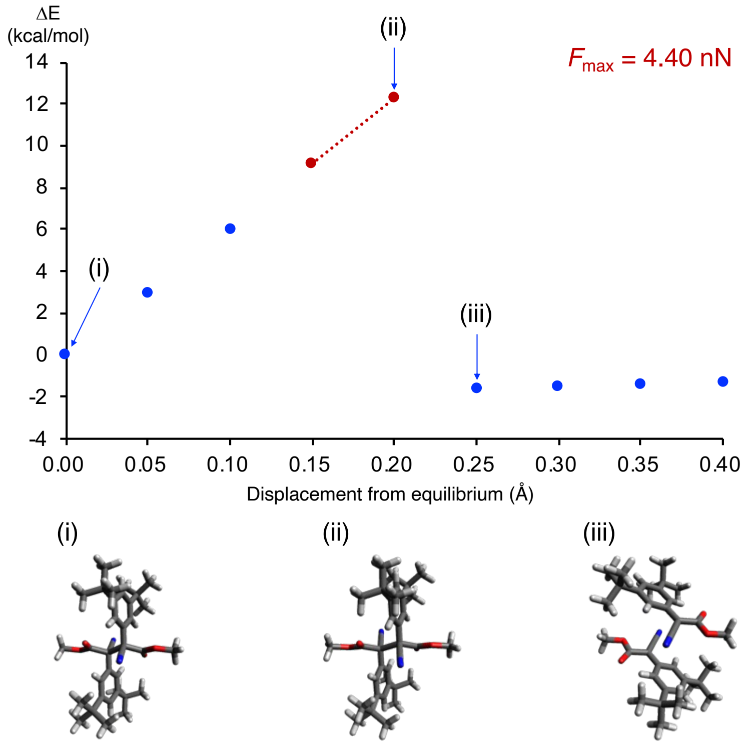

Fig. S52. Potential energy curve of BiACA-H (3,5-*t*-Bu_2) with corresponding structures at initial state, F_{max} and after central C–C bond dissociation.

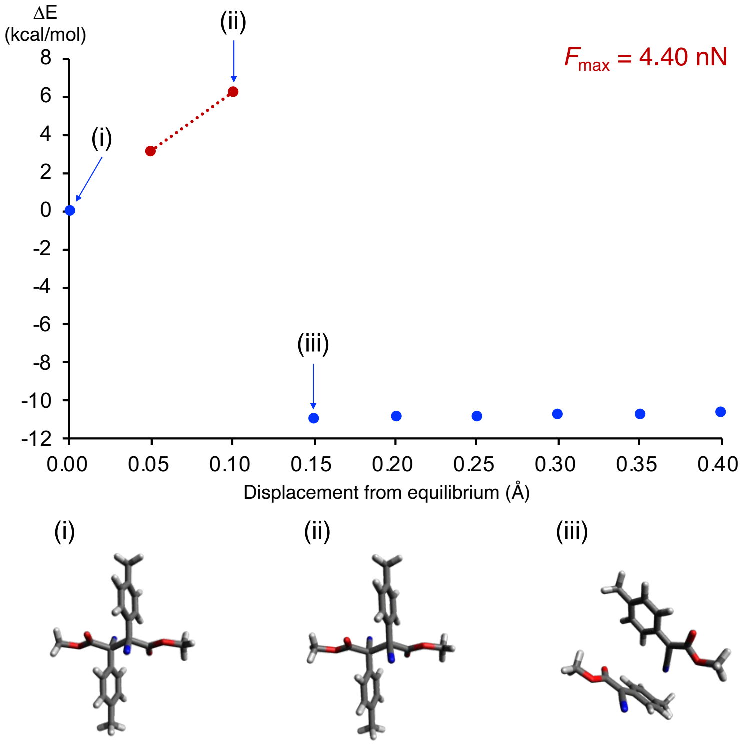

Fig. S53. Potential energy curve of BiACA-Me with corresponding structures at initial state, F_{max} and after central C–C bond dissociation.

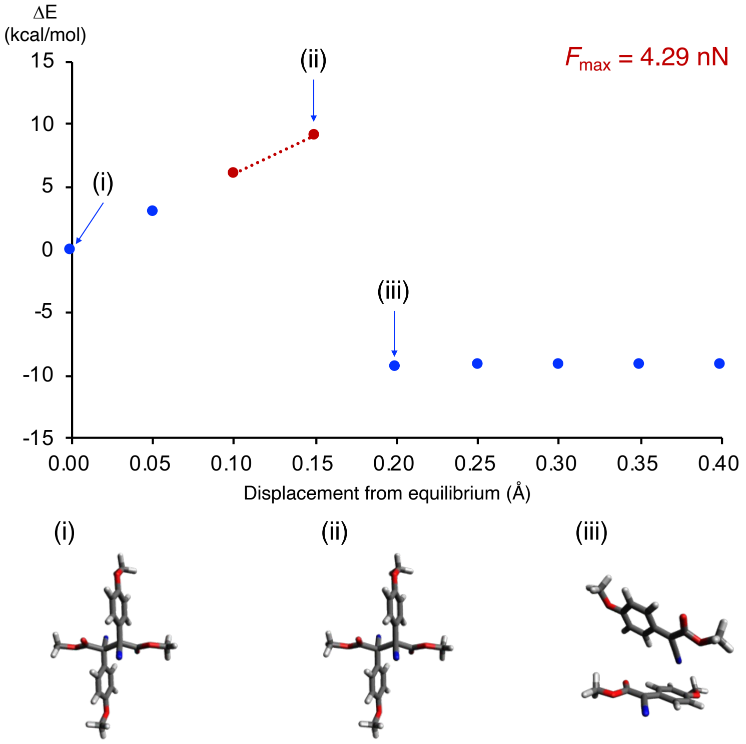

Fig. S54. Potential energy curve of BiACA-OMe with corresponding structures at initial state, F_{max} and after central C–C bond dissociation.

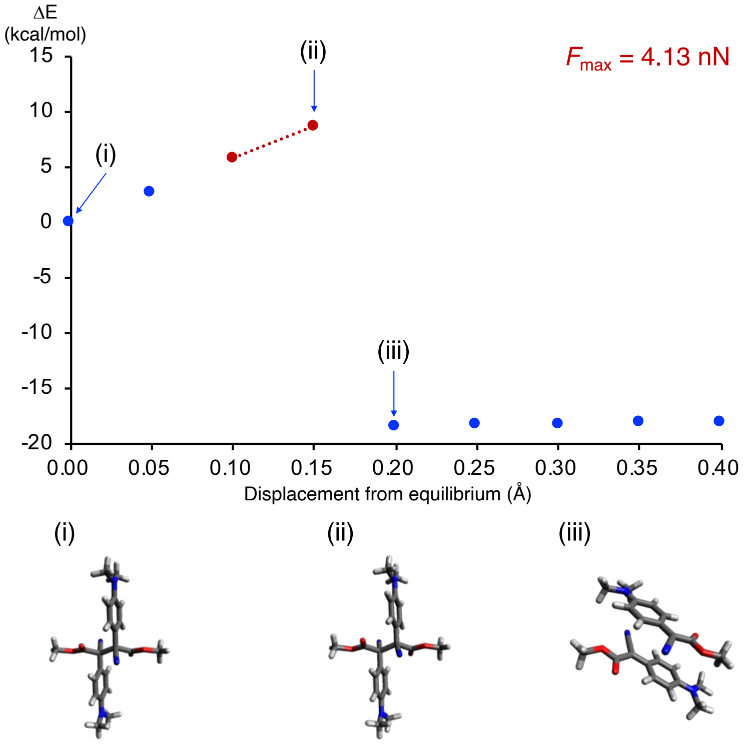

Fig. S55. Potential energy curve of BiACA-NMe_2 with corresponding structures at initial state, F_{max} and after central C–C bond dissociation.

8. Computational details

8-1. Geometry optimization

Cartesian coordinates of all optimized structures at each calculated level were attached to ZIP. All structure were checked not only imaginary vibration but also spin contamination <S^2> under 0.76 at all calculation levels. Geometry optimization were performed with the following options. Empiricaldispersion=gd3 was added to option as needed.

```
%chk=(objective structure name).chk
#p opt freq=noraman (functionals)/6-31++G(d,p) int=grid=superfine scf=(maxcyc=333,direct,tight)
```

8-2. Single point energy calculation at ROMP2/6-31++G(d,p)// UB3LYP/6-31++G(d,p)

Single point energy calculations were performed with the following options.

```
%chk=(objective structure name).chk
#p sp romp2/6-31++G(d,p) int=grid=superfine scf=(maxcyc=333,direct,tight)
```

8-3. Input files of CoGEF calculation at B3LYP/6-31G(d) level

<u>BiACA-NO_2</u>

```
%chk=BACA-NO2_b3lyp_6-31Gd_opt-freq_CoGEF_01A-45.chk
# opt=modredundant b3lyp/6-31g(d) pop=npa

Title Card Required

0 1
C        1.93753     0.20883    -0.45066
C        2.58414     1.17141     0.34146
C        3.96198     1.12140     0.53483
C        4.69034     0.10556    -0.07506
C        4.07985    -0.85547    -0.87440
C        2.70179    -0.79727    -1.06051
H        2.01990     1.96256     0.81789
H        4.47088     1.85625     1.14596
H        4.68032    -1.62553    -1.34251
H        2.22387    -1.53409    -1.69476
C        0.40033     0.26638    -0.65372
C        0.02227    -0.53065    -1.83647
```

C	0.01625	1.73263	-1.05843
O	0.00027	2.10244	-2.20511
O	-0.23300	2.50996	-0.00053
C	-0.54406	3.89195	-0.29936
H	-0.68851	4.36557	0.67062
H	-1.45203	3.94568	-0.90341
H	0.28403	4.35202	-0.84203
N	-0.25956	-1.13733	-2.78310
N	0.25815	1.13802	2.78348
C	-0.02305	0.53141	1.83662
H	-2.22439	1.53533	1.69308
O	0.00000	-2.10164	2.20538
C	-2.70214	0.79788	1.05943
C	-0.40049	-0.26580	0.65380
H	-4.68084	1.62606	1.34060
C	-0.01586	-1.73186	1.05869
C	-4.08021	0.85568	0.87321
C	-1.93770	-0.20866	0.45054
O	0.23389	-2.50917	0.00086
C	-4.69051	-0.10624	0.07480
H	1.45355	-3.94411	0.90414
C	-2.58414	-1.17212	-0.34065
C	0.54569	-3.89097	0.29988
H	-0.28227	-4.35149	0.84238
H	-2.01974	-1.96364	-0.81628
C	-3.96198	-1.12254	-0.53409
H	0.69064	-4.36458	-0.67003
H	-4.47075	-1.85806	-1.14453
N	-6.15052	-0.05084	-0.12462
N	6.15033	0.04971	0.12430
O	-6.66317	-0.92961	-0.81468
O	-6.76182	0.87155	0.41174
O	6.66318	0.92786	0.81499
O	6.76144	-0.87240	-0.41276

B 16 35 S 45 0.1

<u>BiACA-CN</u>

%chk=BACA-CN_b3lyp_6-31Gd_opt-freq_CoGEF_01A-45.chk
opt=modredundant b3lyp/6-31g(d) pop=npa

Title Card Required

0 1

C	1.94102	-0.24021	0.42174
C	2.57943	-1.15173	-0.43375
C	3.95476	-1.09161	-0.63475
C	4.72173	-0.12057	0.02564
C	4.09123	0.78536	0.89162
C	2.71548	0.72183	1.08581
H	2.00940	-1.91063	-0.95403
H	4.43676	-1.79842	-1.30172
H	4.68078	1.53138	1.41411
H	2.24634	1.41973	1.76908
C	0.40549	-0.30523	0.63341
C	0.04063	0.42161	1.86465
C	0.01821	-1.79061	0.95375
O	0.00434	-2.22768	2.07670
O	-0.23666	-2.50494	-0.14688
C	-0.55146	-3.90025	0.07304
H	-0.70290	-4.31654	-0.92190
H	-1.45636	-3.98595	0.67807
H	0.27750	-4.39434	0.58365
N	-0.22887	0.97244	2.84832
N	0.22964	-0.97245	-2.84792
C	-0.04011	-0.42160	-1.86434
H	-2.24615	-1.41875	-1.77034
O	-0.00382	2.22764	-2.07647
C	-2.71531	-0.72129	-1.08663
C	-0.40527	0.30524	-0.63319
H	-4.68066	-1.53039	-1.41583
C	-0.01823	1.79069	-0.95348
C	-4.09110	-0.78483	-0.89267
C	-1.94084	0.24014	-0.42171
O	0.23575	2.50521	0.14722
C	-4.72161	0.12045	-0.02603

H	1.45555	3.98675	-0.67663
C	-2.57926	1.15104	0.43445
C	0.55000	3.90066	-0.07263
H	-0.27866	4.39416	-0.58430
H	-2.00924	1.90950	0.95536
C	-3.95462	1.09087	0.63525
H	0.70008	4.31724	0.92241
H	-4.43663	1.79718	1.30274
C	-6.14026	0.05785	0.17595
C	6.14035	-0.05802	-0.17655
N	-7.29055	0.00525	0.33893
N	7.29062	-0.00545	-0.33971

B 16 35 S 45 0.1

<u>BiACA-COMe</u>

%chk=BACA-COMe_b3lyp_6-31Gd_opt-freq_CoGEF_01A-45.chk

opt=(modredundant, maxcycle=500) b3lyp/6-31g(d) pop=npa scf=(maxcycle=500)

Title Card Required

0 1

C	-1.94079	-0.22433	-0.43789
C	-2.57482	-1.20815	0.33961
C	-3.95091	-1.17294	0.53328
C	-4.73479	-0.16539	-0.04434
C	-4.10195	0.80988	-0.82493
C	-2.72290	0.78075	-1.02205
H	-1.99840	-2.00060	0.79990
H	-4.44361	-1.92989	1.13417
H	-4.67886	1.59968	-1.29560
H	-2.25891	1.53704	-1.64419
C	-0.40426	-0.25955	-0.65361
C	-0.04733	0.55968	-1.82834
C	-0.00547	-1.71265	-1.08303
O	0.01695	-2.06673	-2.23520
O	0.24931	-2.50721	-0.03816
C	0.56756	-3.87952	-0.36118

H	0.71627	-4.36993	0.59998
H	1.47489	-3.91857	-0.96770
H	-0.25819	-4.33616	-0.91066
N	0.21099	1.18238	-2.77134
N	-0.21054	-1.18218	2.77178
C	0.04758	-0.55953	1.82870
H	2.25890	-1.53767	1.64338
O	-0.01559	2.06707	2.23553
C	2.72290	-0.78112	1.02156
C	0.40435	0.25967	0.65390
H	4.67880	-1.60041	1.29435
C	0.00569	1.71279	1.08340
C	4.10192	-0.81031	0.82418
C	1.94086	0.22437	0.43804
O	-0.25024	2.50714	0.03865
C	4.73476	0.16530	0.04399
H	-1.47513	3.91831	0.96939
C	2.57486	1.20853	-0.33903
C	-0.56853	3.87942	0.36178
H	0.25775	4.33642	0.91017
H	1.99843	2.00129	-0.79879
C	3.95091	1.17326	-0.53295
H	-0.71854	4.36960	-0.59930
H	4.44362	1.93045	-1.13353
C	6.21824	0.18849	-0.19628
C	-6.21831	-0.18865	0.19564
O	6.71819	1.07153	-0.87435
O	-6.71831	-1.07138	0.87408
C	7.07808	-0.90181	0.41923
H	6.99501	-0.89890	1.51276
H	8.11750	-0.72872	0.13609
H	6.76735	-1.89395	0.07029
C	-7.07808	0.90134	-0.42050
H	-6.99438	0.89856	-1.51398
H	-6.76785	1.89354	-0.07126
H	-8.11765	0.72796	-0.13801

B 16 35 S 45 0.1

BiACA-COOMe

%chk=BACA-COOMe_b3lyp_6-31Gd_opt-freq_CoGEF_01A-45.chk

opt=modredundant b3lyp/6-31g(d) pop=npa

Title Card Required

0 1

C	1.92235	0.11248	-0.54552
C	2.64301	1.08370	0.16659
C	4.02528	0.98077	0.30293
C	4.71470	-0.09074	-0.27480
C	4.00088	-1.05426	-0.99534
C	2.62042	-0.95354	-1.13222
H	2.13224	1.92080	0.62501
H	4.56948	1.73533	0.85894
H	4.54403	-1.87654	-1.44897
H	2.08664	-1.70130	-1.70684
C	0.38043	0.22118	-0.68223
C	-0.08049	-0.61107	-1.81084
C	0.02679	1.67950	-1.13278
O	-0.04676	2.00206	-2.29211
O	-0.13034	2.51401	-0.09990
C	-0.41793	3.88763	-0.44542
H	-0.48924	4.41069	0.50733
H	-1.35958	3.94352	-0.99560
H	0.38794	4.29646	-1.05846
N	-0.42436	-1.24494	-2.71841
N	0.42509	1.24440	2.71824
C	0.08093	0.61063	1.81070
H	-2.08649	1.70064	1.70783
O	0.04550	-2.00247	2.29208
C	-2.62028	0.95325	1.13272
C	-0.38028	-0.22141	0.68207
H	-4.54389	1.87609	1.45000
C	-0.02718	-1.67988	1.13269
C	-4.00075	1.05405	0.99593
C	-1.92223	-0.11245	0.54545

O	0.13041	-2.51432	0.09989
C	-4.71459	0.09092	0.27488
H	1.35849	-3.94419	0.99666
C	-2.64293	-1.08335	-0.16707
C	0.41744	-3.88810	0.44550
H	-0.38917	-4.29685	1.05762
H	-2.13218	-1.92028	-0.62586
C	-4.02519	-0.98028	-0.30341
H	0.48964	-4.41098	-0.50728
H	-4.56940	-1.73458	-0.85977
C	-6.19383	0.25439	0.16143
O	-6.81785	1.18059	0.64002
O	-6.76989	-0.74723	-0.53800
C	-8.19537	-0.64929	-0.68941
H	-8.68522	-0.65457	0.28780
H	-8.48721	-1.52381	-1.27126
H	-8.46191	0.27089	-1.21579
C	6.19396	-0.25409	-0.16124
O	6.81808	-1.18038	-0.63953
O	6.76993	0.74787	0.53780
C	8.19543	0.65015	0.68924
H	8.46196	-0.26909	1.21728
H	8.48736	1.52568	1.26953
H	8.68519	0.65361	-0.28802

B 16 35 S 45 0.1

<u>BiACA-H</u>
%chk=BACA-H_b3lyp_6-31Gd_opt-freq_CoGEF_01A-45.chk
opt=modredundant b3lyp/6-31g(d) pop=npa

Title Card Required

0 1

C	1.96248	0.26208	-0.30791
C	2.54256	1.18459	0.57618
C	3.90835	1.13205	0.85464
C	4.71621	0.16787	0.25301

C	4.14772	-0.74502	-0.63493
C	2.78253	-0.69872	-0.91603
H	1.93520	1.94151	1.05607
H	4.33718	1.85281	1.54495
H	5.78017	0.13172	0.46986
H	4.76615	-1.49536	-1.11965
H	2.35847	-1.40573	-1.61969
C	0.43996	0.30948	-0.60779
C	0.15610	-0.42024	-1.85945
C	0.04835	1.78605	-0.94872
O	0.06849	2.22529	-2.07169
O	-0.25608	2.50316	0.13929
C	-0.58405	3.88868	-0.09990
H	-0.77998	4.30853	0.88603
H	-1.46727	3.95806	-0.73861
H	0.25452	4.39556	-0.58216
N	-0.04383	-0.97108	-2.85969
N	0.04461	0.97038	2.85972
C	-0.15563	0.41986	1.85937
H	-2.35794	1.40564	1.62004
O	-0.07077	-2.22585	2.07151
C	-2.78215	0.69889	0.91619
C	-0.43978	-0.30964	0.60768
H	-4.76562	1.49581	1.12009
C	-0.04894	-1.78644	0.94861
C	-4.14733	0.74550	0.63514
C	-1.96226	-0.26189	0.30782
O	0.25675	-2.50336	-0.13908
C	-4.71598	-0.16708	-0.25303
H	1.46601	-3.95895	0.74038
C	-2.54250	-1.18411	-0.57644
C	0.58389	-3.88911	0.10022
H	-0.25567	-4.39581	0.58094
H	-5.77993	-0.13074	-0.46987
H	-1.93530	-1.94105	-1.05652
C	-3.90830	-1.13125	-0.85488
H	0.78126	-4.30868	-0.88553
H	-4.33723	-1.85177	-1.54538

B 17 36 S 45 0.1

BiACA-H (3,5-*t*-Bu_2)
%chk=BACA-H-tBu2_b3lyp_6-31Gd_opt-freq_CoGEF_01A-45.chk
opt=modredundant b3lyp/6-31g(d) pop=npa

Title Card Required

0 1

C	-1.95690	0.01192	-0.44746
C	-2.56667	-1.22890	-0.20341
C	-3.93839	-1.32023	0.03954
C	-4.69457	-0.13606	0.00968
C	-4.12503	1.11214	-0.25070
C	-2.73964	1.16587	-0.47470
H	-1.96239	-2.12334	-0.19534
H	-2.26922	2.11884	-0.68396
C	-0.42287	0.08746	-0.68762
C	-0.08200	1.37160	-1.33260
C	-0.04371	-0.97506	-1.77153
O	-0.03313	-0.72969	-2.95275
O	0.20919	-2.18112	-1.24908
C	0.50061	-3.22256	-2.20467
H	0.64307	-4.12352	-1.60901
H	1.40611	-2.97522	-2.76283
H	-0.33430	-3.34007	-2.89893
N	0.16241	2.36970	-1.86951
N	-0.16325	-2.36908	1.86943
C	0.08145	-1.37106	1.33251
H	2.26893	-2.11858	0.68523
O	0.03435	0.73043	2.95269
C	2.73944	-1.16577	0.47542
C	0.42270	-0.08705	0.68748
C	0.04408	0.97561	1.77142
C	4.12486	-1.11227	0.25151
C	1.95676	-0.01179	0.44735
O	-0.20919	2.18157	1.24893

C	4.69449	0.13572	-0.00965
H	-1.40479	2.97603	2.76354
C	2.56664	1.22884	0.20261
C	-0.49977	3.22323	2.20454
H	0.33575	3.34088	2.89804
H	1.96243	2.12332	0.19395
C	3.93838	1.31992	-0.04031
H	-0.64272	4.12406	1.60880
H	5.76054	0.20023	-0.19177
H	-5.76061	-0.20075	0.19181
C	-4.95068	2.41197	-0.31165
C	-4.81824	3.03590	-1.72169
H	-3.77800	3.26971	-1.97032
H	-5.39381	3.96777	-1.78067
H	-5.19877	2.35239	-2.48910
C	-6.44624	2.17249	-0.03073
H	-6.98693	3.12397	-0.08459
H	-6.61373	1.75418	0.96846
H	-6.89702	1.49648	-0.76609
C	-4.41995	3.41267	0.74227
H	-3.37002	3.66946	0.56666
H	-4.50265	2.99807	1.75382
H	-4.99871	4.34366	0.70852
C	-4.63042	-2.66297	0.34279
C	-3.64706	-3.84895	0.31324
H	-3.19242	-3.97473	-0.67664
H	-4.18372	-4.77535	0.54695
H	-2.84316	-3.73543	1.04861
C	-5.73686	-2.92600	-0.70599
H	-6.23843	-3.87875	-0.49728
H	-5.31559	-2.97850	-1.71654
H	-6.50131	-2.14180	-0.70314
C	-5.26340	-2.59991	1.75371
H	-4.49780	-2.42681	2.51809
H	-5.76835	-3.54551	1.98575
H	-6.00549	-1.79826	1.83329
C	4.95041	-2.41212	0.31337
C	6.44601	-2.17291	0.03242

H	6.61362	-1.75519	-0.96700
H	6.98663	-3.12440	0.08687
H	6.89678	-1.49651	0.76743
C	4.81781	-3.03512	1.72380
H	5.19835	-2.35115	2.49080
H	5.39329	-3.96701	1.78342
H	3.77754	-3.26867	1.97251
C	4.41971	-3.41348	-0.73994
H	4.50252	-2.99954	-1.75175
H	3.36974	-3.67009	-0.56425
H	4.99841	-4.34448	-0.70552
C	4.63051	2.66243	-0.34436
C	5.73705	2.92598	0.70418
H	5.31586	2.97907	1.71473
H	6.23866	3.87858	0.49488
H	6.50145	2.14172	0.70171
C	3.64726	3.84852	-0.31541
H	3.19270	3.97488	0.67444
H	2.84329	3.73469	-1.05065
H	4.18399	4.77474	-0.54965
C	5.26338	2.59851	-1.75530
H	6.00540	1.79675	-1.83446
H	5.76839	3.54394	-1.98793
H	4.49771	2.42502	-2.51952

B 14 32 S 45 0.1

<u>BiACA-Me</u>
%chk=BACA-Me_b3lyp_6-31Gd_opt-freq_CoGEF_01A-45.chk
opt=modredundant b3lyp/6-31g(d) pop=npa

Title Card Required

0 1

C	1.94700	0.24560	-0.39756
C	2.58069	1.16270	0.45434
C	3.95597	1.09722	0.66687
C	4.74772	0.12681	0.04163

C	4.10991	-0.77492	-0.81760
C	2.73490	-0.72009	-1.03746
H	2.00794	1.93010	0.95976
H	4.41973	1.82125	1.33250
H	4.69680	-1.53130	-1.33347
H	2.27903	-1.42528	-1.72308
C	0.41446	0.30501	-0.62775
C	0.06808	-0.42519	-1.86347
C	0.01651	1.78294	-0.95283
O	-0.02273	2.21917	-2.07670
O	-0.22115	2.50670	0.14762
C	-0.55115	3.89346	-0.07854
H	-0.68971	4.31827	0.91502
H	-1.46751	3.96775	-0.66819
H	0.26342	4.39246	-0.60795
N	-0.18100	-0.97667	-2.85228
N	0.18069	0.97688	2.85232
C	-0.06828	0.42533	1.86352
H	-2.27918	1.42556	1.72266
O	0.02313	-2.21898	2.07675
C	-2.73503	0.72019	1.03720
C	-0.41453	-0.30493	0.62779
H	-4.69693	1.53137	1.33305
C	-0.01637	-1.78281	0.95286
C	-4.11001	0.77491	0.81734
C	-1.94706	-0.24564	0.39753
O	0.22119	-2.50657	-0.14758
C	-4.74779	-0.12706	-0.04172
H	1.46791	-3.96739	0.66808
C	-2.58072	-1.16299	-0.45409
C	0.55146	-3.89328	0.07856
H	-0.26296	-4.39239	0.60810
H	-2.00794	-1.93052	-0.95928
C	-3.95602	-1.09762	-0.66665
H	0.68994	-4.31808	-0.91501
H	-4.41974	-1.82185	-1.33206
C	-6.23319	-0.04672	-0.30009
H	-6.76088	0.42385	0.53584

H	-6.66651	-1.03967	-0.46051
H	-6.44553	0.54848	-1.19816
C	6.23312	0.04650	0.29999
H	6.66709	1.03978	0.45660
H	6.76033	-0.42748	-0.53431
H	6.44529	-0.54542	1.20026

B 16 35 S 45 0.1

BiACA-OMe

%chk=BACA-OMe_b3lyp_6-31Gd_opt-freq_CoGEF_01A-45.chk

opt=(modredundant, maxcycle=500) b3lyp/6-31g(d) pop=npa scf=(maxcycle=500)

Title Card Required

0 1

C	1.93102	0.11318	-0.51757
C	2.66756	1.01730	0.25721
C	4.04857	0.88536	0.41001
C	4.72425	-0.16323	-0.22323
C	3.99853	-1.06693	-1.01273
C	2.62605	-0.92779	-1.15732
H	2.17203	1.83693	0.76229
H	4.57818	1.60485	1.02323
H	4.53346	-1.86946	-1.51046
H	2.09049	-1.63170	-1.78408
C	0.39572	0.25527	-0.66216
C	-0.06465	-0.50057	-1.84428
C	0.05969	1.73912	-1.02573
O	-0.03541	2.13229	-2.16222
O	-0.05821	2.52029	0.05538
C	-0.33001	3.91262	-0.20968
H	-0.37473	4.38519	0.77093
H	-1.28112	4.01358	-0.73703
H	0.46952	4.34346	-0.81633
N	-0.40452	-1.07586	-2.79167
N	0.40519	1.07522	2.79155
C	0.06512	0.50006	1.84415

H	-2.09004	1.63111	1.78485
O	0.03351	-2.13292	2.16226
C	-2.62572	0.92756	1.15777
C	-0.39554	-0.25561	0.66206
H	-4.53301	1.86931	1.51137
C	-0.06025	-1.73961	1.02570
C	-3.99818	1.06695	1.01326
C	-1.93083	-0.11320	0.51753
O	0.05859	-2.52064	-0.05536
C	-4.72401	0.16369	0.22335
H	1.27981	-4.01457	0.73848
C	-2.66748	-1.01687	-0.25765
C	0.32951	-3.91314	0.20976
H	-0.47107	-4.34381	0.81515
H	-2.17207	-1.83636	-0.76310
C	-4.04847	-0.88469	-0.41039
H	0.37546	-4.38549	-0.77089
H	-4.57817	-1.60382	-1.02395
O	-6.06342	0.38737	0.14328
O	6.06369	-0.38671	-0.14305
C	-6.84882	-0.49425	-0.64722
H	-7.87372	-0.12693	-0.57359
H	-6.80373	-1.52306	-0.26723
H	-6.53238	-0.48162	-1.69806
C	6.84897	0.49541	0.64701
H	7.87391	0.12816	0.57361
H	6.53249	0.48332	1.69785
H	6.80379	1.52402	0.26647

B 16 35 S 45 0.1

<u>BiACA-NMe$_2$</u>

%chk=BACA-NMe2_b3lyp_6-31Gd_opt-freq_CoGEF_01A-45.chk

opt=(modredundant, maxcycle=500) b3lyp/6-31g(d) pop=npa scf=(maxcycle=500)

Title Card Required

0 1

C	2.01471	-0.14061	0.48644
C	2.54899	-1.30151	-0.09272
C	3.91234	-1.44195	-0.31557
C	4.82497	-0.42245	0.03687
C	4.28109	0.74916	0.60987
C	2.91672	0.87704	0.82831
H	1.89725	-2.11065	-0.39598
H	4.26174	-2.35732	-0.77716
H	4.92299	1.57621	0.88710
H	2.55660	1.79723	1.27445
C	0.49834	0.02185	0.73585
C	0.31382	1.01439	1.81213
C	-0.10483	-1.30571	1.27098
O	-0.33906	-1.51506	2.43675
O	-0.30434	-2.19054	0.28419
C	-0.88936	-3.44510	0.68546
H	-0.97965	-4.02511	-0.23237
H	-1.87007	-3.27144	1.13387
H	-0.24344	-3.95153	1.40646
N	0.20658	1.84124	2.61888
N	0.56169	-0.70319	-2.72114
C	0.18622	-0.16603	-1.76431
H	-2.05797	-0.71395	-2.37314
O	1.05363	2.40122	-1.27946
C	-2.57587	-0.37742	-1.48134
C	-0.35137	0.52845	-0.58232
H	-4.42937	-1.12225	-2.21575
C	0.01060	2.03417	-0.79034
C	-3.94299	-0.61511	-1.39119
C	-1.86565	0.26598	-0.45932
O	-0.94408	2.84985	-0.33337
C	-4.68310	-0.21317	-0.25771
H	0.22634	4.47006	0.25803
C	-2.59737	0.66102	0.67139
C	-0.63062	4.25505	-0.38482
H	-0.40320	4.55559	-1.41006
H	-2.11271	1.17393	1.49251
C	-3.96182	0.42225	0.77833

H	-1.52318	4.76106	-0.01822
H	-4.46506	0.74343	1.68219
N	-6.04998	-0.42414	-0.16744
N	6.18735	-0.56804	-0.16137
C	-6.73106	-0.17608	1.09220
H	-7.79650	-0.37879	0.96786
H	-6.62762	0.87243	1.39659
H	-6.35367	-0.80664	1.91278
C	6.68193	-1.69991	-0.92754
H	7.77207	-1.65657	-0.96477
H	6.30134	-1.71306	-1.96084
H	6.40507	-2.64852	-0.45176
C	7.06537	0.57310	0.03770
H	8.09707	0.26522	-0.14258
H	7.00552	0.94019	1.06934
H	6.83256	1.41202	-0.63676
C	-6.71543	-1.23835	-1.16965
H	-6.34109	-2.27437	-1.19649
H	-6.59220	-0.80861	-2.17110
H	-7.78513	-1.26764	-0.95368

B 16 35 S 45 0.1

7-4. Input files of CoGEF calculation at UB3LYP/6-31++G(d,p) level

BiACA-NO_2

%chk=BACA-NO2_b3lyp_6-31Gd_opt-freq_CoGEF_01A-45_str-19_ub3lyp_6-31++Gdp_opt_BF_CoGEF_005-8_ub3lyp_6-31++Gdp.chk

opt=modredundant ub3lyp/6-31++g(d,p) pop=npa

Title Card Required

0 1

C	1.95300	0.40771	-0.28132
C	2.63517	0.77119	0.89184
C	4.00006	0.52928	1.02383
C	4.67761	-0.07716	-0.03172
C	4.02820	-0.44765	-1.20746

C	2.66253	-0.20238	-1.32665
H	2.10623	1.23958	1.71432
H	4.53525	0.80347	1.92422
H	4.58638	-0.91621	-2.00829
H	2.14928	-0.49378	-2.23509
C	0.44910	0.67242	-0.39390
C	0.00199	0.69812	-1.78396
C	0.17916	2.23738	0.21055
O	-0.40089	2.40378	1.25381
O	0.72175	3.20684	-0.57042
C	0.74013	4.77512	-0.28993
H	0.64898	5.17733	-1.29396
H	1.70618	4.96833	0.16997
H	-0.09953	5.01468	0.35935
N	-0.38110	0.74471	-2.87929
N	0.38108	-0.74473	2.87929
C	-0.00199	-0.69813	1.78395
H	-2.14928	0.49374	2.23510
O	0.40088	-2.40378	-1.25382
C	-2.66254	0.20236	1.32666
C	-0.44911	-0.67243	0.39390
H	-4.58638	0.91619	2.00831
C	-0.17916	-2.23739	-0.21056
C	-4.02820	0.44764	1.20748
C	-1.95301	-0.40772	0.28132
O	-0.72174	-3.20686	0.57040
C	-4.67762	0.07716	0.03173
H	-1.70608	-4.96834	-0.17016
C	-2.63518	-0.77117	-0.89185
C	-0.74009	-4.77513	0.28990
H	0.09966	-5.01469	-0.35925
H	-2.10624	-1.23955	-1.71433
C	-4.00006	-0.52926	-1.02383
H	-0.64910	-5.17735	1.29394
H	-4.53525	-0.80342	-1.92423
N	-6.12517	0.33023	-0.09808
N	6.12517	-0.33021	0.09809
O	-6.67704	-0.02537	-1.14018

O	-6.69637	0.88168	0.84337
O	6.67703	0.02540	1.14019
O	6.69637	-0.88168	-0.84334

B 16 35 S 8 0.05

BiACA-CN

%chk=BACA-CN_b3lyp_6-31Gd_opt-freq_CoGEF_01A-45_str-19_ub3lyp_6-31++Gdp_opt_BF_CoGEF_005-8_ub3lyp_6-31++Gdp.chk

opt=modredundant ub3lyp/6-31++g(d,p) pop=npa

Title Card Required

0 1

C	1.96680	-0.35899	0.25884
C	2.65287	-0.66751	-0.92695
C	4.00790	-0.37893	-1.05863
C	4.70279	0.22173	0.00366
C	4.02247	0.53046	1.19304
C	2.66639	0.24231	1.31516
H	2.13312	-1.12841	-1.75966
H	4.52846	-0.61761	-1.97961
H	4.55635	0.99318	2.01595
H	2.15136	0.49278	2.23500
C	0.47238	-0.67041	0.37279
C	0.03514	-0.74608	1.76416
C	0.24440	-2.22401	-0.27307
O	-0.32721	-2.37891	-1.32290
O	0.81082	-3.20109	0.48290
C	0.87359	-4.75772	0.15668
H	0.86726	-5.18663	1.15367
H	1.81159	-4.90417	-0.37362
H	0.00102	-5.01604	-0.43989
N	-0.33828	-0.83158	2.86052
N	0.33830	0.83169	-2.86050
C	-0.03513	0.74616	-1.76415
H	-2.15134	-0.49267	-2.23506
O	0.32715	2.37890	1.32297

C	-2.66637	-0.24226	-1.31521
C	-0.47239	0.67045	-0.37278
H	-4.55633	-0.99313	-2.01604
C	-0.24442	2.22403	0.27312
C	-4.02246	-0.53044	-1.19310
C	-1.96680	0.35900	-0.25885
O	-0.81082	3.20113	-0.48285
C	-4.70278	-0.22178	-0.00370
H	-1.81218	4.90432	0.37262
C	-2.65288	0.66744	0.92695
C	-0.87359	4.75776	-0.15661
H	-0.00164	5.01585	0.44097
H	-2.13313	1.12831	1.75969
C	-4.00790	0.37883	1.05861
H	-0.86605	5.18678	-1.15355
H	-4.52846	0.61746	1.97961
C	-6.10213	-0.51457	0.12414
C	6.10214	0.51448	-0.12420
N	-7.23701	-0.75074	0.22707
N	7.23703	0.75063	-0.22715

B 16 35 S 8 0.05

<u>BiACA-COMe</u>

%chk=BACA-COMe_b3lyp_6-31Gd_opt-freq_CoGEF_01A-45_str-19_ub3lyp_6-31++Gdp_opt_BF_CoGEF_005-8_ub3lyp_6-31++Gdp.chk

opt=modredundant ub3lyp/6-31++g(d,p) pop=npa

Title Card Required

0 1

C	1.95583	-0.42560	0.26091
C	2.62483	-0.81988	-0.91099
C	3.99003	-0.59624	-1.05318
C	4.72523	0.02408	-0.03126
C	4.05417	0.41852	1.13565
C	2.68562	0.19656	1.28223
H	2.08098	-1.29743	-1.71906

H	4.50852	-0.89959	-1.95625
H	4.59114	0.90045	1.94561
H	2.18665	0.51410	2.19025
C	0.45111	-0.67221	0.39788
C	0.02368	-0.67256	1.79472
C	0.16079	-2.24199	-0.17775
O	-0.42433	-2.42636	-1.21519
O	0.69774	-3.20781	0.61775
C	0.70446	-4.77296	0.35235
H	0.79192	-5.15923	1.36284
H	1.57950	-4.96730	-0.26390
H	-0.22842	-5.03941	-0.14057
N	-0.33771	-0.69984	2.89811
N	0.33776	0.69973	-2.89809
C	-0.02366	0.67244	-1.79470
H	-2.18665	-0.51430	-2.19016
O	0.42431	2.42627	1.21521
C	-2.68563	-0.19667	-1.28218
C	-0.45111	0.67212	-0.39787
H	-4.59116	-0.90055	-1.94553
C	-0.16075	2.24190	0.17773
C	-4.05420	-0.41856	-1.13560
C	-1.95583	0.42555	-0.26090
O	-0.69768	3.20772	-0.61778
C	-4.72526	-0.02400	0.03126
H	-1.57921	4.96719	0.26419
C	-2.62485	0.81997	0.91095
C	-0.70439	4.77287	-0.35237
H	0.22866	5.03937	0.14022
H	-2.08100	1.29760	1.71898
C	-3.99006	0.59640	1.05313
H	-0.79221	5.15912	-1.36284
H	-4.50856	0.89986	1.95616
C	-6.19961	-0.23283	0.22895
C	6.19957	0.23298	-0.22896
O	-6.74766	0.15183	1.25326
O	6.74762	-0.15156	-1.25331
C	-6.99895	-0.92613	-0.85919

H	-6.94916	-0.36581	-1.79976
H	-8.03822	-0.99845	-0.53695
H	-6.60770	-1.93074	-1.05541
C	6.99890	0.92622	0.85923
H	6.94916	0.36580	1.79974
H	6.60762	1.93079	1.05556
H	8.03816	0.99860	0.53697

B 16 35 S 8 0.05

<u>BiACA-COOMe</u>

%chk=BACA-COOMe_b3lyp_6-31Gd_opt-freq_CoGEF_01A-45_str-19_ub3lyp_6-31++Gdp_opt_BF_CoGEF_005-8_ub3lyp_6-31++Gdp.chk

opt=modredundant ub3lyp/6-31++g(d,p) pop=npa

Title Card Required

0 1

C	1.93155	0.43527	-0.38794
C	2.67714	0.80231	0.74389
C	4.04986	0.57025	0.79384
C	4.70253	-0.03053	-0.29095
C	3.96105	-0.39653	-1.42196
C	2.58895	-0.16684	-1.47116
H	2.19053	1.26328	1.59649
H	4.61432	0.85488	1.67382
H	4.47270	-0.86027	-2.25828
H	2.02838	-0.46223	-2.35031
C	0.42096	0.68334	-0.41424
C	-0.10523	0.71374	-1.77652
C	0.17203	2.23740	0.21779
O	-0.31485	2.39453	1.30894
O	0.62888	3.22568	-0.60078
C	0.64938	4.78249	-0.29759
H	0.77951	5.18699	-1.29615
H	1.50082	4.95803	0.35655
H	-0.29900	5.05045	0.16396
N	-0.54823	0.76602	-2.84883

N	0.54824	-0.76591	2.84880
C	0.10523	-0.71366	1.77650
H	-2.02835	0.46239	2.35021
O	0.31496	-2.39458	-1.30886
C	-2.58894	0.16694	1.47109
C	-0.42095	-0.68330	0.41422
H	-4.47268	0.86040	2.25818
C	-0.17201	-2.23739	-0.21776
C	-3.96103	0.39661	1.42189
C	-1.93154	-0.43524	0.38790
O	-0.62894	-3.22563	0.60083
C	-4.70253	0.03052	0.29092
H	-1.50051	-4.95794	-0.35696
C	-2.67714	-0.80239	-0.74389
C	-0.64946	-4.78244	0.29771
H	0.29918	-5.05054	-0.16323
H	-2.19054	-1.26342	-1.59646
C	-4.04986	-0.57034	-0.79384
H	-0.78026	-5.18686	1.29622
H	-4.61433	-0.85504	-1.67379
C	-6.17023	0.29842	0.29372
O	-6.77128	0.82316	1.21320
O	-6.77465	-0.10658	-0.84598
C	-8.19410	0.12469	-0.91849
H	-8.70783	-0.40708	-0.11410
H	-8.49950	-0.25835	-1.89175
H	-8.40999	1.19264	-0.83774
C	6.17023	-0.29845	-0.29375
O	6.77129	-0.82312	-1.21327
O	6.77464	0.10646	0.84599
C	8.19408	-0.12483	0.91850
H	8.40996	-1.19278	0.83768
H	8.49948	0.25813	1.89179
H	8.70783	0.40698	0.11416

B 16 35 S 8 0.05

<u>BiACA-H</u>

%chk=BACA-H_b3lyp_6-31Gd_opt-freq_CoGEF_01A-45_str-19_ub3lyp_6-31++Gdp_opt_BF_CoGEF_005-8_ub3lyp_6-31++Gdp.chk
opt=modredundant ub3lyp/6-31++g(d,p) pop=npa

Title Card Required

0 1

C	1.99333	-0.27899	-0.18395
C	2.70630	-0.16361	1.02005
C	3.90005	-0.86298	1.20412
C	4.39958	-1.68227	0.18938
C	3.69503	-1.79927	-1.01110
C	2.49939	-1.10405	-1.19855
H	2.33087	0.46302	1.82201
H	4.43599	-0.76490	2.14338
H	5.33011	-2.22377	0.33252
H	4.07350	-2.43241	-1.80807
H	1.95778	-1.20901	-2.13162
C	0.67495	0.47987	-0.36221
C	0.33059	0.65466	-1.77123
C	0.92642	2.03842	0.25250
O	0.43207	2.38634	1.29539
O	1.77146	2.78759	-0.51347
C	2.31035	4.24151	-0.19313
H	2.61358	4.57652	-1.17995
H	3.14296	4.11497	0.49577
H	1.49496	4.82182	0.23461
N	0.04507	0.82625	-2.88389
N	-0.04508	-0.82625	2.88389
C	-0.33059	-0.65466	1.77123
H	-1.95778	1.20902	2.13162
O	-0.43210	-2.38630	-1.29543
C	-2.49940	1.10406	1.19856
C	-0.67495	-0.47986	0.36222
H	-4.07351	2.43242	1.80808
C	-0.92641	-2.03841	-0.25251
C	-3.69504	1.79927	1.01111
C	-1.99334	0.27899	0.18395

O	-1.77142	-2.78761	0.51347
C	-4.39960	1.68226	-0.18937
H	-3.14295	-4.11498	-0.49573
C	-2.70633	0.16360	-1.02003
C	-2.31028	-4.24153	0.19311
H	-1.49491	-4.82180	-0.23471
H	-5.33013	2.22376	-0.33250
H	-2.33090	-0.46303	-1.82200
C	-3.90007	0.86297	-1.20410
H	-2.61343	-4.57659	1.17994
H	-4.43602	0.76488	-2.14335

B 17 36 S 8 0.05

<u>BiACA-H (3,5-*t*-Bu$_2$)</u>
%chk=BACA-H-tBu2_b3lyp_6-31Gd_opt-freq_CoGEF_01A-45_str-18_ub3lyp_6-31++Gdp_opt_BF_CoGEF_005-8_ub3lyp_6-31++Gdp.chk
opt=modredundant ub3lyp/6-31++g(d,p) pop=npa

Title Card Required

0 1

C	-1.96374	0.03708	0.47409
C	-2.62148	1.21159	0.07173
C	-3.98637	1.20372	-0.22378
C	-4.68068	-0.01406	-0.09630
C	-4.05755	-1.19910	0.30670
C	-2.68203	-1.15223	0.58896
H	-2.05559	2.12898	-0.02005
H	-2.16292	-2.05268	0.89359
C	-0.45564	0.08001	0.76150
C	-0.03447	-1.03243	1.61337
C	-0.17996	1.47656	1.65836
O	0.34498	2.44742	1.17219
O	-0.66938	1.40062	2.92964
C	-0.68140	2.55683	3.98879
H	-0.97024	2.02117	4.88771
H	-1.42375	3.28461	3.66564

H	0.31905	2.98287	4.04270
N	0.31764	-1.89244	2.30989
N	-0.31764	1.89242	-2.30989
C	0.03447	1.03239	-1.61339
H	2.16292	2.05264	-0.89371
O	-0.34491	-2.44748	-1.17220
C	2.68202	1.15220	-0.58903
C	0.45564	-0.08005	-0.76153
C	0.17999	-1.47660	-1.65838
C	4.05754	1.19909	-0.30676
C	1.96373	-0.03711	-0.47411
O	0.66935	-1.40062	-2.92968
C	4.68067	0.01408	0.09631
H	1.42562	-3.28326	-3.66689
C	2.62148	-1.21159	-0.07168
C	0.68144	-2.55682	-3.98885
H	-0.31835	-2.98461	-4.04103
H	2.05559	-2.12898	0.02016
C	3.98636	-1.20370	0.22385
H	0.96785	-2.02074	-4.88829
H	5.74025	0.02793	0.32014
H	-5.74026	-0.02790	-0.32012
C	-4.80967	-2.53773	0.44413
C	-4.66544	-3.06549	1.89276
H	-3.61969	-3.23508	2.16485
H	-5.19686	-4.01800	2.00221
H	-5.08833	-2.35532	2.61183
C	-6.31302	-2.40428	0.13213
H	-6.79764	-3.37943	0.24889
H	-6.49067	-2.07109	-0.89619
H	-6.81207	-1.70521	0.81224
C	-4.20172	-3.56798	-0.53936
H	-3.13612	-3.73043	-0.35450
H	-4.31212	-3.22892	-1.57538
H	-4.71342	-4.53257	-0.44039
C	-4.73753	2.46785	-0.68723
C	-3.81982	3.70461	-0.75485
H	-3.39968	3.95538	0.22550

H	-4.39930	4.56996	-1.09397
H	-2.99210	3.56387	-1.45707
C	-5.89277	2.77630	0.29611
H	-6.43380	3.67389	-0.02524
H	-5.50939	2.95417	1.30693
H	-6.61443	1.95546	0.35181
C	-5.31994	2.22614	-2.10184
H	-4.52154	2.01394	-2.82057
H	-5.86045	3.11574	-2.44564
H	-6.01957	1.38457	-2.11650
C	4.80966	2.53772	-0.44426
C	6.31300	2.40428	-0.13222
H	6.49064	2.07117	0.89612
H	6.79763	3.37943	-0.24904
H	6.81206	1.70518	-0.81228
C	4.66546	3.06539	-1.89292
H	5.08835	2.35519	-2.61194
H	5.19687	4.01790	-2.00240
H	3.61971	3.23497	-2.16503
C	4.20169	3.56801	0.53917
H	4.31208	3.22901	1.57520
H	3.13609	3.73045	0.35428
H	4.71338	4.53261	0.44015
C	4.73752	-2.46780	0.68739
C	5.89276	-2.77632	-0.29593
H	5.50939	-2.95426	-1.30673
H	6.43379	-3.67388	0.02548
H	6.61443	-1.95548	-0.35168
C	3.81981	-3.70456	0.75509
H	3.39966	-3.95538	-0.22523
H	2.99210	-3.56378	1.45731
H	4.39929	-4.56988	1.09426
C	5.31993	-2.22599	2.10199
H	6.01956	-1.38442	2.11659
H	5.86043	-3.11557	2.44584
H	4.52152	-2.01375	2.82070

B 14 32 S 8 0.05

BiACA-Me

%chk=BACA-Me_b3lyp_6-31Gd_opt-freq_CoGEF_01A-45_str-19_ub3lyp_6-31++Gdp_opt_BF_CoGEF_005-8_ub3lyp_6-31++Gdp.chk

opt=modredundant ub3lyp/6-31++g(d,p) pop=npa

Title Card Required

0 1

C	-1.99166	-0.25710	-0.23917
C	-2.69023	-0.54026	0.94439
C	-4.02784	-0.17113	1.08380
C	-4.71370	0.48422	0.05229
C	-4.00699	0.76526	-1.12527
C	-2.66882	0.40249	-1.27405
H	-2.19295	-1.03818	1.77015
H	-4.54243	-0.39583	2.01452
H	-4.50767	1.27808	-1.94266
H	-2.14946	0.64338	-2.19468
C	-0.51818	-0.64584	-0.37045
C	-0.09830	-0.73816	-1.76660
C	-0.37044	-2.20660	0.26673
O	0.16082	-2.40131	1.33121
O	-0.96213	-3.16252	-0.50873
C	-1.11027	-4.70055	-0.17198
H	-1.33379	-5.10993	-1.15201
H	-1.93600	-4.78701	0.53148
H	-0.16748	-5.05183	0.24330
N	0.25574	-0.83872	-2.86827
N	-0.25587	0.83880	2.86821
C	0.09828	0.73825	1.76657
H	2.14940	-0.64324	2.19475
O	-0.16094	2.40141	-1.33115
C	2.66878	-0.40242	1.27410
C	0.51817	0.64590	0.37043
H	4.50760	-1.27803	1.94276
C	0.37052	2.20666	-0.26677
C	4.00694	-0.76524	1.12534

C	1.99164	0.25712	0.23918
O	0.96232	3.16254	0.50865
C	4.71365	-0.48431	-0.05225
H	1.93631	4.78701	-0.53146
C	2.69021	0.54016	-0.94441
C	1.11051	4.70058	0.17192
H	0.16777	5.05188	-0.24344
H	2.19294	1.03805	-1.77021
C	4.02781	0.17098	-1.08380
H	1.33396	5.10995	1.15197
H	4.54240	0.39559	-2.01454
C	6.17215	-0.85077	-0.19405
H	6.41783	-1.74426	0.38811
H	6.81802	-0.03921	0.16471
H	6.43646	-1.04134	-1.23870
C	-6.17221	0.85061	0.19412
H	-6.81805	0.03904	-0.16466
H	-6.41793	1.74412	-0.38801
H	-6.43652	1.04115	1.23878

B 16 35 S 8 0.05

<u>BiACA-OMe</u>

%chk=BACA-OMe_b3lyp_6-31Gd_opt-freq_CoGEF_01A-45_str-18_ub3lyp_6-31++Gdp_opt_BF_CoGEF_005-8_ub3lyp_6-31++Gdp.chk

opt=modredundant ub3lyp/6-31++g(d,p) pop=npa

Title Card Required

0 1

C	-1.95578	0.33459	0.37545
C	-2.72578	0.62853	-0.75618
C	-4.08839	0.32322	-0.80772
C	-4.70410	-0.28571	0.29225
C	-3.94315	-0.58137	1.43356
C	-2.58942	-0.27573	1.47210
H	-2.27034	1.09293	-1.62439
H	-4.64565	0.56264	-1.70507

H	-4.43152	-1.05167	2.28055
H	-2.01803	-0.52052	2.36018
C	-0.45997	0.65746	0.39335
C	0.05815	0.75435	1.75783
C	-0.29017	2.18750	-0.27770
O	0.14998	2.34410	-1.38970
O	-0.76199	3.18131	0.52926
C	-0.85860	4.70174	0.16484
H	-1.07657	5.14196	1.13291
H	-1.67292	4.81208	-0.54932
H	0.09811	5.01789	-0.24766
N	0.49027	0.85727	2.83088
N	-0.49030	-0.85728	-2.83086
C	-0.05815	-0.75438	-1.75781
H	2.01802	0.52047	-2.36020
O	-0.14994	-2.34412	1.38973
C	2.58941	0.27570	-1.47212
C	0.45998	-0.65748	-0.39334
H	4.43150	1.05164	-2.28059
C	0.29019	-2.18752	0.27771
C	3.94314	0.58136	-1.43359
C	1.95578	-0.33460	-0.37545
O	0.76199	-3.18133	-0.52925
C	4.70409	0.28573	-0.29227
H	1.67285	-4.81208	0.54943
C	2.72579	-0.62851	0.75618
C	0.85861	-4.70176	-0.16482
H	-0.09814	-5.01794	0.24757
H	2.27034	-1.09288	1.62441
C	4.08839	-0.32318	0.80772
H	1.07670	-5.14197	-1.13287
H	4.64566	-0.56257	1.70507
O	6.02416	0.62530	-0.35140
O	-6.02417	-0.62526	0.35137
C	6.84675	0.36407	0.78050
H	7.84186	0.72050	0.51215
H	6.89211	-0.70950	1.00188
H	6.49151	0.90595	1.66559

C	-6.84676	-0.36401	-0.78053
H	-7.84187	-0.72043	-0.51219
H	-6.49152	-0.90587	-1.66562
H	-6.89210	0.70957	-1.00188

B 16 35 S 8 0.05

BiACA-NMe$_2$
%chk=BACA-NMe2_b3lyp_6-31Gd_opt-freq_CoGEF_01A-45_str-18_ub3lyp_6-31++Gdp_opt_BF_CoGEF_005-8_ub3lyp_6-31++Gdp.chk
opt=modredundant ub3lyp/6-31++g(d,p) pop=npa

Title Card Required

0 1

C	-1.94397	0.45400	0.30537
C	-2.64449	0.85718	-0.84154
C	-4.01649	0.66341	-0.96198
C	-4.76521	0.05947	0.07560
C	-4.05007	-0.35506	1.22428
C	-2.67788	-0.15601	1.33163
H	-2.11900	1.32011	-1.67037
H	-4.49861	0.98557	-1.87607
H	-4.56072	-0.83816	2.04761
H	-2.17267	-0.49521	2.22911
C	-0.43093	0.65825	0.40553
C	0.02065	0.65068	1.79862
C	-0.12016	2.19044	-0.17382
O	0.36760	2.37577	-1.26185
O	-0.54363	3.17544	0.67052
C	-0.51226	4.69709	0.36825
H	-0.76230	5.12167	1.33593
H	-1.26634	4.89954	-0.39126
H	0.48811	4.96444	0.03037
N	0.39590	0.67104	2.89742
N	-0.39603	-0.67093	-2.89741
C	-0.02062	-0.65062	-1.79866
H	2.17276	0.49516	-2.22916

O	-0.36747	-2.37567	1.26191
C	2.67794	0.15597	-1.33167
C	0.43095	-0.65820	-0.40556
H	4.56081	0.83804	-2.04764
C	0.12012	-2.19038	0.17380
C	4.05013	0.35499	-1.22430
C	1.94400	-0.45399	-0.30539
O	0.54349	-3.17541	-0.67055
C	4.76525	-0.05952	-0.07560
H	1.26615	-4.89951	0.39128
C	2.64450	-0.85714	0.84154
C	0.51208	-4.69705	-0.36823
H	-0.48831	-4.96435	-0.03033
H	2.11898	-1.32003	1.67038
C	4.01649	-0.66340	0.96199
H	0.76209	-5.12167	-1.33589
H	4.49859	-0.98554	1.87611
N	6.14002	0.10566	0.02340
N	-6.13997	-0.10575	-0.02338
C	6.79291	-0.11086	1.30604
H	7.86745	0.03649	1.18726
H	6.63912	-1.13720	1.65627
H	6.43292	0.57607	2.08842
C	-6.79289	0.11080	-1.30601
H	-7.86743	-0.03658	-1.18722
H	-6.43289	-0.57609	-2.08842
H	-6.63913	1.13716	-1.65620
C	-6.83153	-0.92754	0.95844
H	-7.90147	-0.91137	0.74517
H	-6.69214	-0.53057	1.96956
H	-6.49248	-1.97591	0.95164
C	6.83160	0.92740	-0.95845
H	6.49258	1.97578	-0.95168
H	6.69222	0.53040	-1.96955
H	7.90155	0.91121	-0.74516

B 16 35 S 8 0.05

9. References

1 G. E. S. M. J. Frisch, G. W. Trucks, H. B. Schlegel, V. B. M. A. Robb, J. R. Cheeseman, G. Scalmani, A. V. M. G. A. Petersson, H. Nakatsuji, X. Li, M. Caricato, H. P. H. J. Bloino, B. G. Janesko, R. Gomperts, B. Mennucci, D. W.-Y. J. V. Ortiz, A. F. Izmaylov, J. L. Sonnenberg, A. P. F. Ding, F. Lipparini, F. Egidi, J. Goings, B. Peng, N. R. T. Henderson, D. Ranasinghe, V. G. Zakrzewski, J. Gao, R. F. G. Zheng, W. Liang, M. Hada, M. Ehara, K. Toyota, H. N. J. Hasegawa, M. Ishida, T. Nakajima, Y. Honda, O. Kitao, J. E. P. T. Vreven, K. Throssell, J. A. Montgomery, Jr., K. N. K. F. Ogliaro, M. J. Bearpark, J. J. Heyd, E. N. Brothers, J. N. V. N. Staroverov, T. A. Keith, R. Kobayashi, S. S. I. K. Raghavachari, A. P. Rendell, J. C. Burant, R. C. J. Tomasi, M. Cossi, J. M. Millam, M. Klene, C. Adamo, O. F. J. W. Ochterski, R. L. Martin, K. Morokuma and 2019. J. B. Foresman, and D. J. Fox, Gaussian, Inc., Wallingford CT, .

2 N. A. Beare and J. F. Hartwig, *J. Org. Chem.*, 2002, **67**, 541–555.

3 Y. Jiang, N. Wu, H. Wu and M. He, *Synlett*, 2005, 2731–2734.

4 H. A. P. De Jongh, C. R. H. I. De Jonge and W. J. Mijs, *J. Org. Chem.*, 1971, **36**, 3160–3168.

5 W. H. Hao, P. Yan, G. Li and Z. Y. Wang, *Dye. Pigment.*, 2014, **111**, 145–155.

6 M. K. Beyer, *J. Chem. Phys.*, 2000, **112**, 7307–7312.
